# Synthesis, 3D-QSAR and Molecular Docking Study of Nopol-Based 1,2,4-Triazole-Thioether Compounds as Potential Antifungal Agents

**DOI:** 10.3389/fchem.2021.757584

**Published:** 2021-10-25

**Authors:** Xiu Wang, Wengui Duan, Guishan Lin, Baoyu Li, Ming Chen, Fuhou Lei

**Affiliations:** ^1^ School of Chemistry and Chemical Engineering, Guangxi University, Nanning, China; ^2^ Guangxi Key Laboratory of Chemistry and Engineering of Forest Products, Nanning, China

**Keywords:** *β*-pinene, nopol, 1, 2, 4-triazole-thioether, 3D-QSAR, molecular docking

## Abstract

Cytochrome *bc*_1_ complex is an important component of cellular respiratory chain, and it is also an important target enzyme to inhibit the growth of plant pathogens. Using cytochrome *bc*_1_ complex as the target enzyme, twenty-three novel nopol-based 1,2,4-triazole-thioether compounds were designed and synthesized from natural preponderant resource *β*-pinene, and their structures were confirmed by FT-IR, NMR, ESI-MS and elemental analysis. The *in vitro* antifungal activity of the target compounds **5a-5w** was preliminarily evaluated against eight plant pathogens at the concentration of 50 µg/ml. The bioassay results showed that the target compounds exhibited the best antifungal activity against *Physalospora piricola*, in which compounds **5b** (R= *o*-CH_3_ Ph), **5e** (R= o-OCH_3_ Ph), **5h** (R= *o*-F Ph), **5m** (R= *o*-Br Ph), **5o** (R= *m,m*-OCH_3_ Ph), and **5r** (R= *p*-OH Ph) had inhibition rates of 91.4, 83.3, 86.7, 83.8, 91.4 and 87.3%, respectively, much better than that of the positive control chlorothalonil. Also, compound **5a** (R= Ph) had inhibition rate of 87.9% against *Rhizoeotnia solani*, and compound **5b** (R= *o*-CH_3_ Ph) had inhibition rates of 87.6 and 89% against *Bipolaris maydis* and *Colleterichum orbicala*, respectively. In order to develop novel and promising antifungal compounds against *P. piricola*, the analysis of three-dimensional quantitative structure-activity relationship (3D-QSAR) was carried out using the CoMFA method on the basis of their antifungal activity data, and a reasonable and effective 3D-QSAR model (*r* ^*2*^ = 0.944, *q* ^*2*^ = 0.685) has been established. In addition, the theoretical study of molecular docking revealed that the target compounds could bind to and interact with the site of cytochrome *bc*_1_ complex.

## Introduction

Plant diseases caused by phytopathogens have always been one of the main reasons for crop yield reduction, and the use of fungicides is the most critical method to effectively control crop diseases in agriculture (Wang et al., 2017). However, the frequent use and misuse of many traditional pesticides have caused environmental pollution, pesticide residual toxicity, and the emergence of resistant phytopathogenic fungi isolates (Jiao et al., 2021; Qi et al., 2021). Therefore, the development of novel effective antifungal agents is of great significance.

Cytochrome *bc*_1_ complex (EC 1.10.2.2), also known as complex III, is an important component of cellular respiratory chain, as well as a target enzyme used in the development of fungicides owing to its ability to inhibit the growth of plant pathogens (Chen et al., 2016; Cheng et al., 2018). At present, about twenty cytochrome *bc*_1_ complex fungicides have been successfully employed in the world market and more are still being developed according to the statistics from the Fungicide Resistance Action Committee (FRAC, 2021) including flufenoxystrobin, fluoxastrobin, famoxadone, pyrametostrobin and so on (http://www.frac.info/(accessed Aug 12, 2021). These compounds inhibit mitochondrial respiration of plant pathogens by binding at the Qo site of a membrane-bound homodimeric cytochrome *bc*_1_ complex and blocking the generation of adenosine triphosphate (ATP), leading to the inhibition of the energy production which is essential for survival (Wang et al., 2018; Zhu et al., 2019). Therefore, cytochrome *bc*_1_ complex inhibitors have been widely investigated by a large number of chemists to discover novel fungicides. In the past decade, some kinds of potential cytochrome *bc*_1_ complex inhibitors were designed and synthesized by Yang research team and some target compounds exhibited good *in vitro* inhibitory activities against downy mildew and powdery mildew (Wang et al., 2011; Hao et al., 2012; Hao et al., 2015).

Turpentine oil, with a productivity of around 330,000 tons for the most recent decades, is a natural renewable biomass resource which can be obtained by the steam distillation of the oleoresin exudate from living pine trees (García et al., 2020). It is one of the most widely produced secondary metabolites of plants, and its two major components are *α*-pinene and *β*-pinene, which have received much attention in recent years due to their various biological activities (Nie et al., 2014). The content of *β*-pinene in turpentine oil of *Pinus elliottii* can be as high as about 30% (Xu et al., 1992). Nopol, in which molecular scaffold of the natural *α*-pinene is maintained, can be prepared by the Prins reaction of *β*-pinene with paraformaldehyde under catalysis of Lewis acid (Yadav and Jasra, 2006; Jadhav et al., 2010; Vrbková et al., 2020). Nopol and its derivatives exhibited a broad spectrum of biological activities, such as antifungal (Chen et al., 2017; Jin et al., 2017; Feng et al., 2019; Chen et al., 2021), repellent (Han et al., 2008), antifeedant (Han et al., 2007), and treatment of diabetes and gastrointestinal irritable syndrome activities (He et al., 2008; Majouli et al., 2017). Herein, nopol deserves further study for agrochemical or pharmaceutical uses based on its bioactive property and chemical reactivity.

On the other hand, 1,2,4-triazole, as an important kind of heterocyclic nitrogen compounds, is a versatile lead molecule and usually employed to design potential bioactive agents whether in the field of pesticides or medicine. This molecule and its derivatives have received considerable attention owing to a wide range of biological activities, such as anticancer (Li et al., 2016; El-Sherief et al., 2018; Hou et al., 2019), antibacterial (Gao et al., 2019; Shi et al., 2020), anti-inflammatory (Abdel-Aziz et al., 2014), antiviral (Chen et al., 2019), antifungal (Xu et al., 2011), antitubercular (Karczmarzyk et al., 2020) and other biological activities (Mustafa et al., 2019; Liao et al., 2020; Peng et al., 2021). Our research group has reported the synthesis of two series of 1,2,4-triazole derivatives with potent antifungal activities (Lin et al., 2017; Lin et al., 2018).

In continuation of our interest in developing natural product-based bioactive compounds (Lin et al., 2017; Wang X. et al., 2018; Lin et al., 2018; Kang et al., 2019; Liu et al., 2020; Zhu et al., 2020; Chen et al., 2021; He et al., 2021), a series of novel nopol derivatives containing 1,2,4-triazole and thioether moieties were designed and synthesized by the strategy of molecular docking-based virtual screening based on the crystal structure of cytochrome *bc*_1_ complex. In addition, a three-dimensional structure-activity quantitative relationship (3D-QSAR) model was established by the comparative molecular field analysis (CoMFA) method.

## Materials and Methods

### General

All other materials and reagents were purchased from commercial suppliers and used as received. *β*-Pinene (GC purity 98%) was provided by Jiangxi Xuesong Natural Medicinal Oil Co., Ltd. 5-Substituted-1,2,4-triazole-3-thiones were prepared by our laboratory according to the literature (Liu et al., 2012). The GC analysis was performed on an Agilent 6890 GC equipped with an HP-1 (30 m, 0.530 mm, 0.88 µm) column. NMR spectra were recorded using tetramethylsilane (TMS) as the internal standard and deuterated chloroform (CDCl_3_) as a solvent on a Bruker Avance III HD 600 MHz spectrometer. Mass spectra were obtained by means of the electrospray ionization (ESI) method on TSQ Quantum Access MAX HPLC-MS instrument. The IR spectra were recorded by employing a Nicolet iS50 FT-IR spectrometer using the KBr pellet method. Elemental analyses were measured using a PE 2400 II elemental analyzer.

### General Procedure for Nopol 2

A mixture of *β*-pinene **1** (300 ml, 1.90 mol) and paraformaldehyde (85.68 g, 0.95 mol) was magnetically stirred under solvent-free condition. When the mixture was heated to 75°C, a catalytic quantity of anhydrous ZnCl_2_ was added to the reaction system. Afterwards, the reaction mixture was stirred at 75°C for 1 h, and then continuously stirred at 110°C for 10 h. When the reaction was completed, the reaction mixture was cooled to room temperature. Then, the organic layer was separated, washed three times with deionized water, and dried over anhydrous Na_2_SO_4_. The crude product was further purified by vacuum distillation to obtain nopol **2** as a colorless transparent liquid in the yield of 70% at 60–70°C/1,333 Pa (GC purity 94.5%). ^1^H NMR (600 MHz, CDCl_3_) *δ* (ppm): 5.36–5.35 (1H, m), 3.62–3.58 (m, 2H), 2.40 (1H, dt, *J* = 8.6, 5.6 Hz), 2.32–2.21 (4H, m), 2.14–2.10 (1H, m), 2.05 (1H, td, *J* = 5.6, 1.4 Hz), 1.51 (1H, s), 1.29 (3H, s), 1.16 (1H, d, *J* = 8.6 Hz), 0.86 (3H, s); ^13^C NMR (150 MHz, CDCl_3_) *δ* (ppm): 144.72, 119.38, 59.98, 45.61, 40.72, 40.22, 37.90, 31.76, 31.39, 26.25, 21.19; IR (KBr) *v*: 3,370, 3,025, 2,991, 2,914, 2,831, 1,468, 1,381, 1,368, 1,046 cm^−1^.

### General Procedure for Nopyl Chloroacetate 3

A solution of chloroacetyl chloride (8.470 g, 0.075 mol) in dry DCM (10 mL) was added slowly to a mixture of nopol (8.313 g, 0.05 mol) and triethylamine (5.059 g, 0.05 mol) in dry DCM (20 mL) with ice-bath cooling. The reaction process was monitored by TLC. Upon completion, saturated aqueous NaHCO_3_ (5 mL) was added to destroy the unreacted acyl chloride. Then, the organic layer was separated, washed with deionized water three times, dried over Na_2_SO_4_, and concentrated under reduced pressure. The crude product was further purified by silica gel chromatography (petroleum ether/ethyl acetate = 5:1, v/v) to obtain the intermediate **3**. ^1^H NMR (600 MHz, CDCl_3_) *δ* (ppm): 5.34–5.31 (1H, m), 4.27–4.17 (2H, m), 4.06 (2H, s), 2.42–2.32 (3H, m), 2.30–2.19 (2H, m), 2.11–2.10 (1H, m), 2.06 (1H, td, *J* = 5.7, 1.2 Hz), 1.29 (3H, s), 1.16 (1H, d, *J* = 8.6 Hz), 0.84 (3H, s); ^13^C NMR (150 MHz, CDCl_3_) *δ* (ppm): 167.28, 143.54, 119.26, 64.44, 45.61, 40.90, 40.67, 38.01, 35.71, 31.64, 31.34, 26.25, 21.09; IR (KBr) *v*: 3,029, 2,989, 2,917, 2,835, 1,739, 1,475, 1,181 cm^−1^.

### General Procedure for the Title Compounds 5a-5w

The intermediate **4** (2.0 mmol) and sodium acetate trihydrate (0.272 g, 2.0 mmol) were dissolved in 15 mL mixed solvent of ethanol and water (ethanol/water = 2:1, v/v). Then, the mixture was stirred at 45°C for 2 h. Afterwards, a solution of nopyl chloroacetate **3** (0.484 g, 2.0 mmol) in 5 mL EtOH was slowly added to the mixture and continuously refluxed for 8 h. Upon completion of the reaction, the mixture was concentrated in vacuum. Then, the crude product was poured into saturated sodium bicarbonate solution, and the mixture was extracted with dichloromethane (3 × 20 mL). The organic layer was washed with saturated NaCl solution three times, dried over anhydrous sodium sulfate, and purified by silica gel column chromatography (petroleum ether/ethyl acetate = 1:1, v/v) to afford the target compounds **5a-5w**.

2-(6,6-Dimethylbicyclo[3.1.1]hept-2-en-2-yl)ethyl2-((4-methyl-5-phenyl-4*H*-1,2,4-triazol-3-yl)thio)acetate (**5a**). Pale yellow liquid, yield: 63.2%; ^1^H NMR (600 MHz, CDCl_3_) *δ* (ppm): 7.67–7.62 (2H, m), 7.54–7.50 (3H, m), 5.32–5.29 (1H, m), 4.23–4.12 (2H, m), 4.10 (2H, s), 3.68 (3H, s), 2.37 (1H, dt, *J* = 8.6, 5.6 Hz), 2.32 (2H, t, *J* = 7.0 Hz), 2.28–2.18 (2H, m), 2.11–2.07 (1H, m), 2.04 (1H, td, *J* = 5.7, 1.3 Hz), 1.28 (3H, s), 1.14 (1H, d, *J* = 8.6 Hz), 0.82 (3H, s); ^13^C NMR (150 MHz, CDCl_3_) *δ* (ppm): 168.45, 156.18, 150.52, 143.60, 130.18, 128.94, 128.63, 126.96, 119.15, 64.30, 45.63, 40.68, 38.01, 35.75, 35.62, 31.83, 31.64, 31.35, 26.26, 21.13; IR (KBr) *v*: 3,031, 2,987, 2,914, 2,831, 1,735, 1,602, 1,572, 1,470, 685 cm^−1^; ESI-MS m/z: Calcd for C_22_H_27_N_3_O_2_S [M+H]^+^: 398.18, Found: 398.12; Anal. Calcd for C, 66.47; H, 6.85; N, 10.57; Found: C, 66.46; H, 6.83; N, 10.55.

2-(6,6-Dimethylbicyclo[3.1.1]hept-2-en-2-yl)ethyl2-((4-methyl-5-(2-methylphenyl)-4*H*-1,2,4-triazol-3-yl)thio)acetate (**5b**). Pale yellow liquid, yield: 65.3%; ^1^H NMR (600 MHz, CDCl_3_) *δ* (ppm): 7.44–7.40 (1H, m), 7.37–7.29 (3H, m), 5.33–5.30 (1H, m), 4.21–4.15 (2H, m), 4.12 (2H, s), 3.43 (3H, s), 2.38 (1H, dt, *J* = 8.6, 5.6 Hz), 2.32 (2H, t, *J* = 7.0 Hz), 2.29–2.18 (5H, m), 2.11–2.09 (1H, m), 2.05 (1H, td, *J* = 5.6, 1.4 Hz), 1.28 (3H, s), 1.15 (1H, d, *J* = 8.6 Hz), 0.83 (3H, s); ^13^C NMR (150 MHz, CDCl_3_) *δ* (ppm): 168.45, 155.85, 149.63, 143.59, 138.34, 130.69, 130.44, 130.23, 126.41, 126.01, 119.15, 64.29, 45.63, 40.68, 38.01, 35.76, 35.53, 31.64, 31.35, 30.87, 26.26, 21.14, 19.70; IR (KBr) *v*: 3,027, 2,987, 2,920, 2,831, 1,739, 1,606, 1,533, 1,466, 700 cm^−1^; ESI-MS m/z: Calcd for C_23_H_29_N_3_O_2_S [M+H]^+^: 412.20, Found: 412.12; Anal. Calcd for C, 67.12; H, 7.10; N, 10.21; Found: C, 67.11; H, 7.09; N, 10.20.

2-(6,6-Dimethylbicyclo[3.1.1]hept-2-en-2-yl)ethyl2-((4-methyl-5-(3-methylphenyl)-4*H*-1,2,4-triazol-3-yl)thio)acetate (**5c**). Pale yellow liquid, yield: 71.3%; ^1^H NMR (600 MHz, CDCl_3_) *δ* (ppm): 7.47 (1H, s), 7.41–7.37 (2H, m), 7.33–7.29 (1H, m), 5.30–5.29 (1H, m), 4.21–4.15 (2H, m), 4.08 (2H, s), 3.66 (3H, s), 2.42 (3H, s), 2.36 (1H, dt, *J* = 8.6, 5.6 Hz), 2.30 (2H, t, *J* = 7.0 Hz), 2.26–2.17 (2H, m), 2.08–2.04 (1H, m), 2.03 (1H, td, *J* = 5.7, 1.1 Hz), 1.26 (3H, s), 1.12 (1H, d, *J* = 8.6 Hz), 0.81 (3H, s); ^13^C NMR (150 MHz, CDCl_3_) δ (ppm): 168.46, 156.33, 150.36, 143.60, 138.85, 130.94, 129.40, 128.74, 126.84, 125.53, 119.14, 64.28, 45.62, 40.68, 38.00, 35.75, 35.66, 31.81, 31.63, 31.35, 26.25, 21.41, 21.12; IR (KBr) *v*: 3,027, 2,987, 2,918, 2,833, 1,733, 1,612, 1,589, 1,475, 695 cm^−1^; ESI-MS m/z: Calcd for C_23_H_29_N_3_O_2_S [M+H]^+^: 412.20, Found: 412.14; Anal. Calcd for C, 67.12; H, 7.10; N, 10.21; Found: C, 67.10; H, 7.08; N, 10.20.

2-(6,6-Dimethylbicyclo[3.1.1]hept-2-en-2-yl)ethyl2-((4-methyl-5-(4-methylphenyl)-4*H*-1,2,4-triazol-3-yl)thio)acetate (**5d**). Pale yellow liquid, yield: 75.8%; ^1^H NMR (500 MHz, CDCl_3_) *δ* (ppm): 7.53 (2H, d, *J* = 8.1 Hz), 7.32 (2H, d, *J* = 8.0 Hz), 5.32–5.28 (1H, m), 4.21–4.14 (2H, m), 4.09 (2H, s), 3.66 (3H, s), 2.44 (3H, s), 2.37 (1H, dt, *J* = 8.6, 5.6 Hz), 2.31 (2H, t, *J* = 7.0 Hz), 2.28–2.17 (2H, m), 2.09–2.05 (1H, m), 2.04 (1H, td, *J* = 5.6, 1.3 Hz), 1.28 (3H, s), 1.14 (1H, d, *J* = 8.6 Hz), 0.82 (3H, s); ^13^C NMR (125 MHz, CDCl_3_) *δ* (ppm): 168.47, 156.25, 150.26, 143.59, 140.38, 129.60, 128.49, 124.02, 119.13, 77.30, 77.04, 76.79, 64.28, 45.62, 40.68, 38.00, 35.74, 35.65, 31.80, 31.63, 31.34, 26.25, 21.45, 21.12; IR (KBr) *v*: 3,029, 2,985, 2,916, 2,831, 1,739, 1,620, 1,475, 697 cm^−1^; ESI-MS m/z: Calcd for C_23_H_29_N_3_O_2_S [M+H]^+^: 412.20, Found: 412.13; Anal. Calcd for C, 67.12; H, 7.10; N, 10.21; Found: C, 67.10; H, 7.08; N, 10.19.

2-(6,6-Dimethylbicyclo[3.1.1]hept-2-en-2-yl)ethyl2-((4-methyl-5-(2-methoxyphenyl)-4*H*-1,2,4-triazol-3-yl)thio)acetate (**5e**). Pale yellow liquid, yield: 62.2%; ^1^H NMR (500 MHz, CDCl_3_) *δ* (ppm): 7.54–7.48 (2H, m), 7.09 (1H, td, *J* = 7.5, 0.8 Hz), 7.04–7.00 (1H, m), 5.33–5.29 (1H, m), 4.23–4.15 (2H, m), 4.12 (2H, s), 3.84 (3H, s), 3.46 (3H, s), 2.38 (1H, dt, *J* = 8.5, 5.6 Hz), 2.35–2.30 (2H, m), 2.29–2.18 (2H, m), 2.10–2.08 (1H, m), 2.05 (1H, td, *J* = 5.6, 1.3 Hz), 1.28 (3H, s), 1.15 (1H, d, *J* = 8.6 Hz), 0.83 (3H, s); ^13^C NMR (125 MHz, CDCl_3_) *δ* (ppm): 168.52, 157.23, 154.67, 149.78, 143.62, 132.28, 132.15, 121.12, 119.13, 116.15, 111.06, 64.24, 55.56, 45.63, 40.69, 38.01, 35.75, 35.49, 31.64, 31.35, 31.13, 26.26, 21.13; IR (KBr) *v*: 3,027, 2,984, 2,912, 2,833, 1,736, 1,608, 1,584, 1,475, 675 cm^−1^; ESI-MS m/z: Calcd for C_23_H_29_N_3_O_3_S [M+H]^+^: 428.19, Found: 428.13; Anal. Calcd for C, 64.61; H, 6.84; N, 9.83; Found: C, 64.59; H, 6.83; N, 9.81.

2-(6,6-Dimethylbicyclo[3.1.1]hept-2-en-2-yl)ethyl2-((4-methyl-5-(3-methoxyphenyl)-4*H*-1,2,4-triazol-3-yl)thio)acetate (**5f**). Pale yellow liquid, yield: 65.9%; ^1^H NMR (600 MHz, CDCl_3_) *δ* (ppm): 7.42 (1H, t, *J* = 8.0 Hz), 7.22 (1H, s), 7.18 (1H, d, *J* = 7.5 Hz), 7.05 (1H, dd, *J* = 8.3, 2.4 Hz), 5.32–5.29 (1H, m), 4.20–4.15 (2H, m), 4.10 (2H, s), 3.87 (3H, s), 3.68 (3H, s), 2.37 (1H, dt, *J* = 8.5, 5.6 Hz), 2.32 (2H, t, *J* = 7.0 Hz), 2.28–2.18 (2H, m), 2.10–2.08 (1H, m), 2.05 (1H, dd, *J* = 10.1, 4.6 Hz), 1.28 (3H, s), 1.14 (1H, d, *J* = 8.6 Hz), 0.82 (3H, s); ^13^C NMR (150 MHz, CDCl_3_) *δ* (ppm): 168.42, 159.88, 156.04, 155.76, 150.56, 143.59, 129.95, 128.09, 120.62, 119.14, 116.21, 114.05, 64.30, 55.46, 45.62, 40.68, 38.01, 35.75, 35.60, 31.87, 31.63, 31.35, 26.25, 21.12; IR (KBr) *v*: 3,033, 2,983, 2,912, 2,833, 1,737, 1,610, 1,583, 1,481, 693 cm^−1^; ESI-MS m/z: Calcd for C_23_H_29_N_3_O_3_S [M+H]^+^: 428.19, Found: 428.27; Anal. Calcd for C, 64.61; H, 6.84; N, 9.83; Found: C, 64.60; H, 6.83; N, 9.82.

2-(6,6-Dimethylbicyclo[3.1.1]hept-2-en-2-yl)ethyl2-((4-methyl-5-(4-methoxyphenyl)-4*H*-1,2,4-triazol-3-yl)thio)acetate (**5g**). Pale yellow liquid, yield: 71.2%; ^1^H NMR (600 MHz, CDCl_3_) *δ* (ppm): 7.58 (2H, d, *J* = 8.3 Hz), 7.03 (2H, d, *J* = 8.4 Hz), 5.31–5.29 (1H, m), 4.20–4.13 (2H, m), 4.08 (2H, s), 3.88 (3H, s), 3.66 (3H, s), 2.37 (1H, dt, *J* = 8.4, 5.6 Hz), 2.31 (2H, t, *J* = 7.0 Hz), 2.30–2.18 (2H, m), 2.10–2.08 (1H, m), 2.05 (1H, dd, *J* = 10.7, 5.2 Hz), 1.28 (3H, s), 1.14 (1H, d, *J* = 8.6 Hz), 0.82 (3H, s); ^13^C NMR (150 MHz, CDCl_3_) *δ* (ppm): 168.48, 161.01, 156.07, 150.08, 143.59, 130.07, 119.24, 119.13, 114.37, 64.27, 55.41, 45.62, 40.68, 38.01, 35.75, 35.68, 31.79, 31.63, 31.35, 26.26, 21.12; IR (KBr) *v*: 3,035, 2,983, 2,927, 2,831, 1,737, 1,612, 1,577, 1,479, 722 cm^−1^; ESI-MS m/z: Calcd for C_23_H_29_N_3_O_3_S [M+H]^+^: 428.19, Found: 428.28; Anal. Calcd for C, 64.61; H, 6.84; N, 9.83; Found: C, 64.60; H, 6.82; N, 9.81.

2-(6,6-Dimethylbicyclo[3.1.1]hept-2-en-2-yl)ethyl2-((4-methyl-5-(2-fluorophenyl)-4*H*-1,2,4-triazol-3-yl)thio)acetate (**5h**). Pale yellow liquid, yield: 68.2%; ^1^H NMR (500 MHz, CDCl_3_) *δ* (ppm): 7.65 (1H, td, *J* = 7.4, 1.8 Hz), 7.55 (1H, td, *J* = 7.3, 1.8 Hz), 7.32 (1H, td, *J* = 7.6, 1.0 Hz), 7.26–7.20 (1H, m), 5.33–5.29 (1H, m), 4.24–4.14 (2H, m), 4.12 (2H, s), 3.57 (3H, s), 2.37 (1H, dt, *J* = 8.6, 5.6 Hz), 2.33 (2H, t, *J* = 7.2 Hz), 2.30–2.17 (2H, m), 2.12–2.07 (1H, m), 2.05 (1H, td, *J* = 5.6, 1.4 Hz), 1.28 (3H, s), 1.14 (1H, d, *J* = 8.6 Hz), 0.83 (3H, s); ^13^C NMR (125 MHz, CDCl_3_) δ (ppm): 168.36, 160.72, 158.73, 152.29, 150.76, 143.59, 132.70, 132.63, 132.28, 132.26, 124.98, 124.95, 119.14, 116.15, 115.98, 115.23, 115.12, 64.31, 45.62, 40.68, 38.01, 35.74, 35.47, 31.63, 31.35, 31.27, 26.26, 21.12; IR (KBr) *v*: 3,031, 2,987, 2,914, 2,831, 1,735, 1,602, 1,572, 1,468, 685 cm^−1^; ESI-MS m/z: Calcd for C_22_H_26_FN_3_O_2_S [M+H]^+^: 416.17, Found: 416.10; Anal. Calcd for C, 63.59; H, 6.31; N, 10.11; Found:C, 63.57; H, 6.30; N, 10.10.

2-(6,6-Dimethylbicyclo[3.1.1]hept-2-en-2-yl)ethyl2-((4-methyl-5-(3-fluorophenyl)-4*H*-1,2,4-triazol-3-yl)thio)acetate (**5i**). Pale yellow liquid, yield: 71.2%; ^1^H NMR (500 MHz, CDCl_3_) *δ* (ppm): 7.50 (1H, td, *J* = 8.0, 5.7 Hz), 7.46–7.42 (1H, m), 7.41–7.37 (1H, m), 7.22 (1H, tdd, *J* = 8.4, 2.6, 1.0 Hz), 5.32–5.29 (1H, m), 4.23–4.12 (2H, m), 4.10 (2H, s), 3.70 (3H, s), 2.37 (1H, dt, *J* = 8.6, 5.6 Hz), 2.32 (2H, t, *J* = 7.1 Hz), 2.28–2.17 (2H, m), 2.11–2.07 (1H, m), 2.04 (1H, td, *J* = 5.6, 1.3 Hz), 1.28 (3H, s), 1.14 (1H, d, *J* = 8.6 Hz), 0.82 (3H, s); ^13^C NMR (125 MHz, CDCl_3_) *δ* (ppm): 168.34, 163.71, 161.74, 154.97, 151.01, 143.57, 130.74, 130.67, 128.91, 128.84, 124.24, 124.22, 119.15, 117.33, 117.16, 115.83, 115.65, 64.33, 45.62, 40.67, 38.00, 35.75, 35.50, 31.90, 31.63, 31.34, 26.25, 21.12; IR (KBr) *v*: 3,027, 2,981, 2,916, 2,831, 1,739, 1,616, 1,589, 1,479, 687 cm^−1^; ESI-MS m/z: Calcd for C_22_H_26_FN_3_O_2_S [M+H]^+^: 416.17, Found: 416.25; Anal. Calcd for C, 63.59; H, 6.31; N, 10.11; Found:C, 63.58; H, 6.29; N, 10.10.

2-(6,6-Dimethylbicyclo[3.1.1]hept-2-en-2-yl)ethyl2-((4-methyl-5-(4-fluorophenyl)-4*H*-1,2,4-triazol-3-yl)thio)acetate (**5j**). Pale yellow liquid, yield: 78.7%; ^1^H NMR (500 MHz, CDCl_3_) *δ* (ppm): 7.67–7.62 (2H, m), 7.25–7.19 (2H, m), 5.32–5.29 (1H, m), 4.23–4.12 (2H, m), 4.10 (2H, s), 3.67 (3H, s), 2.37 (1H, dt, *J* = 8.6, 5.6 Hz), 2.33 (2H, t, *J* = 7.1 Hz), 2.28–2.17 (2H, m), 2.11–2.07 (1H, m), 2.04 (1H, td, *J* = 5.6, 1.4 Hz), 1.28 (3H, s), 1.14 (1H, d, *J* = 8.6 Hz), 0.82 (3H, s); ^13^C NMR (125 MHz, CDCl_3_) δ (ppm): 168.39, 164.80, 162.80, 155.29, 150.64, 143.58, 130.72, 130.65, 123.14, 123.11, 119.15, 116.29, 116.11, 64.32, 45.62, 40.67, 38.00, 35.75, 35.60, 31.80, 31.63, 31.34, 26.25, 21.12; IR (KBr) *v*: 3,029, 2,985, 2,918, 2,833, 1,739, 1,610, 1,537, 1,483, 625 cm^−1^; ESI-MS m/z: Calcd for C_22_H_26_FN_3_O_2_S [M+H]^+^: 416.17, Found: 416.10; Anal. Calcd for C, 63.59; H, 6.31; N, 10.11; Found:C, 63.57; H, 6.29; N, 10.09.

2-(6,6-Dimethylbicyclo[3.1.1]hept-2-en-2-yl)ethyl2-((4-methyl-5-(2-chlorophenyl)-4*H*-1,2,4-triazol-3-yl)thio)acetate (**5k**). Pale yellow liquid, yield: 66.2%; ^1^H NMR (500 MHz, CDCl_3_) *δ* (ppm): 7.55–7.48 (3H, m), 7.42 (1H, td, *J* = 7.4, 1.4 Hz), 5.33–5.30 (1H, m), 4.24–4.15 (2H, m), 4.13 (2H, s), 3.50 (3H, s), 2.38 (1H, dt, *J* = 8.6, 5.6 Hz), 2.32 (2H, t, *J* = 7.2 Hz), 2.30–2.17 (2H, m), 2.12–2.07 (1H, m), 2.04 (1H, td, *J* = 5.6, 1.4 Hz), 1.28 (3H, s), 1.15 (1H, d, *J* = 8.6 Hz), 0.83 (3H, s); ^13^C NMR (125 MHz, CDCl_3_) *δ* (ppm): 168.36, 154.28, 150.32, 143.59, 134.22, 132.66, 131.98, 129.90, 127.27, 126.54, 119.15, 64.32, 45.63, 40.68, 38.01, 35.76, 35.48, 31.64, 31.35, 31.10, 26.26, 21.14; IR (KBr) *v*: 3,025, 2,983, 2,914, 2,831, 1,739, 1,600, 1,566, 1,470, 658 cm^−1^; ESI-MS m/z: Calcd for C_22_H_26_ClN_3_O_2_S [M+H]^+^: 432.14, Found: 432.08; Anal. Calcd for C, 61.17; H, 6.07; N, 9.73; Found: C, 61.15; H, 6.06; N, 9.72.

2-(6,6-Dimethylbicyclo[3.1.1]hept-2-en-2-yl)ethyl2-((4-methyl-5-(3-chlorophenyl)-4*H*-1,2,4-triazol-3-yl)thio)acetate (**5l**). Pale yellow liquid, yield: 69.2%; ^1^H NMR (600 MHz, CDCl_3_) *δ* (ppm): 7.67 (1H, t, *J* = 1.8 Hz), 7.55 (1H, dt, *J* = 7.4, 1.4 Hz), 7.52–7.45 (2H, m), 5.33–5.29 (1H, m), 4.20–4.11 (2H, m), 4.11 (2H, s), 3.70 (3H, s), 2.37 (1H, dt, *J* = 8.6, 5.6 Hz), 2.32 (2H, t, *J* = 7.0 Hz), 2.28–2.18 (2H, m), 2.12–2.07 (1H, m), 2.05 (1H, td, *J* = 5.6, 1.4 Hz), 1.28 (3H, s), 1.14 (1H, d, *J* = 8.6 Hz), 0.82 (3H, s); ^13^C NMR (150 MHz, CDCl_3_) *δ* (ppm): 168.35, 154.86, 151.03, 143.57, 135.00, 130.32, 130.26, 128.64, 126.66, 119.16, 64.34, 45.62, 40.67, 38.01, 35.75, 35.59, 31.90, 31.63, 31.35, 26.25, 21.12; IR (KBr) *v*: 3,025, 2,987, 2,914, 2,831, 1,741, 1,604, 1,572, 1,472, 689 cm^−1^; ESI-MS m/z: Calcd for C_22_H_26_ClN_3_O_2_S [M+H]^+^: 432.14, Found: 432.05; Anal. Calcd for C, 61.17; H, 6.07; N, 9.73; Found: C, 61.16; H, 6.05; N, 9.72.

2-(6,6-Dimethylbicyclo[3.1.1]hept-2-en-2-yl)ethyl2-((4-methyl-5-(2-bromophenyl)-4*H*-1,2,4-triazol-3-yl)thio)acetate (**5m**). Pale yellow liquid, yield: 75.2%; ^1^H NMR (500 MHz, CDCl_3_) *δ* (ppm): 7.72 (1H, d, *J* = 7.6 Hz), 7.50–7.46 (2H, m), 7.46–7.40 (1H, m), 5.34–5.29 (1H, m), 4.23–4.15 (2H, m), 4.13 (2H, s), 3.49 (3H, s), 2.38 (1H, dt, *J* = 8.6, 5.6 Hz), 2.33 (2H, t, *J* = 7.2 Hz) 2.29–2.18 (2H, m), 2.12–2.07 (1H, m), 2.05 (1H, td, *J* = 5.6, 1.4 Hz), 1.29 (3H, s), 1.15 (1H, d, *J* = 8.6 Hz), 0.83 (3H, s); ^13^C NMR (125 MHz, CDCl_3_) *δ* (ppm): 168.36, 155.37, 150.14, 143.59, 133.05, 132.72, 132.09, 128.72, 127.76, 123.93, 119.16, 64.31, 45.64, 40.69, 38.02, 35.77, 35.51, 31.65, 31.36, 31.14, 26.27, 21.15; IR (KBr) *v*: 3,027, 2,987, 2,916, 2,829, 1,737, 1,598, 1,564, 1,472, 650 cm^−1^; ESI-MS m/z: Calcd for C_22_H_26_BrN_3_O_2_S [M+H]^+^: 478.09, Found: 478.00; Anal. Calcd for C, 55.46; H, 5.50; N, 8.82; Found: C, 55.45; H, 5.49; N, 8.81.

2-(6,6-Dimethylbicyclo[3.1.1]hept-2-en-2-yl)ethyl2-((4-methyl-5-(3-bromophenyl)-4*H*-1,2,4-triazol-3-yl)thio)acetate (**5n**). Pale yellow liquid, yield: 59.5%; ^1^H NMR (500 MHz, CDCl_3_) *δ* (ppm): 7.82 (1H, t, *J* = 1.7 Hz), 7.66 (1H, ddd, *J* = 8.1, 1.9, 1.0 Hz), 7.61–7.57 (1H, m), 7.40 (1H, t, *J* = 7.9 Hz), 5.32–5.28 (1H, m), 4.22–4.13 (2H, m), 4.10 (2H, s), 3.69 (3H, s), 2.37 (1H, dt, *J* = 8.6, 5.6 Hz), 2.31 (2H, t, *J* = 7.0 Hz), 2.29–2.17 (2H, m), 2.10–2.08 (1H, m), 2.05 (1H, td, *J* = 5.6, 1.4 Hz), 1.28 (3H, s), 1.14 (1H, d, *J* = 8.6 Hz), 0.82 (3H, s); ^13^C NMR (125 MHz, CDCl_3_) *δ* (ppm): 168.34, 154.73, 151.03, 143.56, 133.25, 131.50, 130.46, 128.85, 127.10, 122.98, 119.16, 64.34, 45.62, 40.67, 38.01, 35.75, 35.59, 31.90, 31.64, 31.35, 26.26, 21.13; IR (KBr) *v*: 3,027, 2,981, 2,918, 2,829, 1,737, 1,600, 1,568, 1,475, 685 cm^−1^; ESI-MS m/z: Calcd for C_22_H_26_BrN_3_O_2_S [M+H]^+^: 478.09, Found: 478.01; Anal. Calcd for C, 55.46; H, 5.50; N, 8.82; Found: C, 55.44; H, 5.49; N, 8.80.

2-(6,6-Dimethylbicyclo[3.1.1]hept-2-en-2-yl)ethyl2-((5-(3,5-dimethoxyphenyl)-4-methyl-4*H*-1,2,4-triazol-3-yl)thio)acetate (**5o**). Pale yellow liquid, yield: 68.5%; ^1^H NMR (500 MHz, CDCl_3_) *δ* (ppm): 6.77 (2H, d, *J* = 2.3 Hz), 6.59 (1H, t, *J* = 2.3 Hz), 5.32–5.29 (1H, m), 4.21–4.12 (2H, m), 4.09 (2H, s), 3.85 (6H, s), 3.68 (3H, s), 2.37 (1H, dt, *J* = 8.6, 5.6 Hz), 2.32 (2H, t, *J* = 7.0 Hz), 2.28–2.17 (2H, m), 2.11–2.06 (1H, m), 2.05 (1H, td, *J* = 5.6, 1.4 Hz), 1.28 (3H, s), 1.14 (1H, d, *J* = 8.6 Hz), 0.82 (3H, s); ^13^C NMR (125 MHz, CDCl_3_) *δ* (ppm): 168.40, 161.03, 156.00, 150.61, 143.58, 128.49, 119.13, 106.70, 102.21, 64.30, 55.58, 45.62, 40.67, 38.00, 35.74, 35.58, 31.91, 31.63, 31.34, 26.25, 21.12; IR (KBr) *v*: 2,985, 2,917, 2,834, 1,735, 1,596, 1,525, 1,482, 688 cm^−1^; ESI-MS m/z: Calcd for C_24_H_31_N_3_O_4_S [M+H]^+^: 458.20, Found: 458.14; Anal. Calcd for C, 63.00; H, 6.83; N, 9.18; Found: C, 63.00; H, 6.81; N, 9.16.

2-(6,6-Dimethylbicyclo[3.1.1]hept-2-en-2-yl)ethyl2-((5-(2-amino-5-methylphenyl)-4-methyl-4*H*-1,2,4-triazol-3-yl)thio)acetate (**5p**). Pale yellow liquid, yield: 81.2%; ^1^H NMR (500 MHz, CDCl_3_) *δ* (ppm): 7.07 (1H, dd, *J* = 8.2, 1.6 Hz), 7.00 (1H, d, *J* = 1.6 Hz), 6.75 (1H, d, *J* = 8.2 Hz), 5.32–5.30 (1H, m), 4.21–4.11 (2H, m), 4.11 (2H, s), 3.62 (3H, s), 2.38 (1H, dt, *J* = 8.6, 5.6 Hz), 2.33 (2H, t, *J* = 7.2 Hz), 2.29 (3H, s), 2.25–2.18 (2H, m), 2.12–2.07 (1H, m), 2.05 (1H, td, *J* = 5.6, 1.6 Hz), 1.28 (3H, s), 1.15 (1H, d, *J* = 8.6 Hz), 0.83 (3H, s); ^13^C NMR (125 MHz, CDCl_3_) *δ* (ppm): 168.39, 154.73, 150.08, 144.20, 143.61, 131.94, 129.42, 126.80, 119.13, 116.79, 110.67, 64.31, 45.63, 40.68, 38.01, 35.75, 35.33, 31.91, 31.64, 31.35, 26.26, 21.13, 20.41; IR (KBr) *v*: 3,456, 3,350, 3,214, 3,025, 2,983, 2,918, 2,833, 1,739, 1,604, 1,508, 1,468, 681 cm^−1^; ESI-MS m/z: Calcd for C_23_H_30_N_4_O_2_S [M+H]^+^: 427.21, Found: 427.13; Anal. Calcd for C, 64.76; H, 7.09; N, 13.13; Found: C, 64.74; H, 7.08; N, 13.12.

2-(6,6-Dimethylbicyclo[3.1.1]hept-2-en-2-yl)ethyl2-((5-(3-amino-4-methylphenyl)-4-methyl-4*H*-1,2,4-triazol-3-yl)thio)acetate (**5q**). Pale yellow liquid, yield: 78.6%; ^1^H NMR (500 MHz, CDCl_3_) *δ* (ppm): 7.16 (1H, d, *J* = 7.7 Hz), 7.00 (1H, d, *J* = 1.6 Hz), 6.88 (1H, d, *J* = 7.6 Hz), 5.32–5.29 (1H, m), 4.21–4.13 (2H, m), 4.08 (2H, s), 3.65 (3H, s), 2.37 (1H, dt, *J* = 8.5, 5.6 Hz), 2.31 (2H, t, *J* = 7.0 Hz), 2.29–2.16 (5H, m), 2.11–2.08 (1H, m), 2.05 (1H, dd, *J* = 5.9, 1.4 Hz), 1.28 (3H, s), 1.14 (1H, d, *J* = 8.6 Hz), 0.82 (3H, s); ^13^C NMR (125 MHz, CDCl_3_) *δ* (ppm): 168.48, 156.46, 150.08, 145.22, 143.60, 130.75, 125.40, 124.51, 119.13, 118.22, 114.88, 64.27, 45.62, 40.68, 38.01, 35.75, 35.64, 31.83, 31.64, 31.35, 26.26, 21.13, 17.35; IR (KBr) *v*: 3,462, 3,354, 3,229, 3,025, 2,985, 2,918, 2,829, 1,737, 1,508, 1,475, 681 cm^−1^; ESI-MS m/z: Calcd for C_23_H_30_N_4_O_2_S [M+H]^+^: 427.21, Found: 427.14; Anal. Calcd for C, 64.76; H, 7.09; N, 13.13; Found: C, 64.75; H, 7.07; N, 13.11.

2-(6,6-Dimethylbicyclo[3.1.1]hept-2-en-2-yl)ethyl2-((4-methyl-5-(4-hydroxyphenyl)-4*H*-1,2,4-triazol-3-yl)thio)acetate (**5r**). Pale yellow liquid, yield: 74.6%; ^1^H NMR (500 MHz, CDCl_3_) *δ* (ppm): 9.98 (1H, s) 7.39–7.36 (2H, m), 6.95–6.91 (2H, m), 5.31–5.28 (1H, m), 4.22–4.11 (2H, m), 4.09 (2H, s), 3.64 (3H, s), 2.36 (1H, dt, *J* = 8.5, 5.6 Hz), 2.31 (2H, t, *J* = 7.2 Hz), 2.27–2.16 (2H, m), 2.11–2.05 (1H, m), 2.03 (1H, td, *J* = 5.6, 1.4 Hz), 1.27 (3H, s), 1.13 (1H, d, *J* = 8.6 Hz), 0.81 (3H, s); ^13^C NMR (125 MHz, CDCl_3_) *δ* (ppm): 168.39, 159.62, 156.53, 150.20, 143.55, 130.05, 119.16, 116.56, 116.48, 64.38, 45.60, 40.66, 38.00, 35.73, 35.53, 31.83, 31.63, 31.34, 26.25, 21.12; IR (KBr) *v*: 3,420, 2,954, 2,914, 2,881, 1,735, 1,614, 1,541, 1,477, 704 cm^−1^; ESI-MS m/z: Calcd for C_22_H_27_N_3_O_3_S [M+H]^+^: 414.18, Found: 414.09; Anal. Calcd for C, 63.90; H, 6.58; N, 10.16; Found: C, 63.89; H, 6.56; N, 10.14.

2-(6,6-Dimethylbicyclo[3.1.1]hept-2-en-2-yl)ethyl2-((4-methyl-5-(3-(trifluoromethyl)phenyl)-4*H*-1,2,4-triazol-3-yl)thio)acetate (**5s**). Pale yellow liquid, yield: 68.7%; ^1^H NMR (500 MHz, CDCl_3_) δ (ppm): 7.94 (1H, s), 7.86 (1H, d, *J* = 7.8 Hz), 7.79 (1H, d, *J* = 7.9 Hz), 7.67 (1H, t, *J* = 7.8 Hz), 5.32–5.29 (1H, m), 4.21–4.11 (2H, m), 4.11 (2H, s), 3.71 (3H, s), 2.36 (1H, dt, *J* = 8.5, 5.6 Hz), 2.33 (2H, t, *J* = 7.2 Hz), 2.28–2.17 (2H, m), 2.11–2.06 (1H, m), 2.04 (1H, td, *J* = 5.7, 1.0 Hz), 1.28 (3H, s), 1.13 (1H, d, *J* = 8.6 Hz), 0.82 (3H, s); ^13^C NMR (125 MHz, CDCl_3_) *δ* (ppm): 168.31, 154.79, 151.28, 143.56, 131.75, 129.61, 127.83, 126.90, 125.45, 124.67, 122.51, 119.16, 64.36, 45.62, 40.67, 38.00, 35.75, 35.58, 31.90, 31.63, 31.34, 26.24, 21.11; IR (KBr) *v*: 3,025, 2,985, 2,920, 2,835, 1,737, 1,593, 1,468, 700 cm^−1^; ESI-MS m/z: Calcd for C_23_H_26_F_3_N_3_O_2_S [M+H]^+^: 466.17, Found: 466.10; Anal. Calcd for C, 59.34; H, 5.63; N, 9.03; Found: C, 59.32; H, 5.613; N, 9.02.

2-(6,6-Dimethylbicyclo[3.1.1]hept-2-en-2-yl)ethyl2-((4-methyl-5-(furan-2-yl)-4*H*-1,2,4-triazol-3-yl)thio)acetate (**5t**). Pale yellow liquid, yield: 76.2%; ^1^H NMR (500 MHz, CDCl_3_) *δ* (ppm): 7.60 (1H, d, *J* = 1.6 Hz), 7.07 (1H, d, *J* = 3.5 Hz), 6.58 (1H, dd, *J* = 3.5, 1.8 Hz), 5.30–5.27 (1H, m), 4.21–4.11 (2H, m), 4.05 (2H, s), 3.85 (3H, s), 2.36 (1H, dt, *J* = 8.6, 5.6 Hz), 2.30 (2H, t, *J* = 7.2 Hz), 2.27–2.17 (2H, m), 2.11–2.06 (1H, m), 2.03 (1H, td, *J* = 5.6, 1.3 Hz), 1.27 (3H, s), 1.13 (1H, d, *J* = 8.6 Hz), 0.81 (3H, s); ^13^C NMR (125 MHz, CDCl_3_) *δ* (ppm): 168.32, 150.21, 148.36, 143.95, 143.57, 142.28, 119.12, 111.86, 111.79, 64.29, 45.61, 40.67, 37.99, 35.80, 35.72, 31.93, 31.62, 31.33, 26.25, 21.11; IR (KBr) *v*: 3,027, 2,981, 2,918, 2,831, 1,737, 1,614, 1,514, 1,447, 704 cm^−1^; ESI-MS m/z: Calcd for C_20_H_25_N_3_O_3_S [M+H]^+^: 388.16, Found: 388.24; Anal. Calcd for C, 61.99; H, 6.50; N, 10.84; Found: C, 61.97; H, 6.49; N, 10.82.

2-(6,6-Dimethylbicyclo[3.1.1]hept-2-en-2-yl)ethyl2-((4-methyl-5-(thiophen-2-yl)-4*H*-1,2,4-triazol-3-yl)thio)acetate (**5u**). Pale yellow liquid, yield: 69.2%; ^1^H NMR (500 MHz, CDCl_3_) *δ* (ppm): 7.52 (1H, dd, *J* = 5.1, 0.6 Hz), 7.50–7.46 (1H, m), 7.19 (1H, dd, *J* = 5.0, 3.8 Hz), 5.32–5.27 (1H, m), 4.22–4.11 (2H, m), 4.06 (2H, s), 3.78 (3H, s), 2.37 (1H, dt, *J* = 8.5, 5.6 Hz), 2.31 (2H, t, *J* = 7.1 Hz), 2.29–2.17 (2H, m), 2.11–2.06 (1H, m), 2.03 (1H, td, *J* = 5.7, 1.1 Hz), 1.28 (3H, s), 1.13 (1H, d, *J* = 8.6 Hz), 0.82 (3H, s); ^13^C NMR (125 MHz, CDCl_3_) *δ* (ppm): 168.36, 151.11, 150.52, 143.57, 128.39, 127.95, 127.79, 127.75, 119.14, 64.30, 45.61, 40.67, 38.00, 35.81, 35.74, 31.89, 31.63, 31.34, 26.25, 21.12; IR (KBr) *v*: 3,027, 2,985, 2,916, 2,831, 1,741, 1,566, 1,470, 712 cm^−1^; ESI-MS m/z: Calcd for C_20_H_25_N_3_O_2_S_2_ [M+H]^+^: 404.14, Found: 404.06; Anal. Calcd for C, 59.53; H, 6.24; N, 10.41; Found: C, 59.52; H, 6.22; N, 10.40.

2-(6,6-Dimethylbicyclo[3.1.1]hept-2-en-2-yl)ethyl2-((4-methyl-5-(pyridin-2-yl)-4*H*-1,2,4-triazol-3-yl)thio)acetate (**5v**). Pale yellow liquid, yield: 71.7%; ^1^H NMR (500 MHz, CDCl_3_) *δ* (ppm): 8.65 (1H, d, *J* = 4.8 Hz), 8.27 (1H, d, *J* = 8.0 Hz), 7.83 (1H, td, *J* = 7.8, 1.8 Hz), 7.35 (1H, ddd, *J* = 7.5, 4.9, 1.0 Hz), 5.32–5.27 (1H, m), 4.23–4.14 (2H, m), 4.12 (2H, s), 4.07 (3H, s), 2.36 (1H, dt, *J* = 8.6, 5.6 Hz), 2.31 (2H, t, *J* = 7.2 Hz), 2.27–2.17 (2H, m), 2.08–2.06 (1H, m), 2.04 (1H, td, *J* = 5.7, 1.3 Hz), 1.27 (3H, s), 1.13 (1H, d, *J* = 8.6 Hz), 0.82 (3H, s); ^13^C NMR (125 MHz, CDCl_3_) *δ* (ppm): 168.32, 153.59, 151.99, 148.70, 147.84, 143.62, 136.98, 123.98, 123.41, 119.09, 64.28, 45.62, 40.68, 38.00, 35.73, 35.25, 33.10, 31.62, 31.33, 26.25, 21.11; IR (KBr) *v*: 3,027, 2,983, 2,914, 2,831, 1,741, 1,589, 1,566, 1,481, 697 cm^−1^; ESI-MS m/z: Calcd for C_21_H_26_N_4_O_2_S [M+H]^+^: 399.18, Found: 399.25; Anal. Calcd for C, 63.29; H, 6.58; N, 14.06; Found: C, 63.28; H, 6.56; N, 14.05.

2-(6,6-Dimethylbicyclo[3.1.1]hept-2-en-2-yl)ethyl2-((5-butyl-4-methyl-4*H*-1,2,4-triazol-3-yl)thio)acetate (**5w**). Pale yellow liquid, yield: 68.1%; ^1^H NMR (500 MHz, CDCl_3_) *δ* (ppm): 5.30–5.27 (1H, m), 4.18–4.09 (2H, m), 3.98 (2H, s), 3.54 (3H, s), 2.75–2.69 (2H, m), 2.36 (1H, dt, *J* = 8.6, 5.6 Hz), 2.31 (2H, t, *J* = 7.2 Hz), 2.24–2.17 (2H, m), 2.09–2.07 (1H, m), 2.03 (1H, td, *J* = 5.6, 1.4 Hz), 1.75 (2H, dt, *J* = 15.4, 7.6 Hz), 1.48–1.39 (2H, m), 1.27 (3H, s), 1.13 (1H, d, *J* = 8.6 Hz), 0.96 (3H, t, *J* = 7.4 Hz), 0.81 (3H, s); ^13^C NMR (125 MHz, CDCl_3_) *δ* (ppm): 168.53, 156.45, 148.91, 143.58, 119.11, 64.20, 45.62, 40.67, 37.99, 35.86, 35.72, 31.62, 31.33, 30.16, 29.04, 26.25, 25.03, 22.37, 21.11, 13.70; IR (KBr) *v*: 2,985, 2,929, 2,833, 1,737, 1,516, 1,468, 677 cm^−1^; ESI-MS m/z: Calcd for C_20_H_31_N_3_O_2_S [M+H]^+^: 378.21, Found: 378.14; Anal. Calcd for C, 63.63; H, 8.28; N, 11.13; Found: C, 63.61; H, 8.27; N, 11.12.

### Antifungal Assay

All the target compounds were evaluated for their *in vitro* antifungal activities against eight plant fungi by the agar dilution method according to the literature (Su et al., 2013). Each tested compound was dissolved in acetone and diluted with Sorporl-144 (200 mg/L) as an emulsifier to prepare the solution with a concentration of 500 mg/L. The stock solution (1 mL) was added to the Potato-Sugar-Agar (PSA, 9 mL) culture medium. Then, mycelion dishes of 5 mm diameter were cut along the external edge of the mycelium was transferred to the center of flat containing the tested compound and put in equilateral triangular style in triplicate. After culturing 48 h at (24 ± 1) °C in the incubator, the expanded colony diameters of strains were measured and compared with that treated with aseptic distilled water, and then calculated the relative inhibition percentage. The corresponding aseptic distilled water without the test samples was served as a blank control and the commercial protective fungicide chlorothalonil was employed as the positive control. The test for each target compound was repeated three times. The inhibitory rate of the tested compound was calculated by the formula: inhibitory rate = [(the average extended mycelium diameter of the blank assay−the average extended mycelium diameter after treatment with emulsion)/the average extended mycelium diameter of the blank assay] × 100%.

### 3D-QSAR Study

**FIGURE 1:**
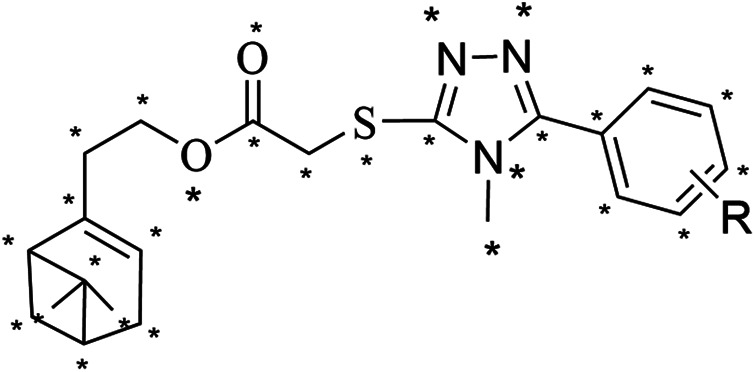
The asterisk skeleton of title compounds.

**FIGURE 2:**
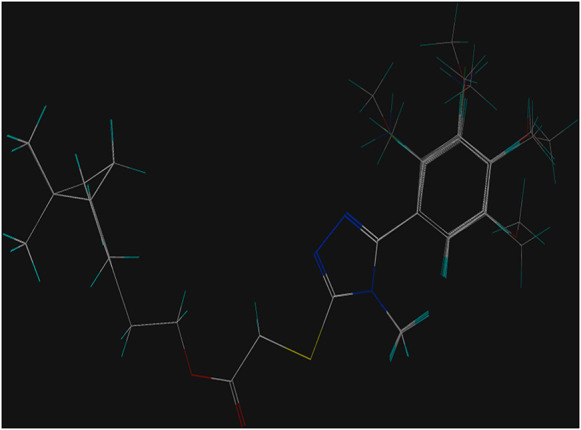
Superposed mode of the compounds.

The 3D-QSAR model was built using the CoMFA method of Sybyl-X 2.1.1 software to investigate the relationship between the antifungal activity and the compound structure according to our previous report (Zhao et al., 2021). The 3D structures of compounds **5a-5r** were built on the sketch module and minimized by the conjugate gradient method in the program based on the Tripos force field using termination convergence energy of 0.005 kcal/(mol*Å), a maximum of 1,000 iterations, and Gasteiger-Hückel charge as the parameters. The compound **5b** with the best activity against *P. piricola* was selected as the template compound, of which the atoms marked with an asterisk (Figure 1) were used as the common superimposed skeleton. 16 compounds using as the training set were aligned to form a superimposed model (Figure 2) and the remaining three compounds bearing aromatic R groups constitute the test set. Their inhibitor rates against *P. piricola* were converted to the active factor (AF) values by the formula:

AF = log {I/[(100 -I) × MW]}

where I was the inhibition rate at 50 µg/mL and MW was the molecular weight. The built 3D-QSAR model was checked by the partial least squares method. The modeling predictive capability was indicated by a correlation coefficient squared *r* ^*2*^, a cross-validated squared *q* ^*2*^, a standard deviation S, and a Fisher validation value F.

### Molecular Docking Study

To understand the binding interactions of the target compounds with the active site of cytochrome *bc*_1_ complex, the molecular docking procedures were carried out using AutoDock 4.2.6 software according to the reported paper (Lin et al., 2019; Du et al., 2020). The target protein PDB file for the cytochrome *bc*_1_ complex (PDB ID 1SQB, 2.69 Å in resolution) was downloaded from the RCSB PDB web (https://www.rcsb.org/). The site of the Azoxystrobin ligand was chosen as the docking domain. Before docking calculations, all of the other small molecules such as some ligands (except for Azoxystrobin ligand) and water molecules in the crystal were removed. The torsional bonds of each docked compound were automatically set by the AUTOTORS module. A grid map with a 60 × 60 × 60 point grid box (X = 69.364, Y = 57.986, Z = 168.988) with 0.375 Å spacing was selected around the site of the azoxystrobin ligand. The grid box was generated by applying default parameters. The docking calculations were performed using the Lamarckian genetic algorithm (GA) and the number of GA runs was set to 20 conformations. The lowest binding energy in the maximum cluster for the docked conformations was chosen as the representative binding energy. The binding energy between the docked compound and the enzyme was calculated using the AutoGrid program with a grid spacing of 0.375 Å and the Lamarckian genetic algorithm as a searching method. When the docking results were generated, the binding energies of all docked compounds were also automatically obtained in this program.

## Results and Discussion

### Chemistry

**SCHEME 1:**
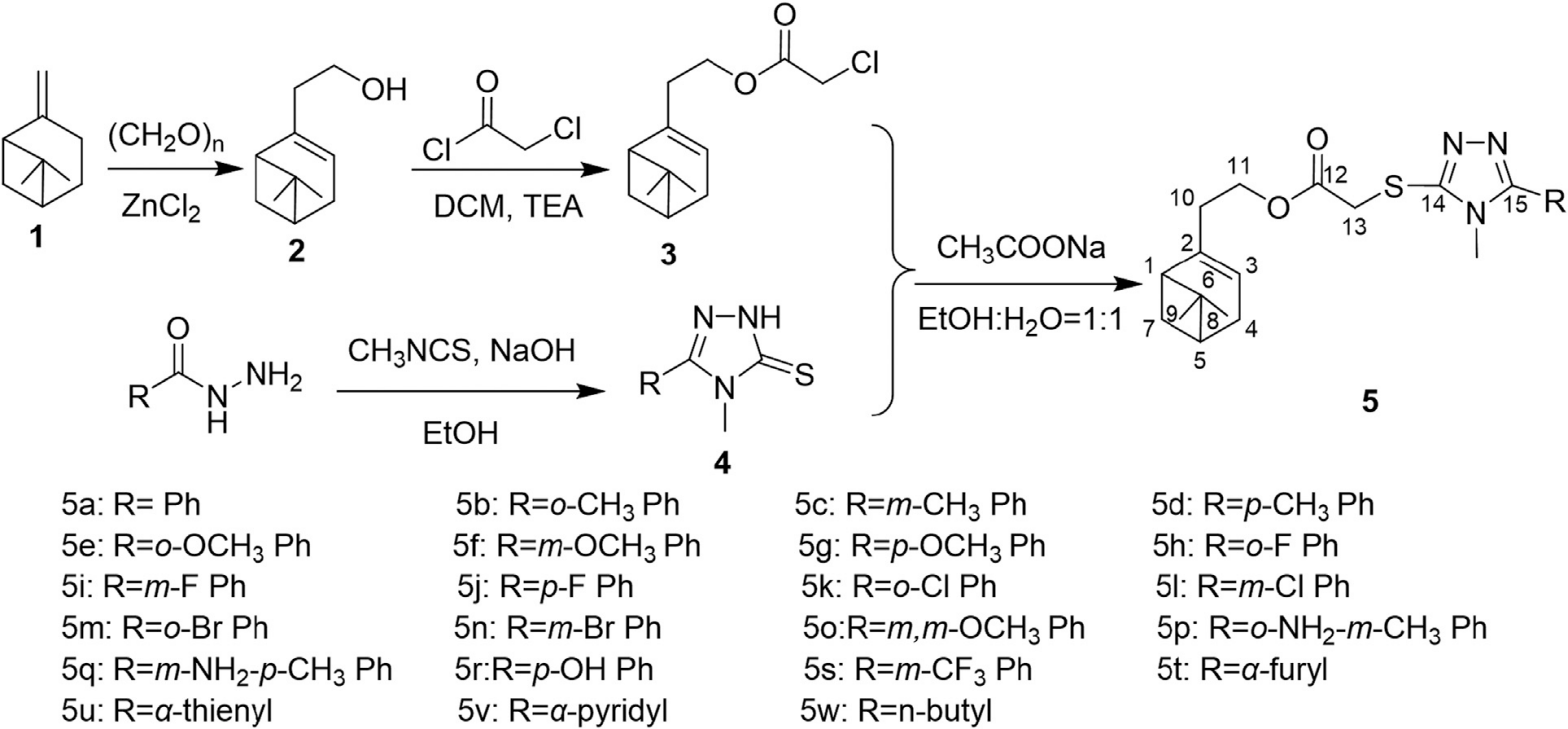
Synthesis of nopol-based 1,2,4-triazole-thioether compounds **5a-5w**.

The synthetic route of nopol-based 1,2,4-triazole-thioether compounds **5a-5w** was illustrated in Scheme 1. At first, nopol **2** was prepared by Prins reaction of *β*-pinene **1** with paraformaldehyde under catalysis of Lewis acid ZnCl_2_ (Yi et al., 2000). Then, nopyl chloroacetate **3** was prepared by alcoholysis reaction of chloroacetyl chloride with nopol **2** in a good yield (Huang et al., 2017), followed by nucleophilic substitution reaction of nopyl chloroacetate **3** with self-prepared 5-substituted-1,2,4-triazole-3-thiones **4** to afford a series of novel nopol-based 1,2,4-triazole-thioether compounds **5a-5w** (Liu et al., 2007).

The structures of all the synthesized compounds and the key intermediates **2** and **3** were characterized by FT-IR, ^1^H NMR, ^13^C NMR, ESI-MS, and elemental analysis, and the related spectra can be found in Supplementary Material. In the IR spectra of the target compounds, the weak absorption bands at about 3,025 cm^−1^ and about 1,610 cm^−1^ were attributed to the stretching vibrations of =C–H and C=C in the nopol moiety, respectively. The strong absorption bands at about 1,730 cm^−1^ and about 1,470 cm^−1^ were assigned to the stretching vibrations of C=O in the ester group and C=N in the 1,2,4-triazole moiety, respectively. Also, the absorption bands at 605–712 cm^−1^ revealed the presence of C-S-C in thioether moiety. In the ^1^H NMR spectra, the olefinic protons of nopol scaffold showed signals at about 5.33 ppm, and the other protons bonded to the saturated carbons of the nopol moiety displayed signals in the range of 0.81–2.37 ppm. The protons on the saturated carbon bonded to the S atom displayed signals at about 4.10 ppm. The ^13^C NMR spectra of all the target compounds showed peaks for the two olefinic carbons of the nopol moiety at about 143 and 119 ppm, respectively, and the unsaturated carbons in the triazole heterocycle and the benzene ring showed signals at 156–150 and 125–138 ppm, respectively. The carbons of C=O displayed the signals at 168 ppm and the saturated carbon bonded to the S atom displayed signals at about 35 ppm. Their molecular weights were confirmed by ESI-MS.

### *In Vitro* Antifungal Activity of Target Compounds

The antifungal activity of the target compounds **5a-5w** was evaluated using the agar dilution method against *Fusarium oxysporum* f. sp. *cucumerinum*, *Cercospora arachidicola*, *Physalospora piricola*, *Alternaria solani*, *Gibberella zeae*, *Rhizoeotnia solani*, *Bipolaris maydis*, and *Colleterichum orbicalare* at a concentration of 50 µg/mL, using the commercial antifungal drug chlorothalonil as positive control. The results were listed in Table 1.

**TABLE 1:** Antifungal activity of nopol-based 1,2,4-triazole- thioether compounds **5a-5w** at 50 µg/mL.

Compounds	Relative inhibition rate (%) against the fungi							
	*F.oxysporm* f. sp*. cucumerinum*	*C. arachidicola*	*P. piricola*	*A. solani*	*G. zeae*	*R. solani*	*B. myadis*	*C*. *orbicalare*
**5a** (R=Ph)	52.4	59.1	58.1	73.3	69.1	87.9	71.4	81.9
**5b** (R=*o*-CH_3_ Ph)	76.8	76.5	91.4	80.0	65.3	75.6	87.6	89.0
**5c** (R=*m*-CH_3_ Ph)	49.7	41.7	74.3	63.3	48.3	38.5	44.3	51.0
**5d** (R=*p*-CH_3_ Ph)	68.6	80.9	43.8	60.0	42.6	50.9	49.7	65.2
**5e** (R=*o*-OCH_3_ Ph)	65.9	72.2	83.3	63.3	52.1	75.6	60.5	81.9
**5f** (R=*m*-OCH_3_ Ph)	65.9	72.2	63.8	36.7	74.7	45.9	57.8	65.2
**5g** (R=*p*-OCH_3_ Ph)	71.4	72.2	43.8	50.0	55.8	57.0	60.5	79.5
**5h** (R=*o*-F Ph)	57.8	59.1	86.7	40.0	52.1	57.0	63.2	67.6
**5i** (R=*m*-F Ph)	65.9	59.1	67.6	43.3	50.2	63.2	47.0	58.1
**5j** (R=*p*-F Ph)	47.0	50.4	53.3	56.7	57.7	32.3	44.3	58.1
**5k** (R=*o*-Cl Ph)	49.7	54.8	75.7	53.3	69.1	58.3	49.7	60.5
**5l** (R=*m*-Cl Ph)	47.0	63.5	63.3	53.3	61.5	32.3	52.4	62.9
**5m** (R=*o*-Br Ph)	61.4	63.5	83.8	66.7	65.3	60.7	76.8	84.3
**5n** (R=*m*-Br Ph)	30.8	59.1	65.8	43.3	52.1	23.7	49.7	53.3
**5o** (R=*m*,*m*-OCH_3_ Ph)	52.4	67.8	91.4	63.3	61.5	38.5	63.2	65.2
**5p** (R=*o*-NH_2_-*m*-CH_3_ Ph)	49.7	50.4	67.6	66.7	61.5	60.7	74.1	79.5
**5q** (R=*m*-NH_2_-*p*-CH_3_ Ph)	60.5	63.5	73.8	36.7	46.4	57.0	49.7	70.0
**5r** (R=*p*-OH Ph)	49.7	67.8	87.3	56.7	57.7	54.6	36.2	46.2
**5s** (R=*m*-CF_3_ Ph)	44.3	54.8	63.5	66.7	70.9	44.7	49.7	55.7
**5t** (R=*α*-furyl)	63.2	72.2	48.6	66.7	42.1	84.1	41.6	62.9
**5u** (R=*α*-thienyl)	55.1	50.4	43.8	53.3	51.5	59.4	44.3	48.6
**5v** (R=*α*-pyridyl)	65.9	72.2	43.8	60.0	38.3	60.6	57.8	65.2
**5w** (R=n-butyl)	38.9	50.4	67.6	53.3	47.7	68.0	36.2	55.7
Chlorothalonil	100	73.3	75.0	73.9	73.1	96.1	90.4	91.3
Azoxystrobin	87.5	92.3	96.0	90.9	91.3	—	—	—

It was found that, at the concentration of 50 µg/mL, the target compounds exhibited the best antifungal activity against *P. piricola*, in which compounds **5b** (R= *o*-CH_3_ Ph), **5e** (R= *o*-OCH_3_ Ph), **5h** (R= *o*-F Ph), **5m** (R= *o*-Br Ph), **5o** (R= *m, m*-OCH_3_ Ph), and **5r** (R= *p*-OH Ph) had inhibition rates of 91.4, 83.3, 86.7, 83.8, 91.4 and 87.3%, respectively, much better than that of the positive control chlorothalonil. Moreover, compound **5a** (R= Ph) showed inhibition rate of 87.9% against *R. solani*, and compound **5b** (R= *o*-CH_3_ Ph) showed inhibition rate of 87.6% against *B. maydis*. It was also found that compound **5a** (R= Ph), **5b** (R= *o*-CH_3_ Ph), **5e** (R= *o*-OCH_3_ Ph), and **5m** (R= *o*-Br Ph) displayed inhibition rates of 81.9, 89.0, 81.9, and 84.3%, respectively, against *C. orbicalare*. On the whole, compound **5b** (R= *o*-CH_3_ Ph) is worthy of further study due to its better broad-spectrum antifungal activity against the eight tested plant pathogens. It was also found that the R groups displayed a noticeable influence on antifungal activity, a 3D-QSAR study was then carried out.

### 3D-QSAR Study

**FIGURE 3:**
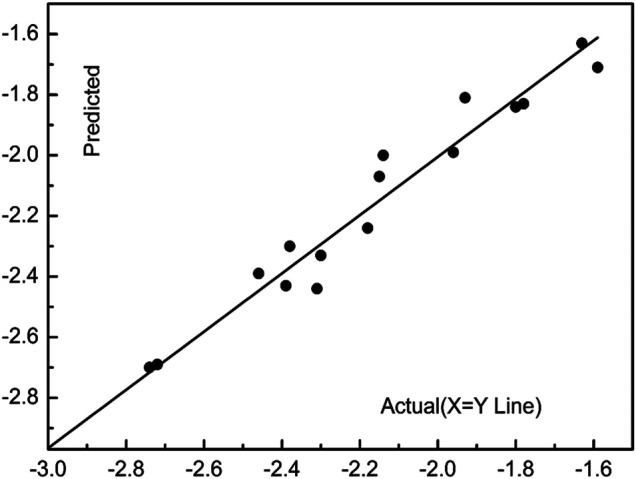
The experimental and predicted AF values.

The 3D-QSAR study was performed by CoMFA mothed to investigate the influence on the activity against *P. piricola* for the R groups. According to the report (Wang et al., 2016), the inhibition rates of the compounds **5a-5r** were converted to the available active factor (AF) values, which were listed in Table 3. The validity for the 3D-QSAR model was checked by the partial least squares (PLS) analysis including a correlation coefficient squared *r* ^*2*^ (close to 1), a cross-validated squared *q* ^*2*^ (>0.5), a standard deviation S (close to 0), a Fisher validation value F (>100), and an optimal number of component (ONC). Their values for the built 3D-QSAR model were shown in Table 2. The *r* ^*2*^ was 0.944, *q* ^*2*^ was 0.685, S was 0.099, F was 133.642, and ONC was 5, suggesting that the built 3D-QSAR model was valid. The experimental AF values, the predicted AF′ values, and their residue values were presented in Table 3, and the scatter diagram of AF vs. AF′ was shown in Figure 3. All data were concentrated near the X = Y line, also indicating that the 3D-QSAR model was reliable and had a good predictive ability.

**TABLE 2:** PLS analysis parameters for the built 3D-QSAR model.

*q* ^*2*^	*r* ^*2*^	*S*	*N*	*F*	Contribution (%)	
					Steric	Electrostatic
0.685	0.944	0.099	5	133.642	55.9	44.1

**TABLE 3:** The experimental and predicted AF values.

Compounds	R	WM	AF	AF′	Residue
**5a**	Ph	397.18	−2.46	−2.38	−0.08
**5b**	*o*-CH_3_ Ph	411.20	−1.59	−1.72	0.13
**5c**	*m*-CH_3_ Ph	411.20	−2.15	−2.08	−0.07
**5d**	*p*-CH_3_ Ph	411.20	−2.72	−2.68	−0.04
**5e**	*o*-OCH_3_ Ph	427.19	−1.93	−1.82	−0.11
**5f**	*m*-OCH_3_ Ph	427.19	−2.38	−2.31	−0.07
**5g**	*p*-OCH_3_ Ph	427.19	−2.74	−2.71	−0.03
**5h**	*o*-F Ph	415.17	−1.80	−1.83	0.03
**5i**	*m*-F Ph	415.17	−2.30	−2.34	0.04
**5k**	*o*-Cl Ph	431.14	−2.14	−2.01	−0.13
**5m**	*o*-Br Ph	477.09	−1.96	−1.98	0.02
**5n**	*m*-Br Ph	477.09	−2.39	−2.44	0.05
**5o**	*m*,*m*-OCH_3_ Ph	457.20	−1.63	−1.64	0.01
**5p**	*o*-NH_2_-m-CH_3_ Ph	426.21	−2.31	−2.43	0.12
**5q**	*m*-NH_2_-p-CH_3_ Ph	426.21	−2.18	−2.23	0.05
**5r**	*p*-OH Ph	413.18	−1.78	−1.84	0.06
5j*	*p*-F Ph	415.17	−2.56	−2.55	−0.01
5l*	*m*-Cl Ph	431.14	−2.40	−2.52	0.12
5s*	*m*-CF_3_ Ph	465.17	−2.43	−2.39	−0.04

AF = experimental value; AF′ = predicted value; *: test-set compound.

**FIGURE 4:**
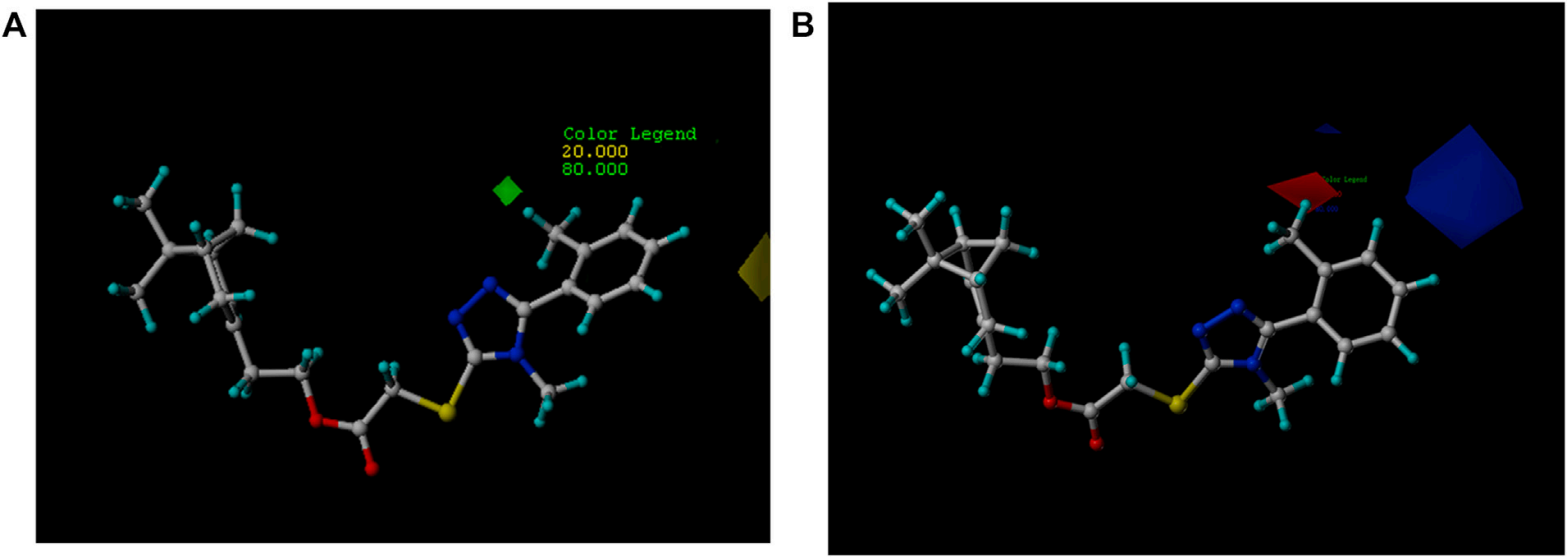
**(A)** Contours of steric contribution: green contour favors steric or bulky group, yellow contour denotes disfavored region. **(B)** Contours of electrostatic contribution: blue contour indicates electropositive charge, red contour electronegative charge.

**FIGURE 5:**
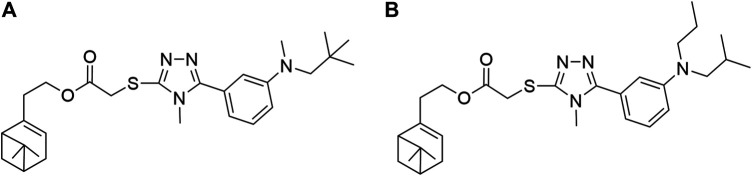
The proposed new molecules of 2-(6,6-dimethylbicyclo[3.1.1]hept-2-en-2yl)ethyl 2-((4-methyl-5-(3-(methyl(neopentyl)amino)phenyl)-4*H*-1,2,4-triazol-3-yl)thio)acetate **(A)**;2-(6,6-dimethylbicyclo[3,1,1]hept-2-en-2yl)ethyl 2-((5-(3-(isobutyl(propyl)amino)phenyl)-4-methyl-4*H*-1,2,4-triazol-3-yl)thio)acetate **(B)**.

The relative contributions of the steric and electrostatic fields of the 3D-QSAR model were 55.9 and 44.1%, respectively. In Figure 4A, the steric field contours were represented with two different colors: the green contour indicated that the R group embedding at 2-position of benzene ring was beneficial to increase the antifungal activity but yellow was the opposite. For instance, compounds **5b** (R= *o*-CH_3_ Ph) and **5e** (R= *o*-OCH_3_ Ph) possessed better antifungal activity against *P. piricola* than compounds **5d** (R= *p*-CH_3_ Ph) and **5g** (R= *p*-OCH_3_ Ph). In Figure 4B, the electrostatic contours were represented in two distinguishable colors: the blue enclosed volume represented that the R group with an electron-donating surface embedding in this area will favor the increase in activity, while red defines the opposite. The presence of electron-withdrawing groups such as fluorine or chlorine atom as well as bromine atom at 2-position of benzene ring was favorable for higher antifungal activity. For example, compounds **5h** (R= *o*-F Ph), **5k** (R= *o*-Cl Ph), and **5m** (R= *o*-Br Ph) showed a higher inhibitor rate against *P. piricola* than the unsubstituted compound **5a** (R= Ph). In contrast, the introduction of the electron-donating group at the 3-position of benzene ring played a crucial role in the antifungal activity. For instance, **5c** (R= *m*-CH_3_ Ph) and **5f** (R= *m*-OCH_3_ Ph) displayed better antifungal activity against *P. piricola*. The 3D-QSAR study of these title compounds can provide useful information for the further rational design of novel nopol derivatives. Herein, based on the results of 3D-QSAR analysis above, two novel unsynthesized compounds (Figure 5) were designed and the predicted ED values were calculated by the established CoMFA model. As a result, the designed compounds A (AF′ = −1.445) and B (AF′ = −1.589) showed potential excellent antifungal activities with inhibition rates of 94.68 and 92.93%, respectively, indicating that the antifungal activities of the proposed molecules were better than that of the compounds containing aromatic rings.

### Molecular Docking Analysis

**FIGURE 6:**
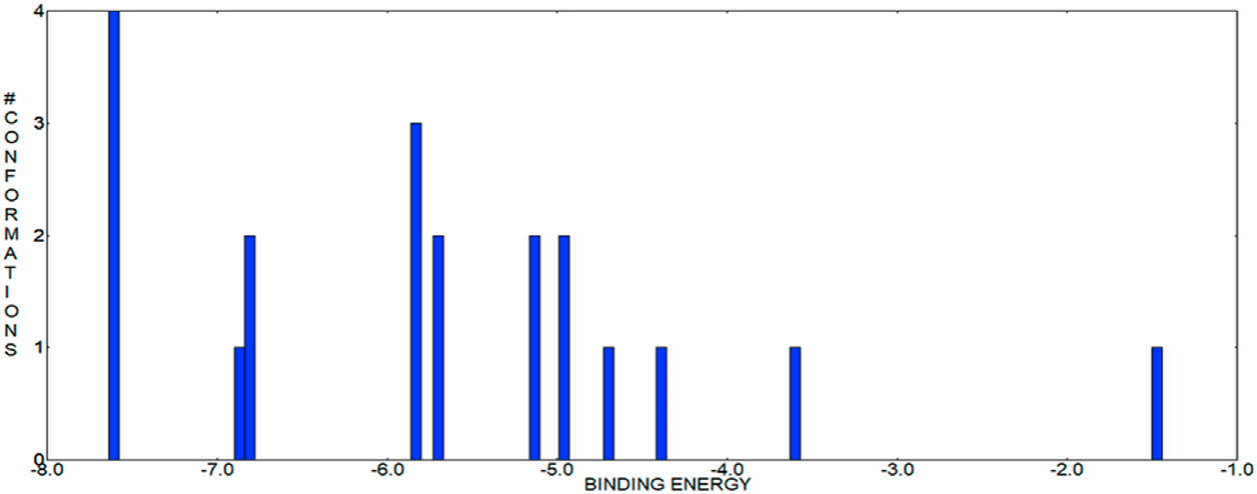
20 Docked conformations cluster for compound **5b**.

**FIGURE 7:**
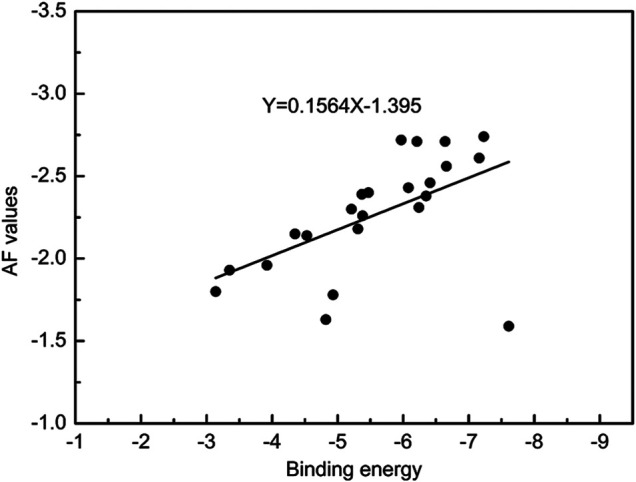
Monotonicity of binding energies versus AF values for the title compounds.

Using the cytochrome *bc*_1_ complex (PDB ID 1SQB) as the target protease, the molecular docking study for all the target compounds was performed to investigate the correlation between the binding energy and antifungal activity. There were 20 conformations docked for each target compound (Figure 6), and the lowest binding energy in the maximum cluster for the docked conformations was chosen as the representative binding energy for the corresponding compound. The scatter plot of AF values versus binding energies for the title compounds was shown in Figure 7. All of the data were concentrated near the line Y = 0.1564X-1.395, illustrating that there was a clear positive monotonic association between AF values and binding energies. In addition, Spearman’s rank correlation coefficient analytical approach was carried out using IBM SPSS STATITICS 22 software to investigate the correlation between AF values and binding energies. The result were listed in Table 4 and Table 5. It was found that the correlation was significant at 0.001 (at 0.01 level). The Spearman correlation coefficient was 0.626, indicating that there was a significant positive correlation, namely, the activity gradually increased with the increase in binding energies. It suggested that the cytochrome *bc*_1_ complex was a potential target protease.

**TABLE 4:** Binding energies and AF values of the target compounds **5a-5w**.

Compounds	R	Binding free energies (kcal/mol)	AF
5a	Ph	−6.41	−2.46
**5b**	*o*-CH_3_ Ph	−7.61	−1.59
**5c**	*m*-CH_3_ Ph	−4.35	−2.15
**5d**	*p*-CH_3_ Ph	−5.97	−2.72
**5e**	*o*-OCH_3_ Ph	−3.35	−1.93
**5f**	*m*-OCH_3_ Ph	−6.35	−2.38
**5g**	*p*-OCH_3_ Ph	−7.23	−2.74
**5h**	*o*-F Ph	−3.14	−1.80
**5i**	*m*-F Ph	−5.21	−2.30
**5j**	*p*-F Ph	−6.66	−2.56
**5k**	*o*-Cl Ph	−4.53	−2.14
**5l**	*m*-Cl Ph	−5.47	−2.40
**5m**	*o*-Br Ph	−3.92	−1.96
**5n**	*m*-Br Ph	−5.37	−2.39
**5o**	*m,m*-OCH_3_ Ph	−4.82	−1.63
**5p**	*o-*NH_2_ *-m*-CH_3_ Ph	−6.24	−2.31
**5q**	*m-*NH_2_ *-p*-CH_3_ Ph	−5.31	−2.18
**5r**	*p*-OH Ph	−4.93	−1.78
**5s**	*m*-CF_3_ Ph	−6.08	−2.43
**5t**	*α*-furyl	−7.16	−2.61
**5u**	*α*-thienyl	−6.64	−2.71
**5v**	*α*-pyridyl	−6.21	−2.71
**5w**	n-butyl	−5.38	−2.26
Azoxystrobin	—	−6.67	−1.23

**TABLE 5:** Spearman rank correlation coefficient of AF values vs. binding energies for the target compounds.

		Binding energies	AF values
Binding energies	Correlation coefficient	1.000	0.626
	Sig. (1-tailed)	—	0.001
AF values	Correlation coefficient	0.626	1.000
		Sig. (1-tailed)	0.001

**FIGURE 8:**
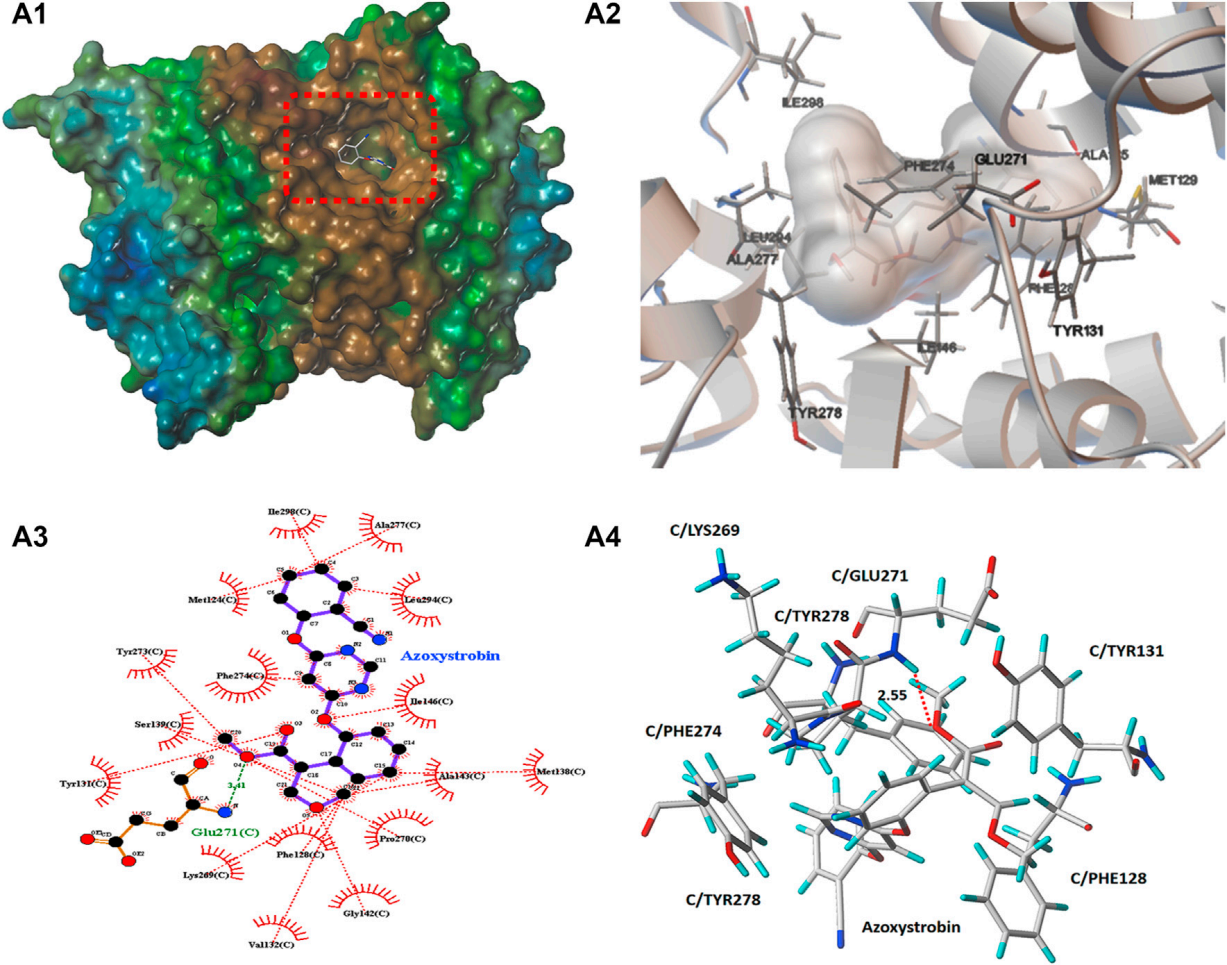
**(A1)** The molecular docking of compound azoxystrobin with cytochrome *bc*_1_ complex. **(A2)** The binding conformation of compound azoxystrobin in the active site of cytochrome *bc*_1_ complex. **(A3)** The 2D interactions mao of compound azoxystrobin with cytochrome *bc*_1_ complex. **(A4)** The 3D interactions map of compound azoxystrobin with cytochrome *bc*_1_ complex.

**FIGURE 9:**
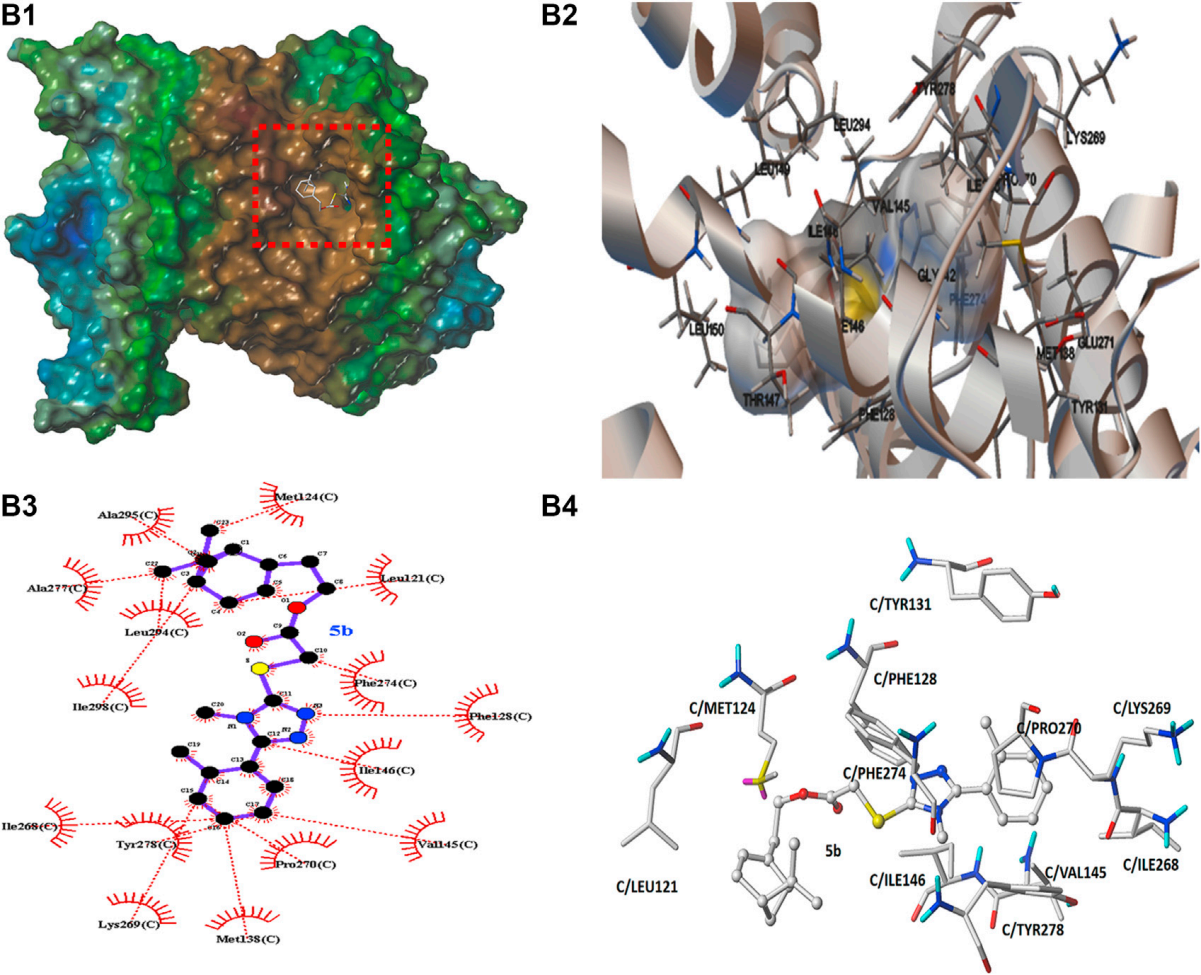
**(B1)** The molecular docking of compound **5b** with cytochrome *bc*_1_ complex. **(B2)** The binding conformation of copound **5b** in the active site of cytochrome *bc*_1_ complex. **(B3)** The 2D interactions map of compound **5b** with cytochrome *bc*_1_ complex. **(B4)** The 3D interactions map of compound **5b** with cytochrome *bc*_1_ complex.

In order to preliminarily explore the possible binding mode of compound **5b** possessing the best antifungal activity with the typical cytochrome *bc*_1_ complex inhibitor Azoxystrobin, molecular docking analysis was carried out on Sybyl X 2.1.1 program and AutoDock 4.2.6 software. The result was showed in Figures 8, 9. In Figures 8A1, 9B1, it can be observed that the compound **5b** was well embedded into the active domain where the ligand azoxystrobin was found. The binding conformation of compound azoxystrobin and **5b** in the active site of cytochrome *bc*_1_ complex, as well as the 2D interactions map of compound azoxystrobin and **5b** with residues were presented in Figures 8A2,B2,A3,B3. The 3D interactions map of compound azoxystrobin with residues were showed in Figure 8A4, and compound **5b** with residues in Figure 9B4. It was found that compound **5b** was buried into the binding pocket consisting of the residues LEU121, MET124, PHE274, PRO270, PHE128, TYR131, LYS269, ILE146, VAL145, etc. Meanwhile, two π-π stacking interactions (distance = 2.12 and 2.90 Å) were observed between compound **5b** and the residues PHE128 and PHE274, respectively. In addition, two π-π stacking interactions were observed between azoxystrobin and the residues PHE274 and TYR278, respectively. A H-bond was formed by the oxygen atom of methoxyacrylate moiety and the residue GLU271 in cytochrome *bc*_1_ complex binding site (distance = 2.55 Å), which was essential to stabilize the binding between cytochrome *bc*_1_ complex inhibitors and cytochrome *bc*_1_ complex. Based on these results, the binding domain and the binding mode of compound **5b** were both similar to that of azoxystrobin, suggesting that they shared a similar action mode.

## Conclusion

Using molecular docking-based virtual screening in the assumption of cytochrome *bc*_1_ complex as the target enzyme, and natural preponderant resource *β*-pinene as starting material, twenty-three novel nopol-based 1,2,4-triazole-thioether compounds were designed and synthesized. Their structures were confirmed by FT-IR, NMR, ESI-MS, and elemental analysis. The *in vitro* antifungal activity of the target compounds **5a-5w** was preliminarily evaluated against eight plant pathogens at the concentration of 50 µg/mL. The bioassay results indicated that some of the target compounds showed excellent inhibitory activity against the tested fungi, especially against *P. piricola*. Compound **5b** (R= *o*-CH_3_ Ph) exhibited better broad-spectrum antifungal activity against the tested fungi. There was a significant positive Spearman’s rank correlation between the antifungal activity and the docking-based binding free energy. Molecular docking study revealed that there was hydrophobic interactions between the target compounds and the key favorable residues of cytochrome *bc*_1_ complex. In addition, a reasonable and effective 3D-QSAR model (*r* ^*2*^ = 0.944, *q* ^*2*^ = 0.685) has been established for the further development of novel and promising antifungal compounds.

## Data Availability

The crystallographic data of the target protein cytochrome *bc*_1_ complex (PDB ID 1SQB, 2.69 Å in resolution) used in the molecular docking study of our article was downloaded from the RCSB PDB web (https://www.rcsb.org/).

## References

[B1] Abdel-Aziz M. Beshr E. A. Abdel-Rahman I. M. Ozadali K. Tan O. U. Aly O. M. (2014). 1-(4-Methoxyphenyl)-5-(3,4,5-trimethoxyphenyl)-1H-1,2,4-triazole-3-carboxamides: Synthesis, Molecular Modeling, Evaluation of Their Anti-inflammatory Activity and Ulcerogenicity. Eur. J. Med. Chem. 77, 155–165. 10.1016/j.ejmech.2014.03.001 24631895

[B2] Chen C. Wu Q. Y. Shan L. Y. Zhang B. Verpoort F. Yang G. F. (2016). Discovery of Cytochrome *Bc* _1_ Complex Inhibitors Inspired by the Natural Product Karrikinolide. RSC Adv. 6, 97580–97586. 10.1039/c6ra19424a

[B3] Chen J. Z. Xiao Z. Q. Xu L. F. Wang Z. D. (2017). The Synthesis and Antifungal of Hydronopyl Alkylamine and its Acetylation Derivative. Chem. Res. Appl. 29, 1728–1732. 10.14233/ajchem.2017.20224

[B4] Chen M. Duan W. G. Lin G. S. Fan Z. T. Wang X. (2021). Synthesis, Antifungal Activity, and 3D-QSAR Study of Novel Nopol-Derived 1,3,4-Thiadiazole-Thiourea Compounds. Molecules 26, 1708–1723. 10.3390/molecules26061708 33803890 PMC8003325

[B5] Chen Y. Li P. Su S. Chen M. He J. Liu L. (2019). Synthesis and Antibacterial and Antiviral Activities of Myricetin Derivatives Containing a 1,2,4-triazole Schiff Base. RSC Adv. 9, 23045–23052. 10.1039/c9ra05139b PMC9067368 35514467

[B6] Cheng H. Fu Y. Chang Q. Zhang N. Bu M. Niu Y. (2018). Synthesis, Biochemical Evaluation and Computational Simulations of New Cytochrome *Bc* _1_ Complex Inhibitors Based on N-(4-aryloxyphenyl) Phthalimides. Chin. Chem. LettersChem. Lett. 29, 1897–1900. 10.1016/j.cclet.2018.10.008

[B7] Du S. J. Qin Z. H. Xu L. (2020). Discovery of Novel Complex III Inhibitor by Docking-Based Virtual Screening. Chin. J. Pestic. Sci. 22, 942–950.

[B8] El-Sherief H. A. M. Youssif B. G. M. Abbas Bukhari S. N. Abdelazeem A. H. Abdel-Aziz M. Abdel-Rahman H. M. (2018). Synthesis, Anticancer Activity and Molecular Modeling Studies of 1,2,4-triazole Derivatives as EGFR Inhibitors. Eur. J. Med. Chem. 156, 774–789. 10.1016/j.ejmech.2018.07.024 30055463

[B9] Feng X. Z. Xiao Z. Q. Lu P. Y. Fan G. R. Wang Z. D. (2019). Synthesis and Antibacterial Activity of Hydronopol Dimethyl Alkyl Ammonium Halide. Chem. Ind. For. Prod. 39, 35–40.

[B10] Frac (2021). FRAC Classification of Fungicides. Availableat: http://www.frac.info/ (accessed Aug 12, 2021).

[B11] Gao F. Wang T. Xiao J. Huang G. (2019). Antibacterial Activity Study of 1,2,4-triazole Derivatives. Eur. J. Med. Chem. 173, 274–281. 10.1016/j.ejmech.2019.04.043 31009913

[B12] García D. Bustamante F. Villa A. L. Lapuerta M. Alarcón E. (2020). Oxyfunctionalization of Turpentine for Fuel Applications. Energy Fuels 34, 579–586. 10.1021/acs.energyfuels.9b03742

[B13] Han Z. J. Wang Z. D. Jiang Z. K. Jin X. Y. Qian W. H. Chen C. (2007). Antifeedant Activity of Terpene Compounds against Larvae of the Diamondback Moth, Plutella Xylostella. Bull. Entomol. 44, 863–867.

[B14] Han Z. J. Wang Z. D. Jiang Z. K. Zheng W. Q. Qian W. H. Chen J. Z. (2008). The Synthesis of Bridge-Ring Terpenoids and Their Repellent Activities against House Antsn (Monomorium Pharaonis). Acta Agric. Univ. Jiangxiensis. 30, 586–591.

[B15] Hao G. F. Wang F. Li H. Zhu X. L. Yang W. C. Huang L. S. (2012). Computational Discovery of Picomolar Qo Site Inhibitors of Cytochrome *Bc* _1_ Complex. J. Am. Chem. Soc. 134, 11168–11176. 10.1021/ja3001908 22690928

[B16] Hao G. F. Yang S. G. Huang W. Wang L. Shen Y. Q. Tu W. L. (2015). Rational Design of Highly Potent and Slow-Binding Cytochrome *Bc* _1_ Inhibitor as Fungicide by Computational Substitution Optimization. Sci. Rep. 5, 13471–13480. 10.1038/srep13471

[B17] He L. L. Tian Q. Yang Q. Zhu X. M. Yang J. S. (2008). Study on Synthesis of Pinaverium Bramide. Chem. Res. Appl. 20, 600–602.

[B18] He Y. Wang X. Duan W. G. Lin G. S. Cen B. Lei F. H. (2021). Synthesis and Antifungal Activity of Citral-Based 1,2,3-triazole Compounds. Fine Chem. 38, 640–648.

[B19] Hou Y. L. Zhu L. Y. Li Z. W. Shen Q. Xu Q. L. Li W. (2019). Design, Synthesis and Biological Evaluation of Novel 7-Amino-[1,2,4]triazolo[4,3-F]pteridinone, and 7-Aminotetrazolo[1,5-F]pteridinone Derivative as Potent Antitumor Agents. Eur. J. Med. Chem. 163, 690–709. 10.1016/j.ejmech.2018.12.009 30572179

[B20] Huang M. Duan W.-G. Lin G.-S. Li K. Hu Q. (2017). Synthesis and Antifungal Activity of Novel 3-Caren-5-One Oxime Esters. Molecules 22, 1538–1552. 10.3390/molecules22091538 PMC6151701 28895932

[B21] Jadhav S. V. Jinka K. M. Bajaj H. C. (2010). Synthesis of Nopol via Prins Condensation of *β*-pinene and Paraformaldehyde Catalyzed by Sulfated Zirconia. Appl. Catal. A: Gen. 390, 158–165. 10.1016/j.apcata.2010.10.005

[B22] Jiao J. Chen M. Sun S. X. Si W. J. Wang X. B. Ding W. J. (2021). Synthesis, Bioactivity Evaluation, 3D‐QSAR , and Molecular Docking of Novel Pyrazole‐4‐carbohydrazides as Potential Fungicides Targeting Succinate Dehydrogenase. Chin. J. Chem. 39, 323–329. 10.1002/cjoc.202000438

[B23] Jin L. L. Xiao Z. Q. Fan G. R. Chen J. Z. Wang P. Wang Z. D. (2017). Synthesis and Antifungal Activity of Series of N-Hydronopol Pyridine Ammonium Halide. Chem. Ind. For. Prod. 37, 122–128.

[B24] Kang G. Q. Duan W. G. Lin G. S. Yu Y. P. Wang X. Y. Lu S. Z. (2019). Synthesis of Bioactive Compounds from 3-carene (II): Synthesis, Antifungal Activity and 3D-QSAR Study of (Z)- and (E)-3-caren-5-one Oxime Sulfonates. Molecules 24, 477–490. 10.3390/molecules24030477 PMC6384770 30699975

[B25] Karczmarzyk Z. Swatko-Ossor M. Wysocki W. Drozd M. Ginalska G. Pachuta-Stec A. (2020). New Application of 1,2,4-triazole Derivatives as Antitubercular Agents. Structure, *In Vitro* Screening and Docking Studies. Molecules 25, 6033–6050. 10.3390/molecules25246033 PMC7767103 33352814

[B26] Li B. L. Li B. Zhang R. L. Zhao J. J. Wang X. F. Liu Y. M. (2016). Synthesis and Antiproliferative Evaluation of Novel 1,2,4-triazole Derivatives Incorporating Benzisoselenazolone Scaffold. Bioorg. Med. Chem. Lett. 26, 1279–1281. 10.1016/j.bmcl.2016.01.017 26786698

[B27] Liao L. P. Jiang C. B. Chen J. W. Shi J. G. Li X. H. Wang Y. (2020). Synthesis and Biological Evaluation of 1,2,4-triazole Derivatives as Potential Neuroprotectant against Ischemic Brain Injury. Eur. J. Med. Chem. 190, 112114–112129. 10.1016/j.ejmech.2020.112114 32061962

[B28] Lin G. S. Duan W. G. Yang L. X. Huang M. Lei F. H. (2017). Synthesis and Antifungal Activity of Novel Myrtenal-Based 4-Methyl-1,2,4-Triazole-Thioethers. Molecules 22, 193–202. 10.3390/molecules22020193 PMC6155697 28125042

[B29] Lin G. S. Bai X. Duan W. G. Cen B. Huang M. Lu S. Z. (2019). High Value-Added Application of Sustainable Natural Forest Product α-Pinene: Synthesis of Myrtenal Oxime Esters as Potential KARI Inhibitors. ACS Sustainable Chem. Eng. 7, 7862–7868. 10.1021/acssuschemeng.9b00254

[B30] Lin G. S. Chen Z. C. Duan W. G. Wang X. Y. Lei F. H. (2018). Synthesis and Biological Activity of Novel Myrtenal-Derived 2-Acyl-1,2,4-Triazole-3-Thione Compounds. Chin. J. Org. Chem. 38, 2085–2092. 10.6023/cjoc201801043

[B31] Liu C. J. Shi T. H. Li Y. P. (2007). Synthesis of Bisoxadiazolyl Substituted Sulfides and Sulfoxides Containing 2 Trifluoromethylbenzimidazol-1-Yl Group. Chin. J. Org. Chem. 27, 985–988. 10.1016/j.bmcl.2007.03.088

[B32] Liu X. H. Pan L. Weng J. Q. Tan C. X. Li Y. H. Wang B. L. (2012). Synthesis, Structure, and Biological Activity of Novel (Oxdi/tri)azoles Derivatives Containing 1,2,3-thiadiazole or Methyl Moiety. Mol. Divers. 16, 251–260. 10.1007/s11030-011-9352-z 22249419

[B33] Liu X. L. Hu T. T. Lin G. S. Wang X. Zhu Y. Liang R. Z. (2020). The Synthesis of a DHAD/ZnAlTi-LDH Composite with Advanced UV Blocking and Antibacterial Activity for Skin protection. RSC Adv. 10, 9786–9790. 10.1039/d0ra00572j PMC9050216 35498563

[B34] Majouli K. Mahjoub M. A. Rahim F. Hamdi A. Wadood A. Besbes Hlila M. (2017). Biological Properties of Hertia Cheirifolia L. Flower Extracts and Effect of the Nopol on α-glucosidase. Int. J. Biol. Macromolecules 95, 757–761. 10.1016/j.ijbiomac.2016.12.008 27939269

[B35] Mustafa M. Anwar S. Elgamal F. Ahmed E. R. Aly O. M. (2019). Potent Combretastatin A-4 Analogs Containing 1,2,4-triazole: Synthesis, Antiproliferative, Anti-tubulin Activity, and Docking Study. Eur. J. Med. Chem. 183, 111697–111708. 10.1016/j.ejmech.2019.111697 31536891

[B36] Nie G. Zou J.-J. Feng R. Zhang X. Wang L. (2014). HPW/MCM-41 Catalyzed Isomerization and Dimerization of Pure Pinene and Crude Turpentine. Catal. Today 234, 271–277. 10.1016/j.cattod.2013.12.003

[B37] Peng Z. Y. Wang G. C. Zeng Q. H. Li Y. F. Wu Y. Liu H. Q. (2021). Synthesis, Antioxidant and Anti-tyrosinase Activity of 1,2,4-triazole Hydrazones as Antibrowning Agents. Food Chem. 341, 128265–128273. 10.1016/j.foodchem.2020.128265 33031957

[B38] Qi L. Li M.-C. Bai J.-C. Ren Y.-H. Ma H.-X. (2021). *In Vitro* antifungal Activities, Molecular Docking, and DFT Studies of 4-Amine-3-Hydrazino-5-Mercapto-1,2,4-Triazole Derivatives. Bioorg. Med. Chem. Lett. 40, 127902–127907. 10.1016/j.bmcl.2021.127902 33684439

[B39] Shi J. Ding M. H. Luo N. Wan S. R. Li P. J. Li J. H. (2020). Design, Synthesis, crystal Structure, and Antimicrobial Evaluation of 6-Fluoroquinazolinylpiperidinyl-Containing 1,2,4-triazole Mannich Base Derivatives against Phytopathogenic Bacteria and Fungi. J. Agric. Food Chem. 68, 9613–9623. 10.1021/acs.jafc.0c01365 32786823

[B40] Su N. N. Li Y. Yu S. J. Zhang X. Liu X. H. Zhao W. G. (2013). Microwave-assisted Synthesis of Some Novel 1,2,3-triazoles by Click Chemistry, and Their Biological Activity. Res. Chem. Intermed. 39, 759–766. 10.1007/s11164-012-0595-9

[B41] Vrbková E. Šteflová B. Vyskočilová E. Červený L. (2020). Heterogeneous Mo/W/Zn-SiO2 Based Catalysts in Nopol (2-(6,6-dimethyl-2-bicyclo[3.1.1]hept-2-Enyl)ethanol) Synthesis. Reac Kinet Mech. Cat 131, 213–232. 10.1007/s11144-020-01858-w

[B42] Wang B. L. Shi Y. X. Zhang S. J. Ma Y. Wang H. X. Zhang L. Y. (2016). Syntheses, Biological Activities and SAR Studies of Novel Carboxamide Compounds Containing Piperazine and Arylsulfonyl Moieties. Eur. J. Med. Chem. 117, 167–178. 10.1016/j.ejmech.2016.04.005 27092414

[B43] Wang F. Li H. Wang L. Yang W.-C. Wu J.-W. Yang G.-F. (2011). Design, Syntheses, and Kinetic Evaluation of 3-(phenylamino)oxazolidine-2,4-Diones as Potent Cytochrome *Bc* _1_ Complex Inhibitors. Bioorg. Med. Chem. 19, 4608–4615. 10.1016/j.bmc.2011.06.008 21719298

[B44] Wang H. Gao X. Zhang X. Jin H. Tao K. Hou T. (2017). Design, Synthesis and Antifungal Activity of Novel Fenfuram-Diarylamine Hybrids. Bioorg. Med. Chem. Lett. 27, 90–93. 10.1016/j.bmcl.2016.11.026 27884696

[B45] Wang L. L. Zhao S. S. Kong X. T. Cao L. L. Tian S. Ye Y. H. (2018a). Design, Synthesis and Fungicidal Evaluation of Novel Pyraclostrobin Analogues. Bioorg. Med. Chem. 26, 875–883. 10.1016/j.bmc.2018.01.004 29395803

[B46] Wang X. Pang F. H. Huang L. Yang X. P. Ma X. L. Jiang C. N. (2018b). Synthesis and Biological Evaluation of Novel Dehydroabietic Acid-Oxazolidinone Hybrids for Antitumor Properties. Int. J. Mol. Sci. 19, 3116–3133. 10.3390/ijms19103116 PMC6213879 30314336

[B47] Xu B. Zhai Q. H. Li Z. X. Zheng G. Y. (1992). Studies on the Yield and Chemical Compositions of Oleoresin from Pinus Elliottii Engelm. In Different Tappine Seasons. Chem. Ind. For. Prod. 12, 75–82.

[B48] Xu J. M. Cao Y. B. Zhang J. Yu S. C. Zou Y. Chai X. Y. (2011). Design, Synthesis and Antifungal Activities of Novel 1,2,4-triazole Derivatives. Eur. J. Med. Chem. 46, 3142–3148. 10.1016/j.ejmech.2011.02.042 21420761

[B49] Yadav M. K. Jasra R. V. (2006). Synthesis of Nopol from **β**-pinene Using ZnCl2 Impregnated Indian Montmorillonite. Catal. Commun. 7, 889–895. 10.1016/j.catcom.2006.04.002

[B50] Yi F. P. Ke M. Wang L. S. (2000). Study on Optimum Condition of Reaction between *β*-pinene and Paraformaldehyde Catalyzed by ZnCl_2_ . Chem. Ind. For. Prod. 20, 55–58.

[B51] Zhao S. Y. Lin G. S. Duan W. G. Zhang Q. A. Huang Y. L. Lei F. H. (2021). Design, Synthesis, and Antifungal Activity of Novel Longifolene-Derived Diacylhydrazine Compounds. ACS Omega 6, 9104–9111. 10.1021/acsomega.1c00217 33842780 PMC8028131

[B52] Zhu X. L. Zhang R. Wu Q. Y. Song Y. J. Wang Y. X. Yang J. F. (2019). Natural Product Neopeltolide as a Cytochrome *Bc* _1_ Complex Inhibitor: Mechanism of Action and Structural Modification. J. Agric. Food Chem. 67, 2774–2781. 10.1021/acs.jafc.8b06195 30794394

[B53] Zhu X. P. Lin G. S. Duan W. G. Li Q. M. Li F. Y. Lu S. Z. (2020). Synthesis and Antiproliferative Evaluation of Novel Longifolene-Derived Tetralone Derivatives Bearing 1,2,4-triazole Moiety. Molecules 25, 986–997. 10.3390/molecules25040986 PMC7070458 32098438

