# Supplementary material for: Synthesis, 3D-QSAR and Molecular Docking Study of Nopol-Based 1,2,4-Triazole-Thioether Compounds as Potential Antifungal Agents
Source: Front Chem. 2021 Oct 25;9:757584. doi: 10.3389/fchem.2021.757584 (PMC8576812; doi:10.3389/fchem.2021.757584)
Supplement: Supplementary file 1 [file DataSheet1.docx]

Supplementary Material

**NMR-Spectra**


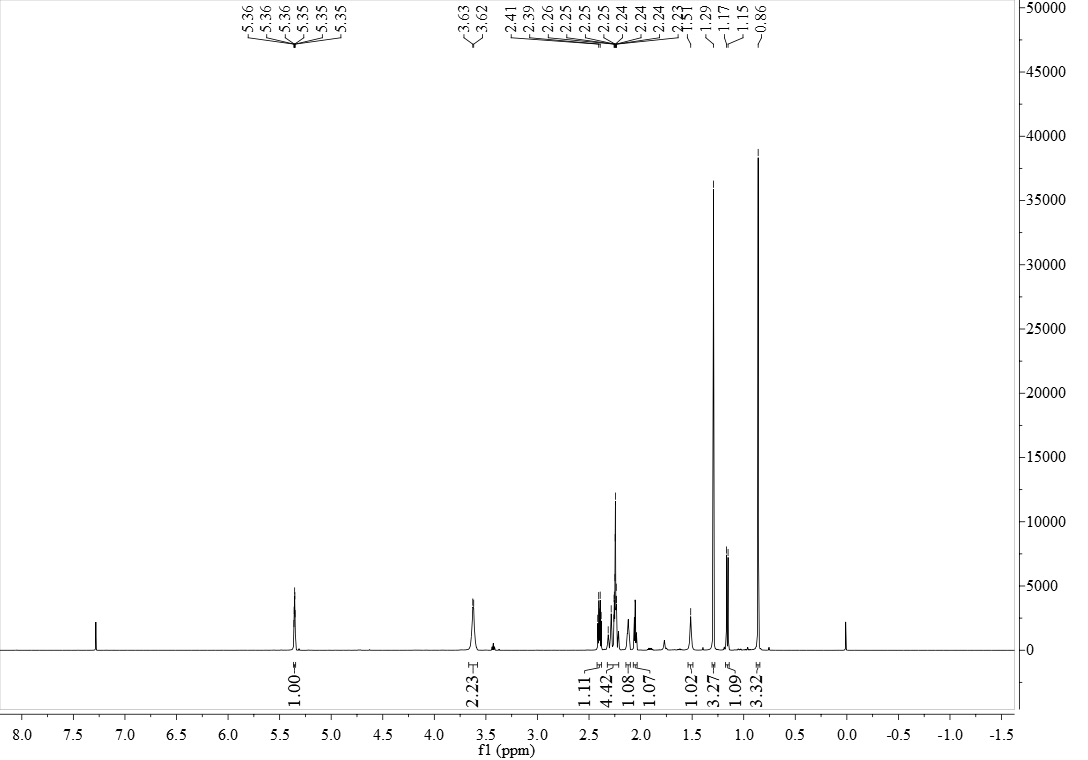


**FigureS1.** ^1^H NMR (600 MHz, CDCl_3_) of compound **2**


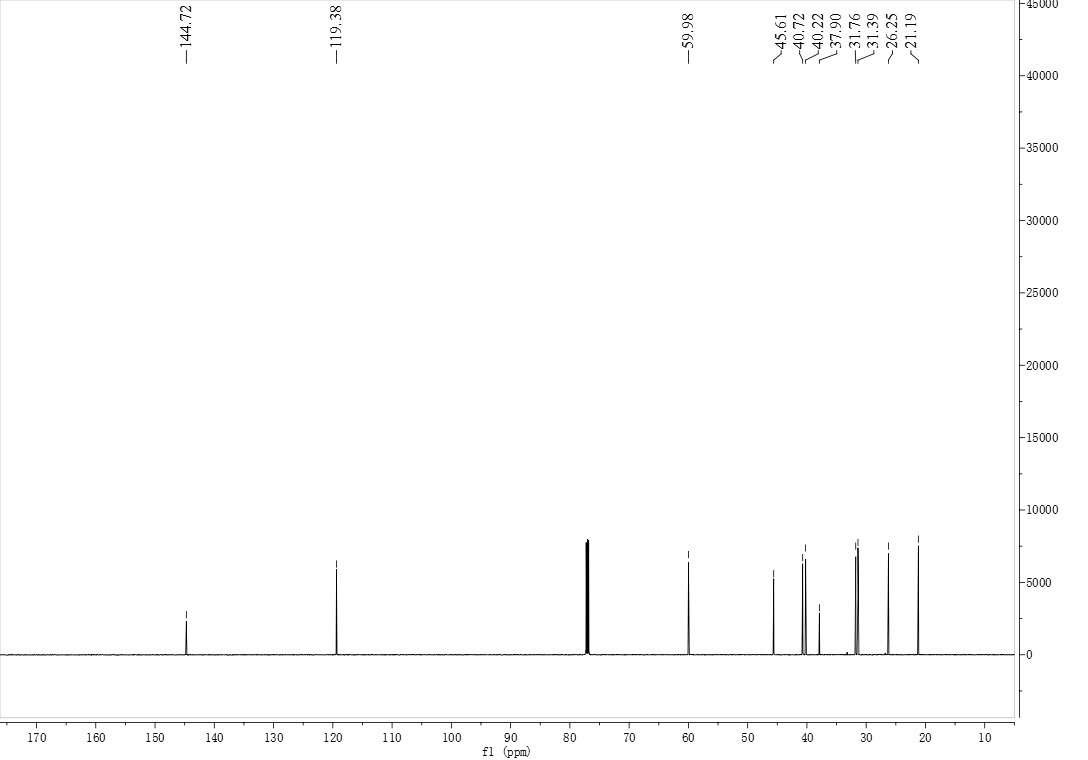


**FigureS2.** ^13^C NMR (150 MHz, CDCl_3_) of compound **2**


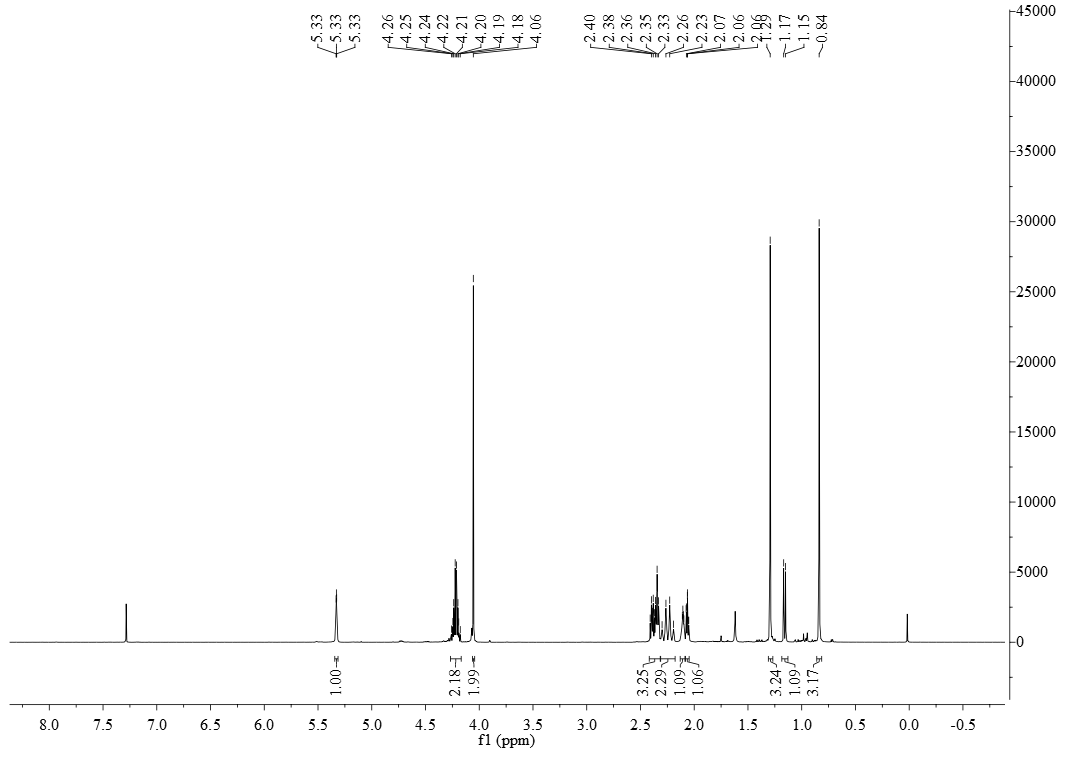


**FigureS3.** ^1^H NMR (600 MHz, CDCl_3_) of compound **3**


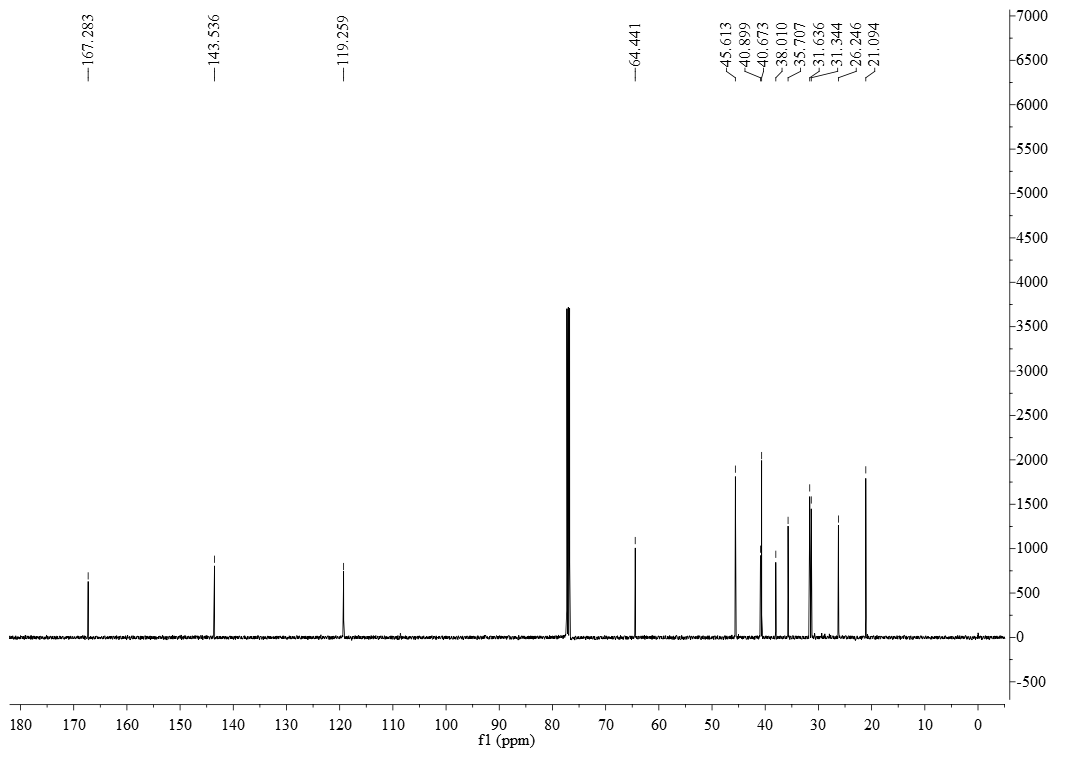


**FigureS4.** ^13^C NMR (150 MHz, CDCl_3_) of compound **3**


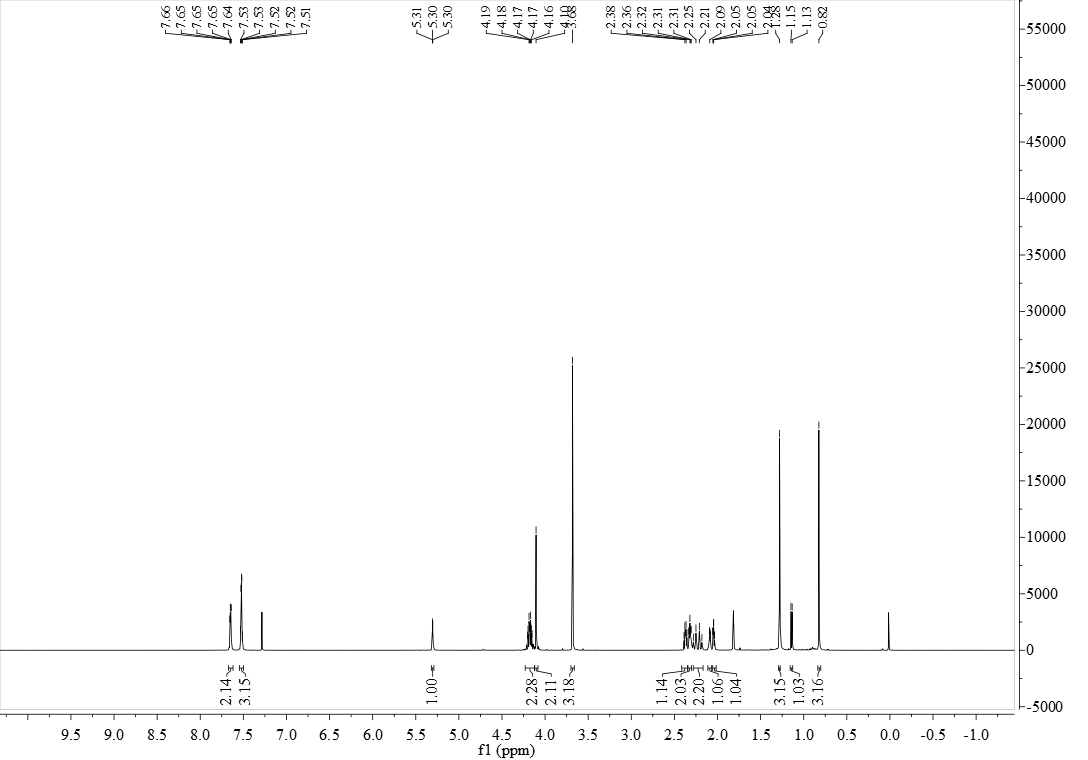


**FigureS5.** ^1^H NMR (600 MHz, CDCl_3_) of compound **5a**


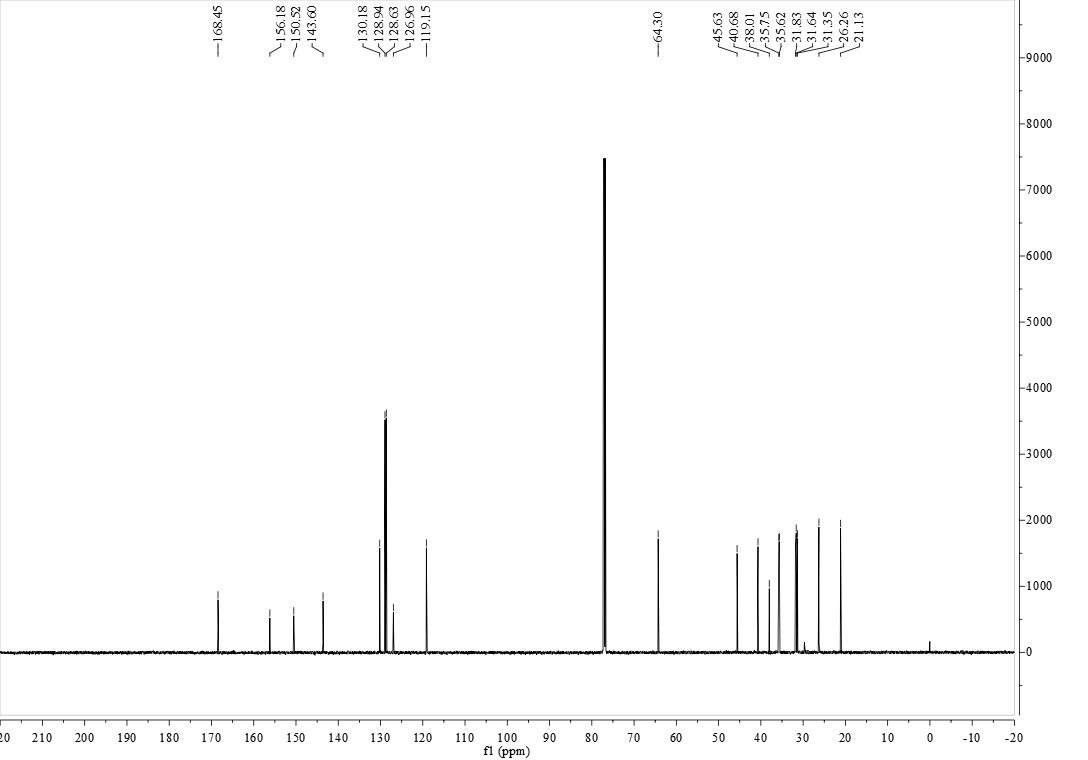


**FigureS6.** ^13^C NMR (150 MHz, CDCl_3_) of compound **5a**


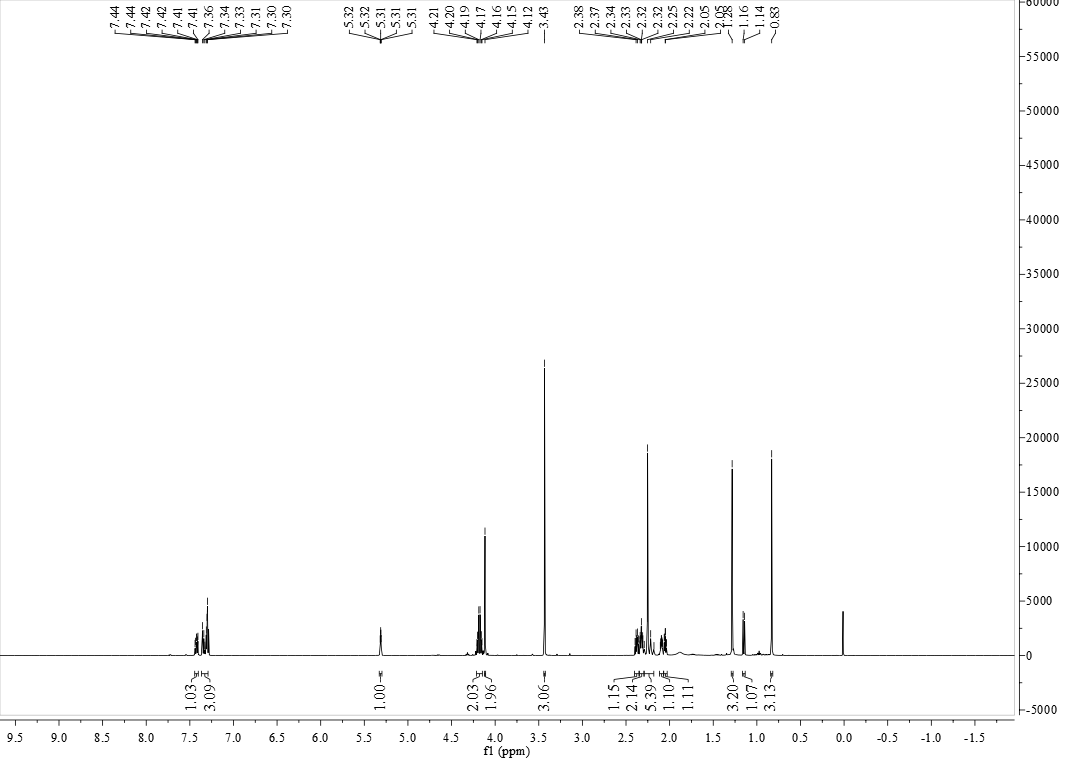


**FigureS7.** ^1^H NMR (600 MHz, CDCl_3_) of compound **5b**


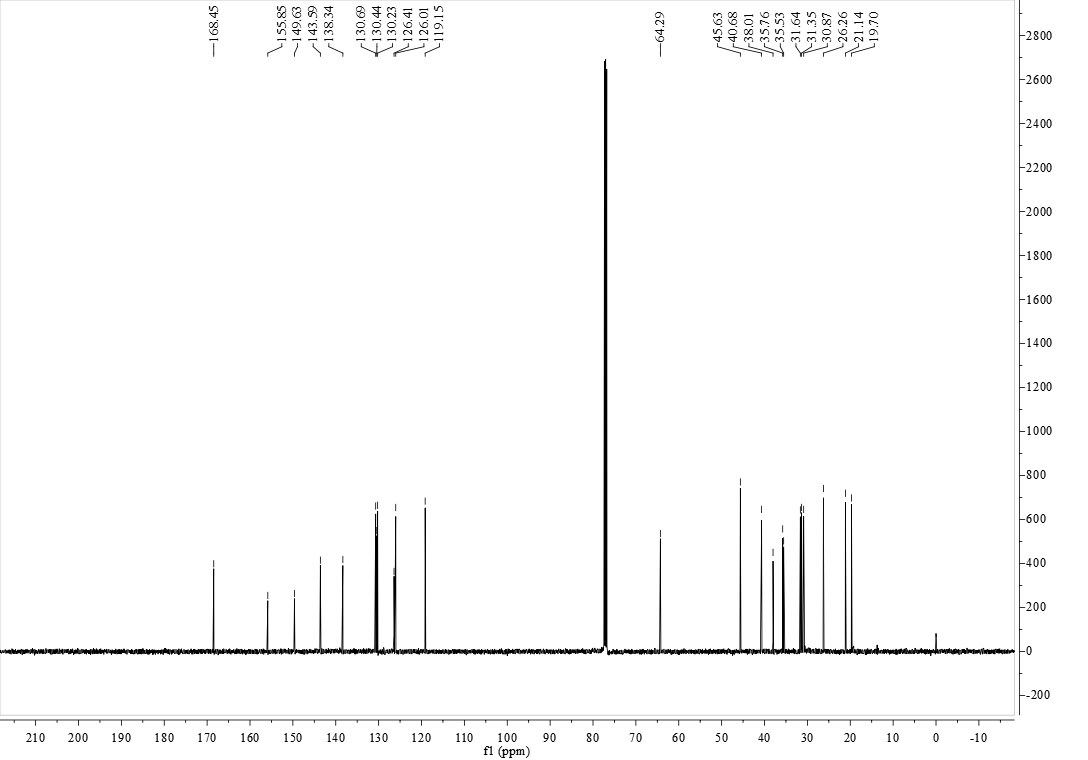


**FigureS8.** ^13^C NMR (150 MHz, CDCl_3_) of compound **5b**


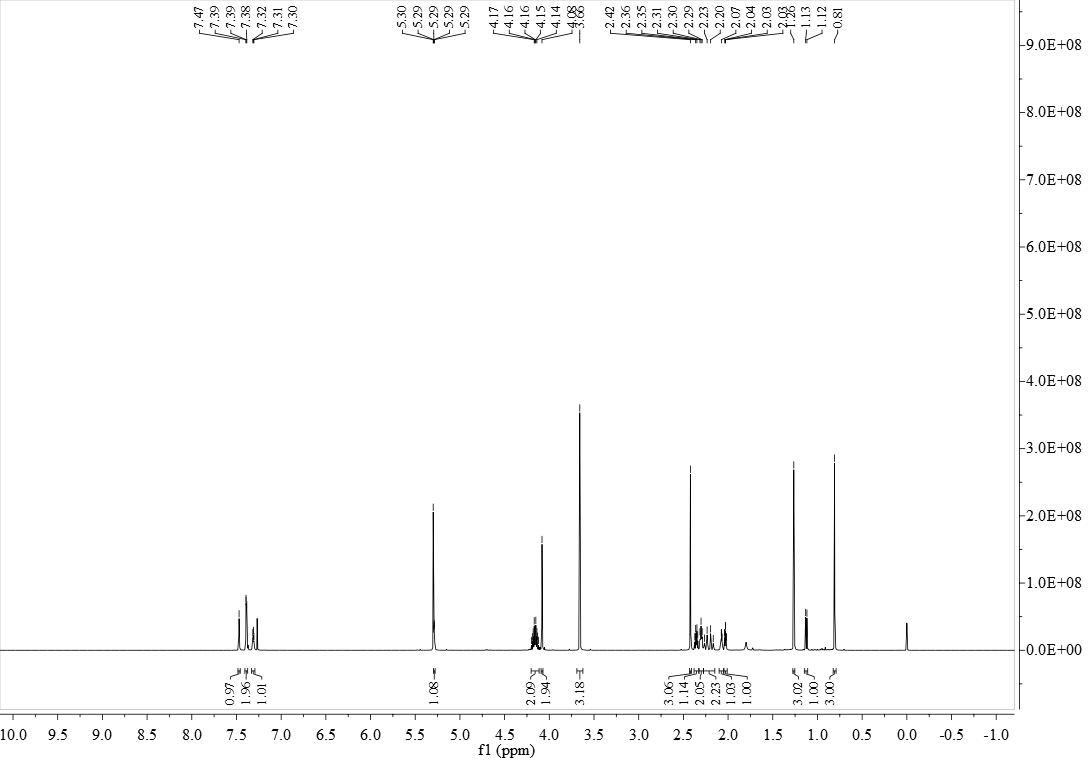


**FigureS9.** ^1^H NMR (600 MHz, CDCl_3_) of compound **5c**


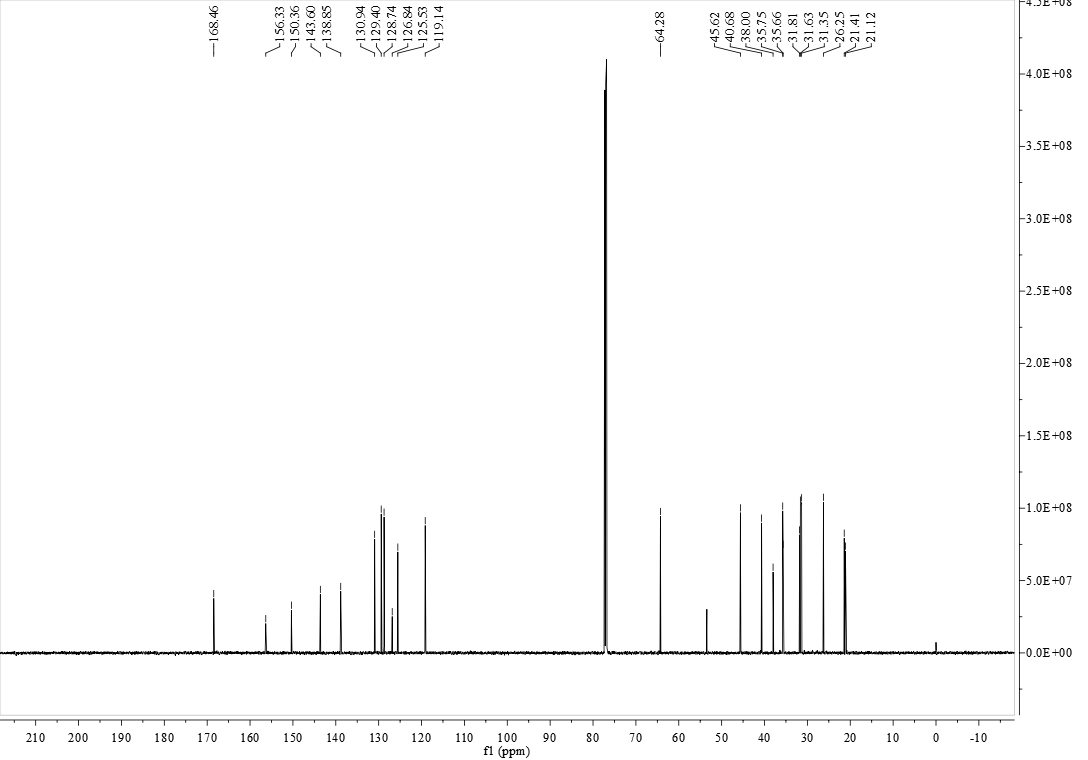


**FigureS10.** ^13^C NMR (150 MHz, CDCl_3_) of compound **5c**


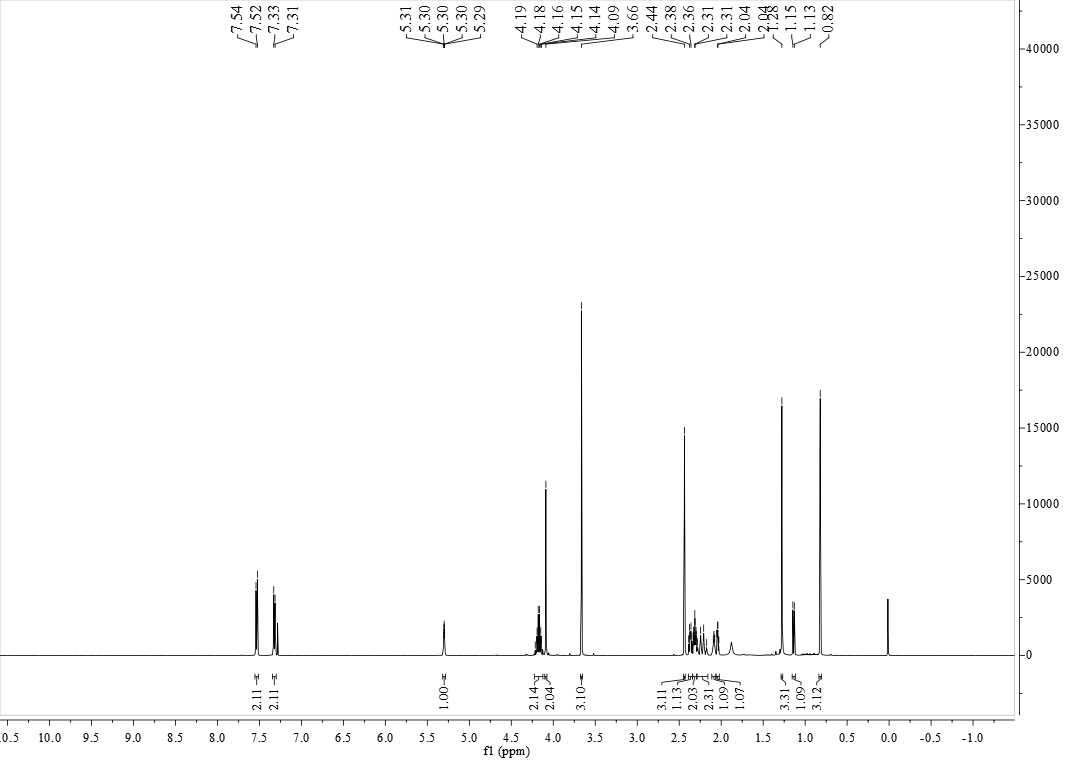


**FigureS11.** ^1^H NMR (500 MHz, CDCl_3_) of compound **5d**


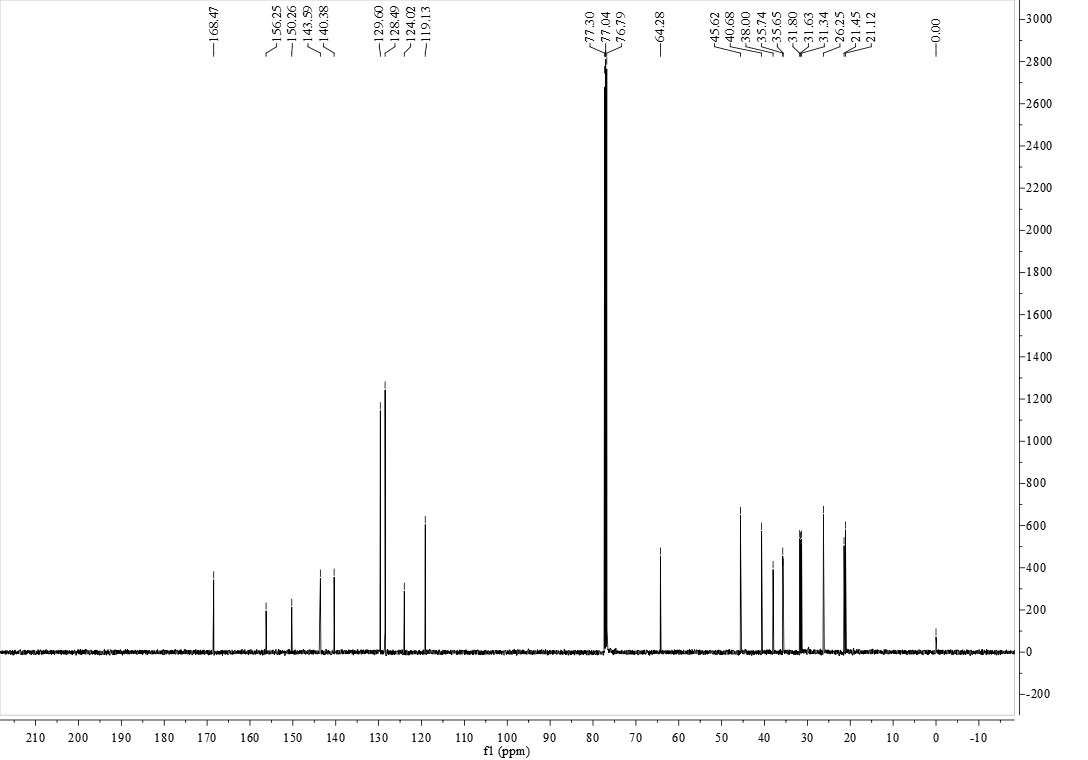


**FigureS12.** ^13^C NMR (125 MHz, CDCl_3_) of compound **5d**


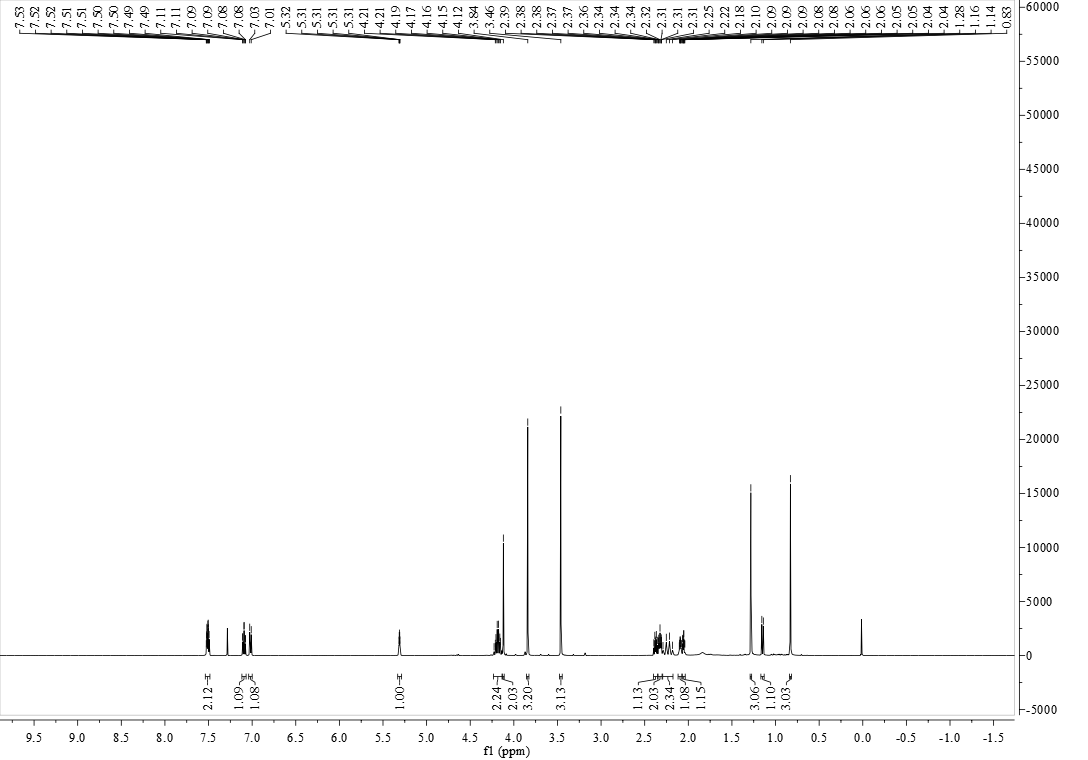


**FigureS13.** ^1^H NMR (500 MHz, CDCl_3_) of compound **5e**


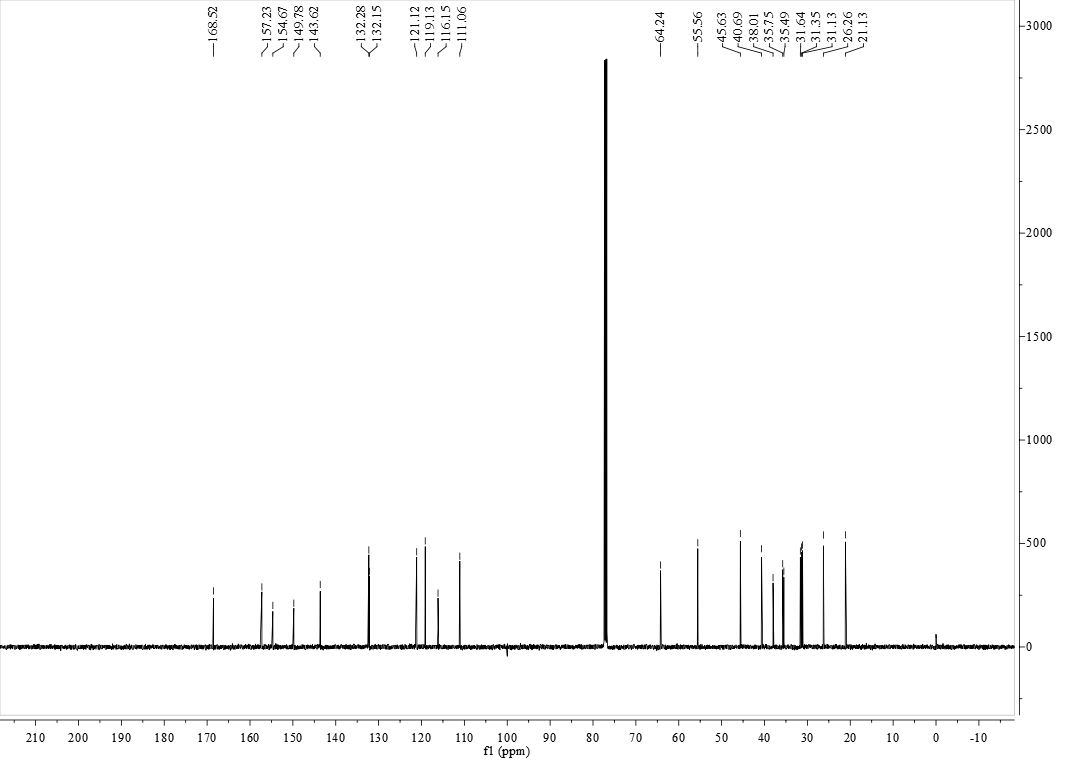


**FigureS14.** ^13^C NMR (125 MHz, CDCl_3_) of compound **5e**


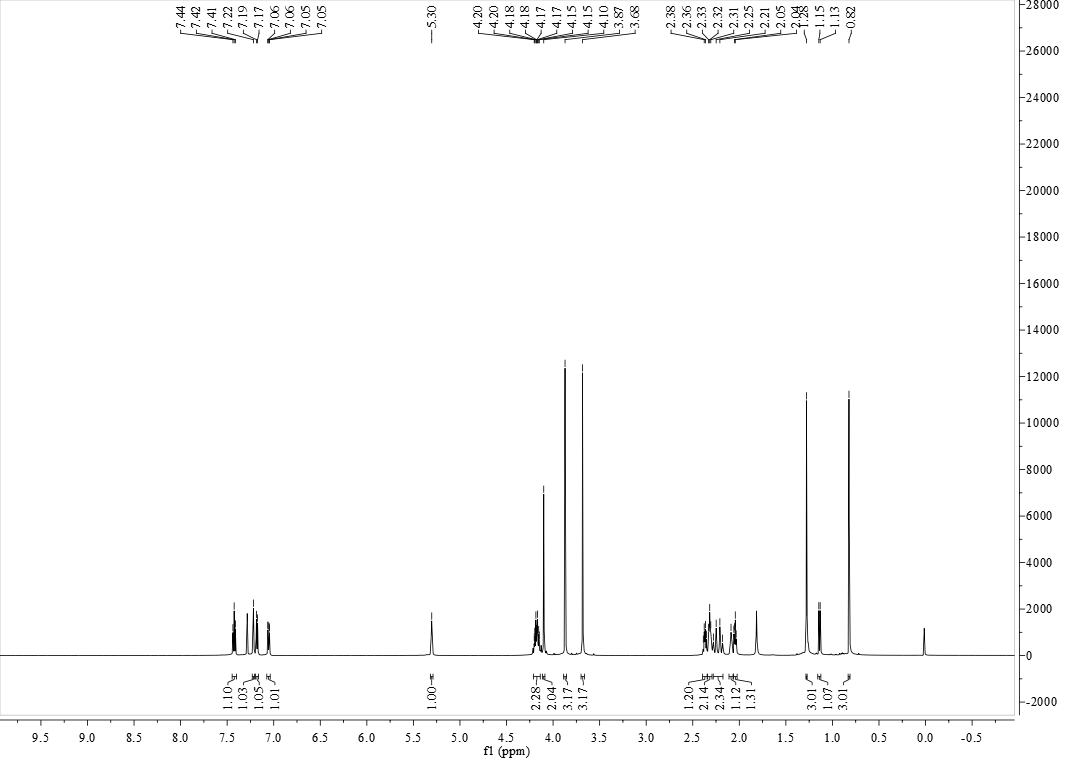


**FigureS15.** ^1^H NMR (600 MHz, CDCl_3_) of compound **5f**


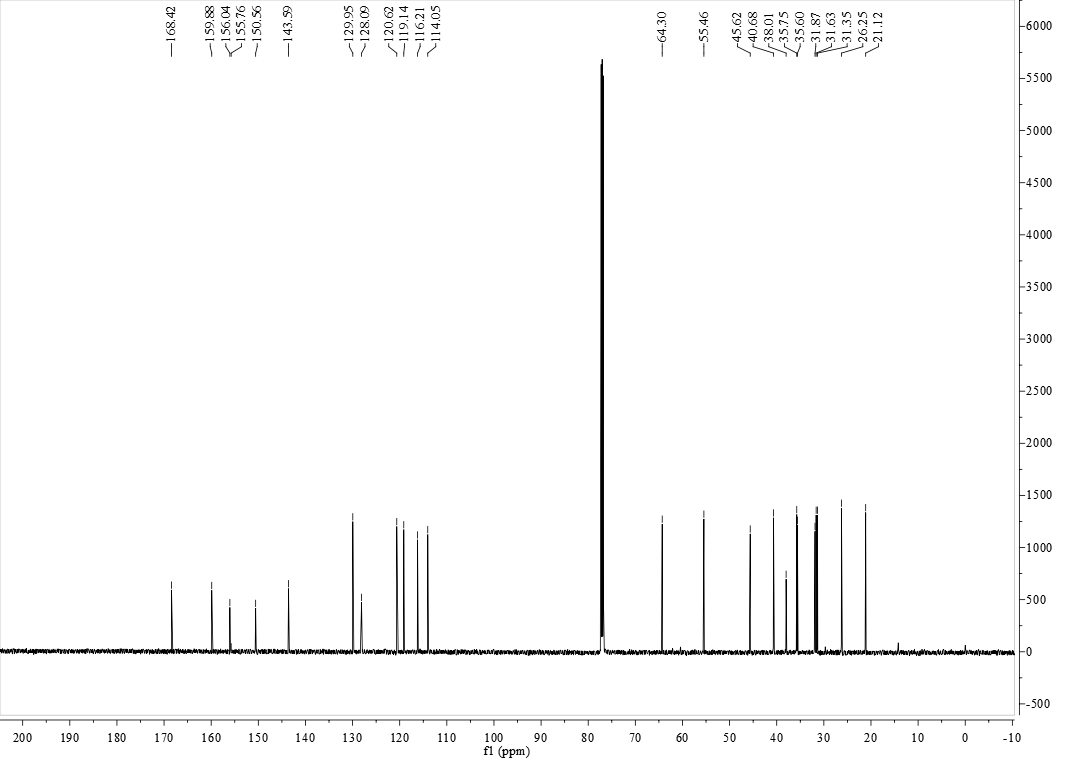


**FigureS16.** ^13^C NMR (150 MHz, CDCl_3_) of compound **5f**


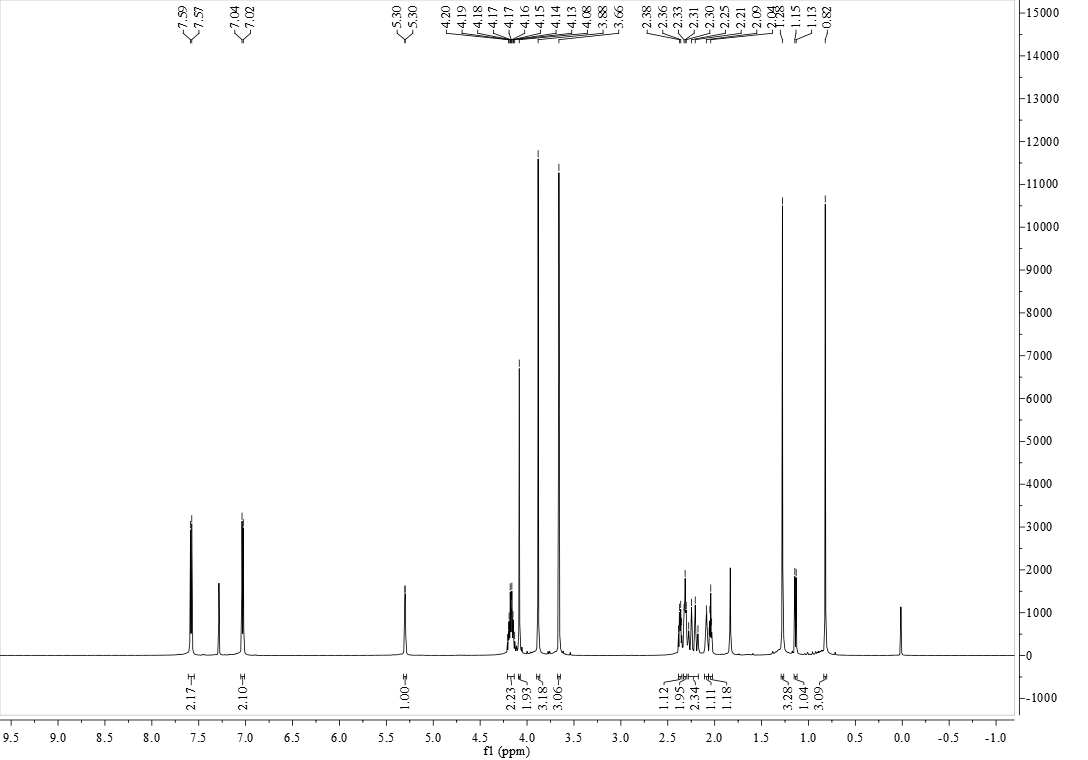


**FigureS17.** ^1^H NMR (600 MHz, CDCl_3_) of compound **5g**


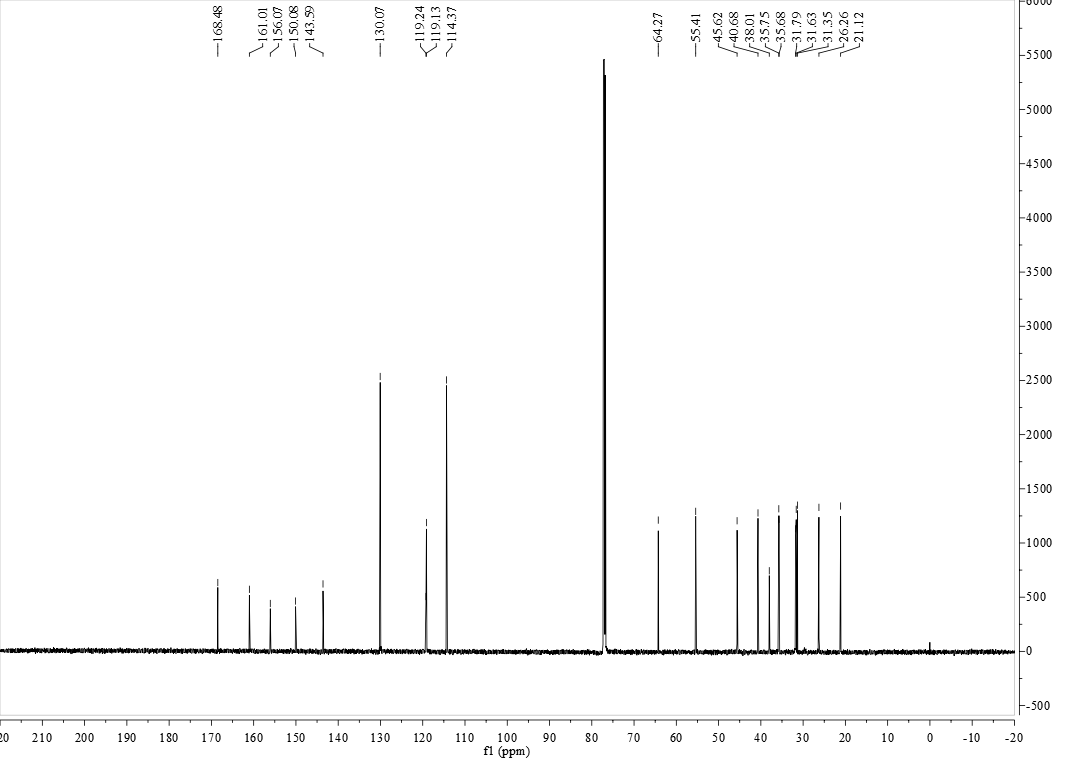


**FigureS18.** ^13^C NMR (150 MHz, CDCl_3_) of compound **5g**


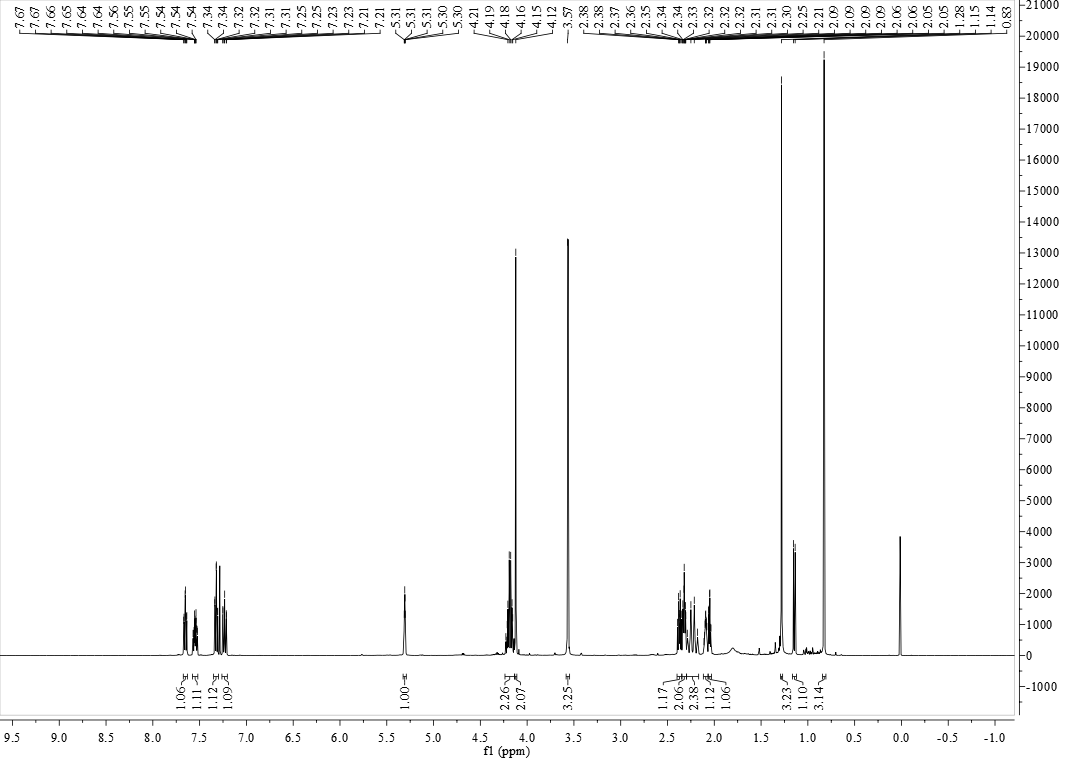


**FigureS19.** ^1^H NMR (500 MHz, CDCl_3_) of compound **5h**


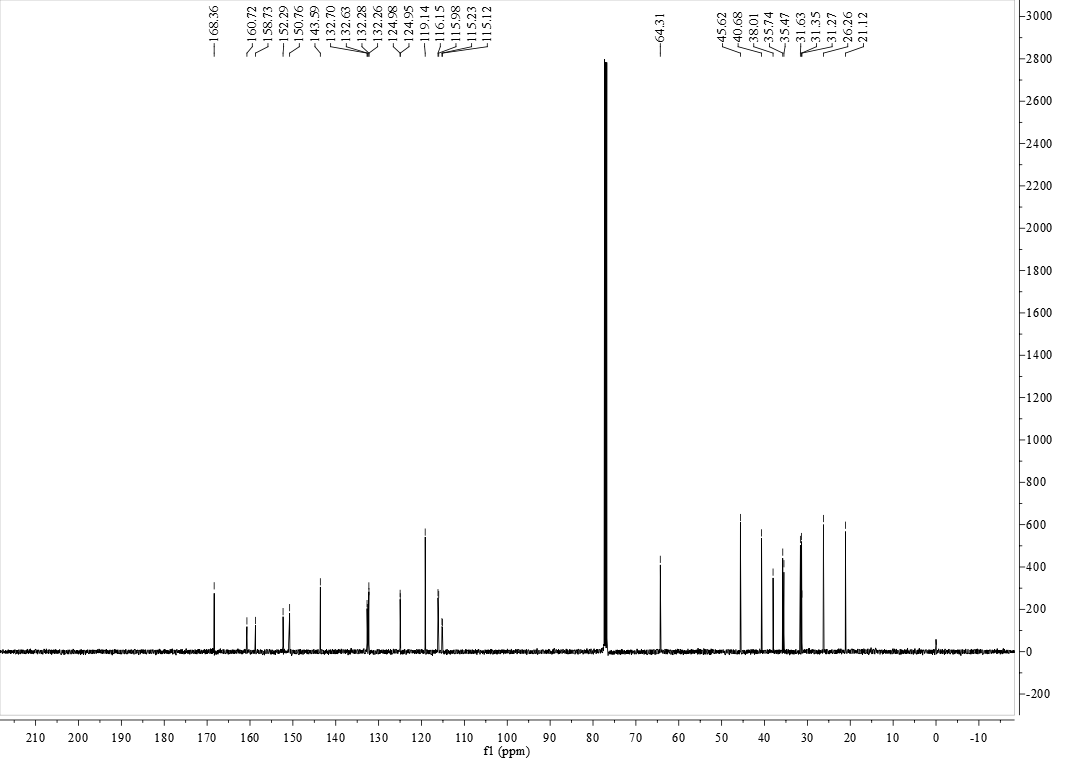


**FigureS20.** ^13^C NMR (125 MHz, CDCl_3_) of compound **5h**


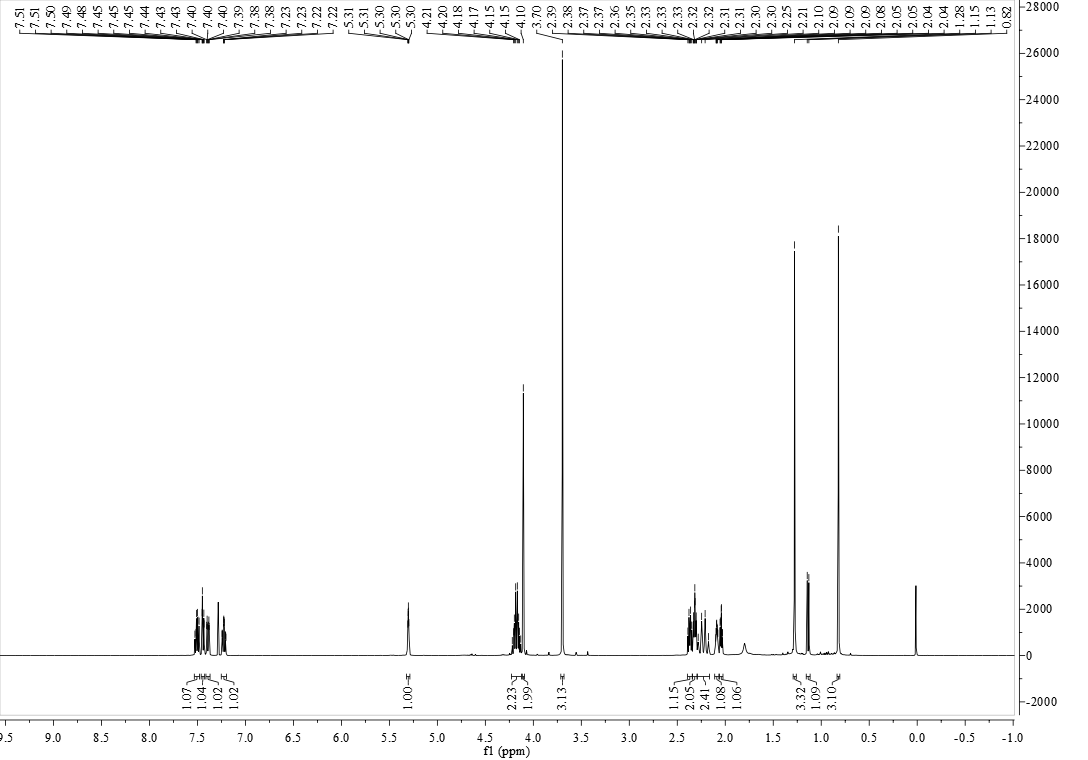


**FigureS21.** ^1^H NMR (500 MHz, CDCl_3_) of compound **5i**


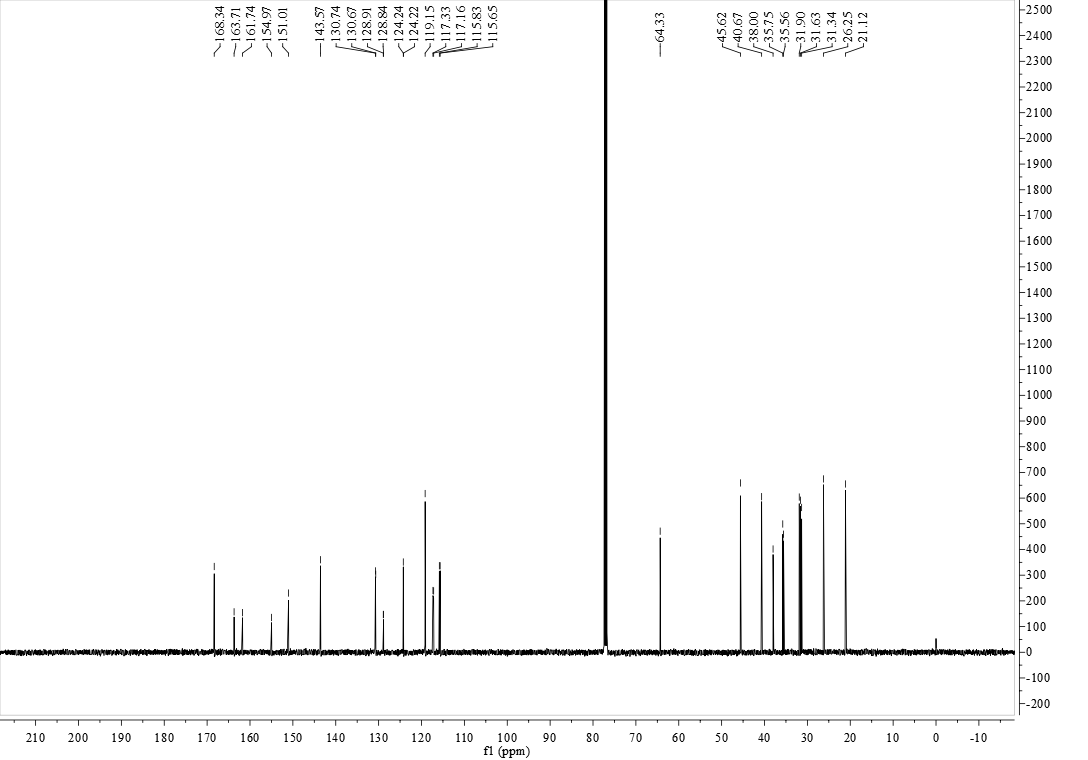


**FigureS22.** ^13^C NMR (125 MHz, CDCl_3_) of compound **5i**


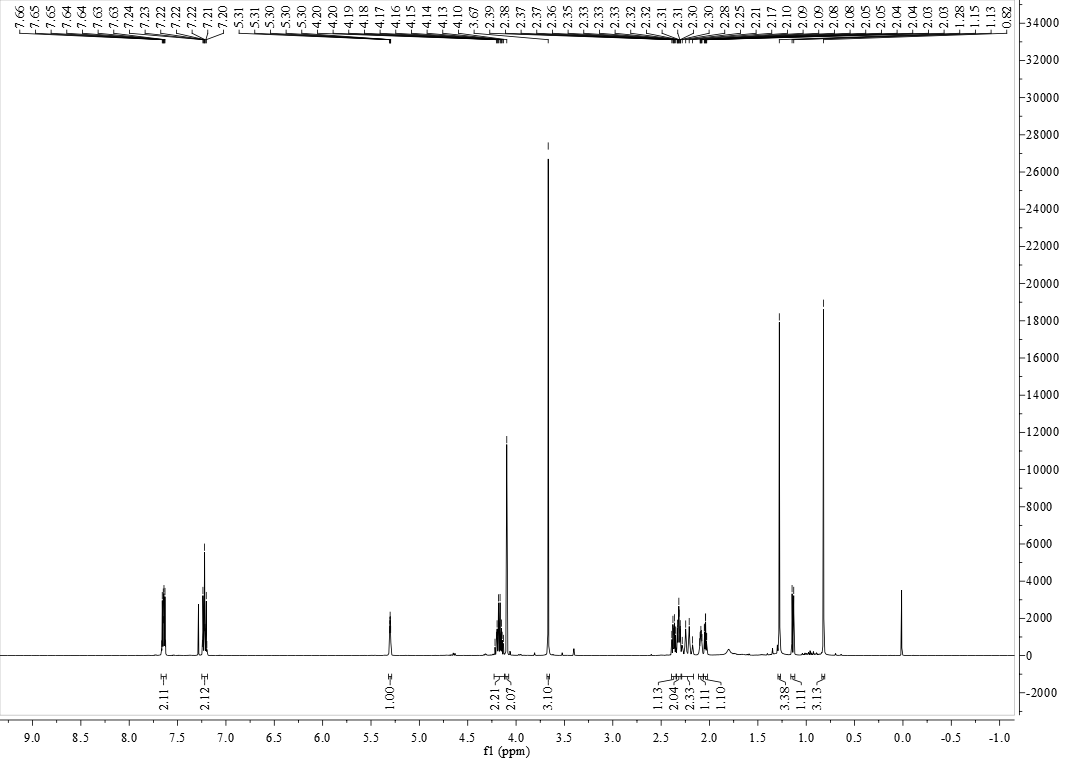


**FigureS23.** ^1^H NMR (500 MHz, CDCl_3_) of compound **5j**


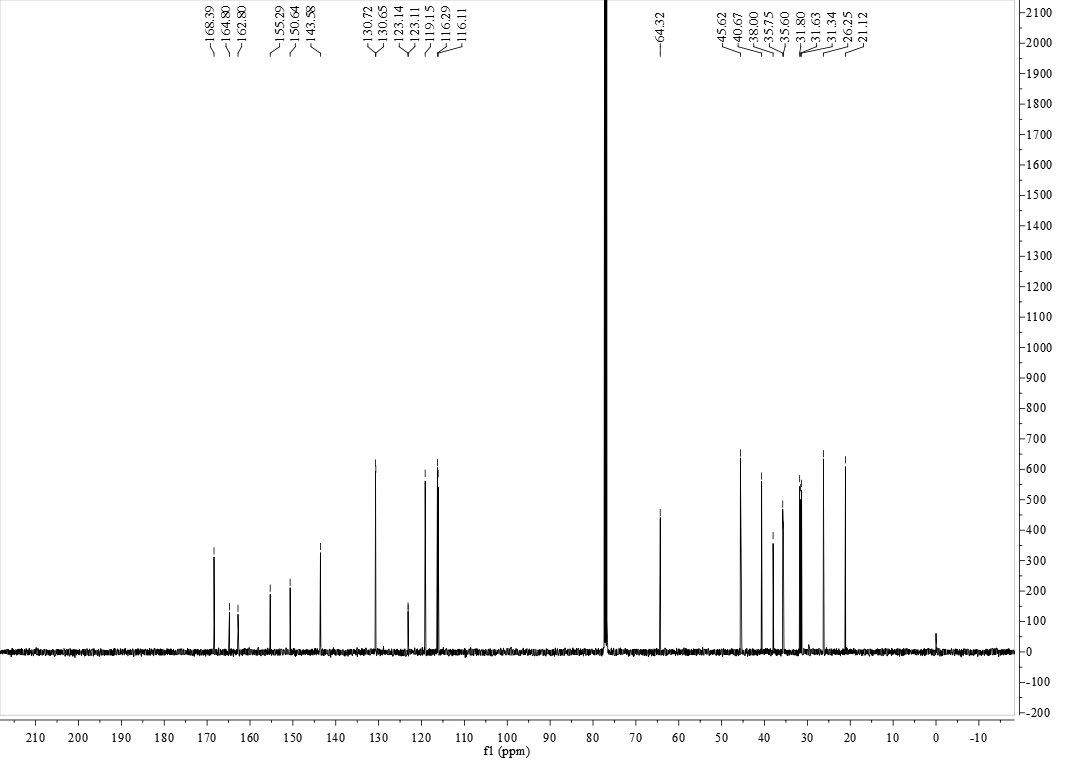


**FigureS24.** ^13^C NMR (125 MHz, CDCl_3_) of compound **5j**


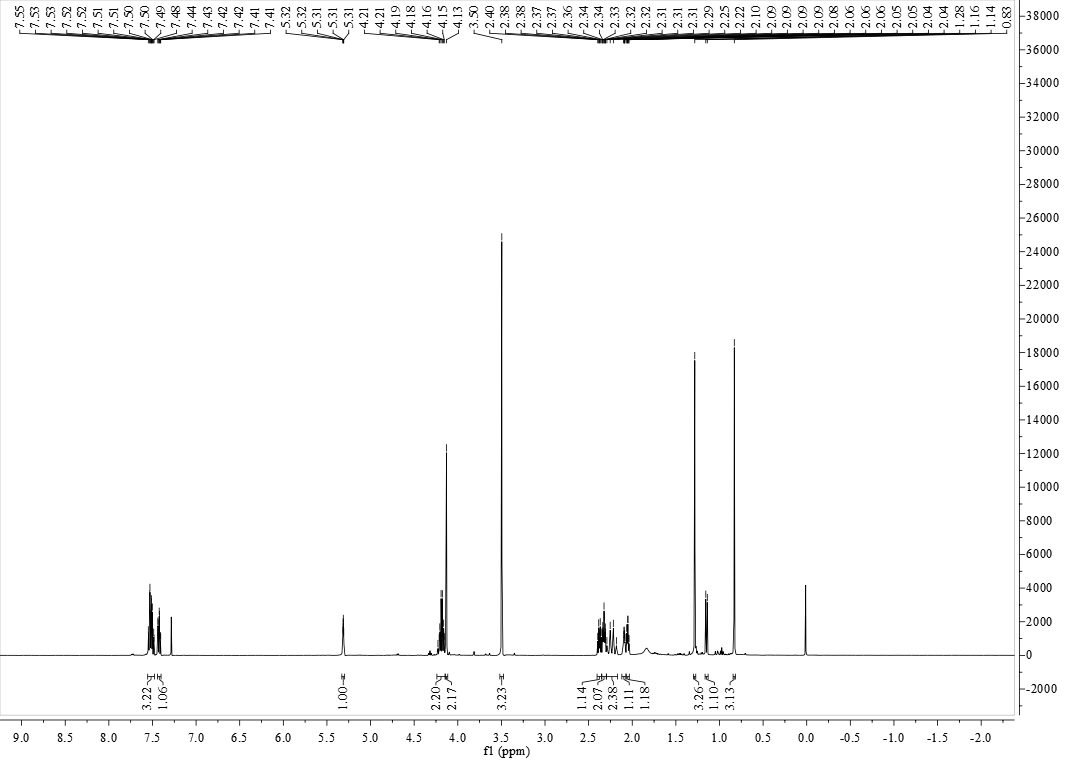


**FigureS25.** ^1^H NMR (500 MHz, CDCl_3_) of compound **5k**


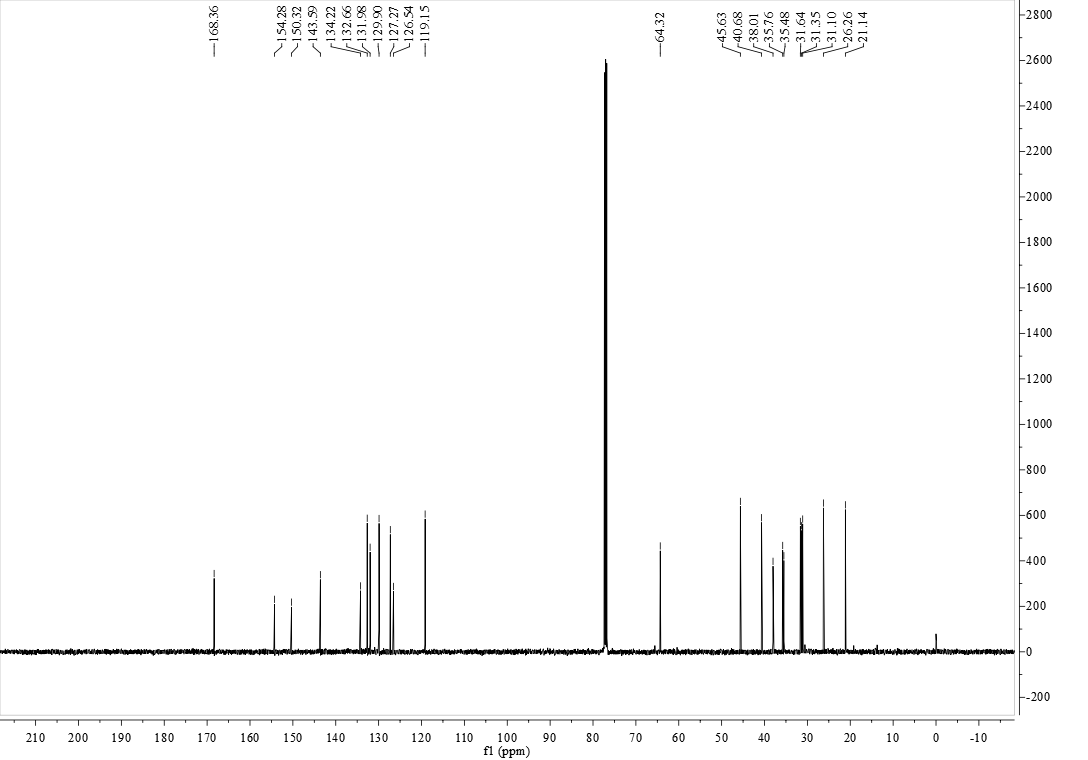


**FigureS26.** ^13^C NMR (125 MHz, CDCl_3_) of compound **5k**


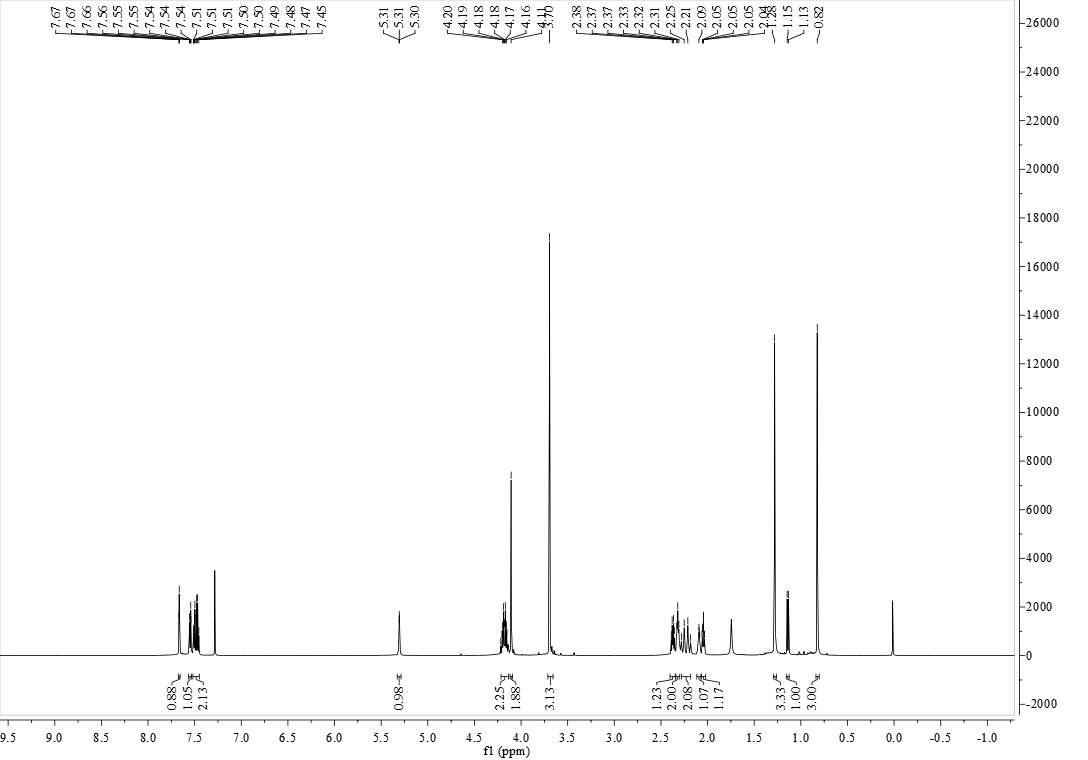


**FigureS27.** ^1^H NMR (600 MHz, CDCl_3_) of compound **5l**


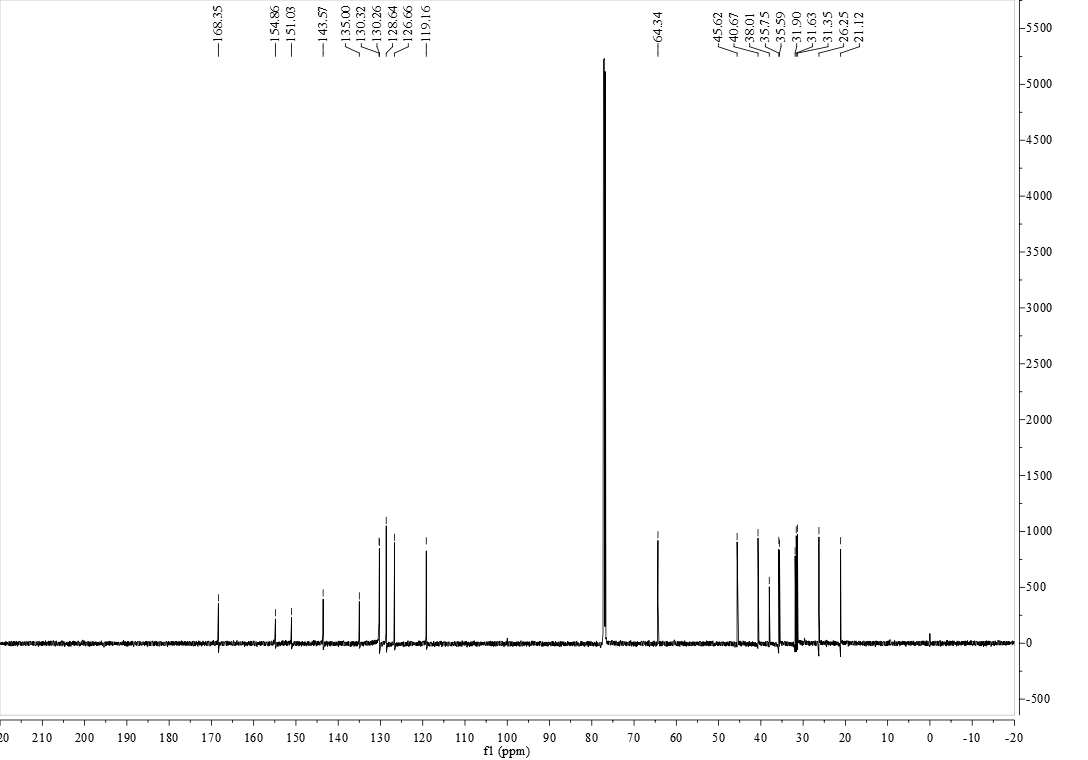


**FigureS28.** ^13^C NMR (150 MHz, CDCl_3_) of compound **5l**


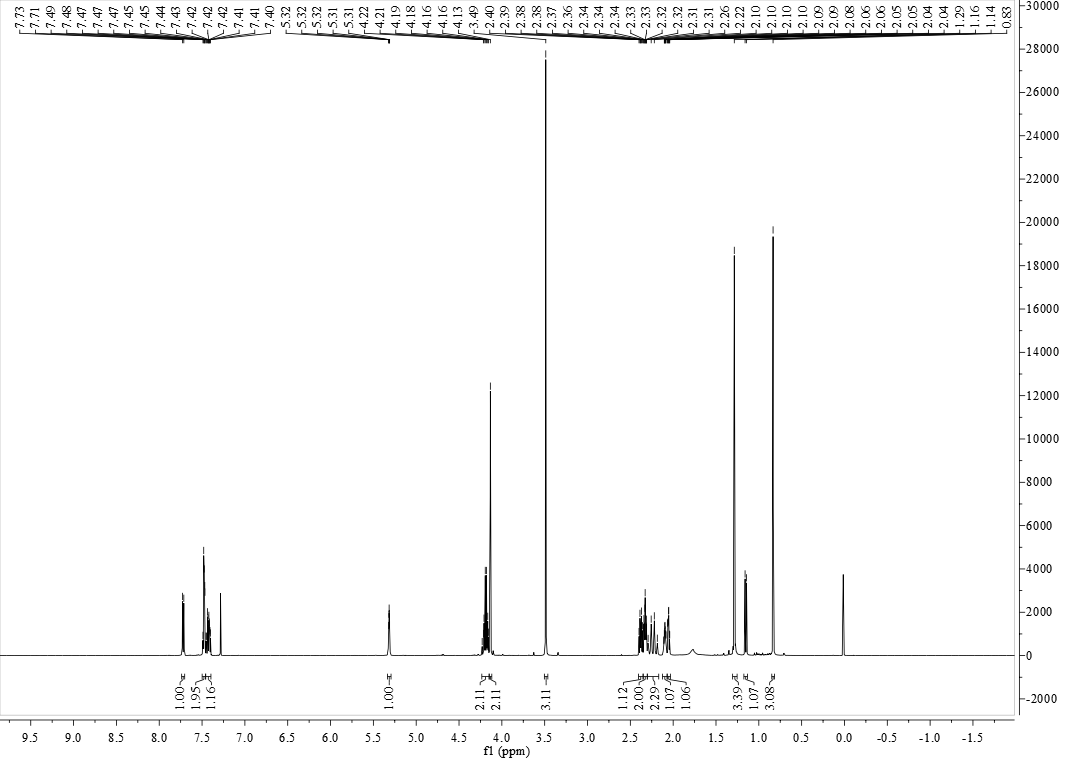


**FigureS29.** ^1^H NMR (500 MHz, CDCl_3_) of compound **5m**


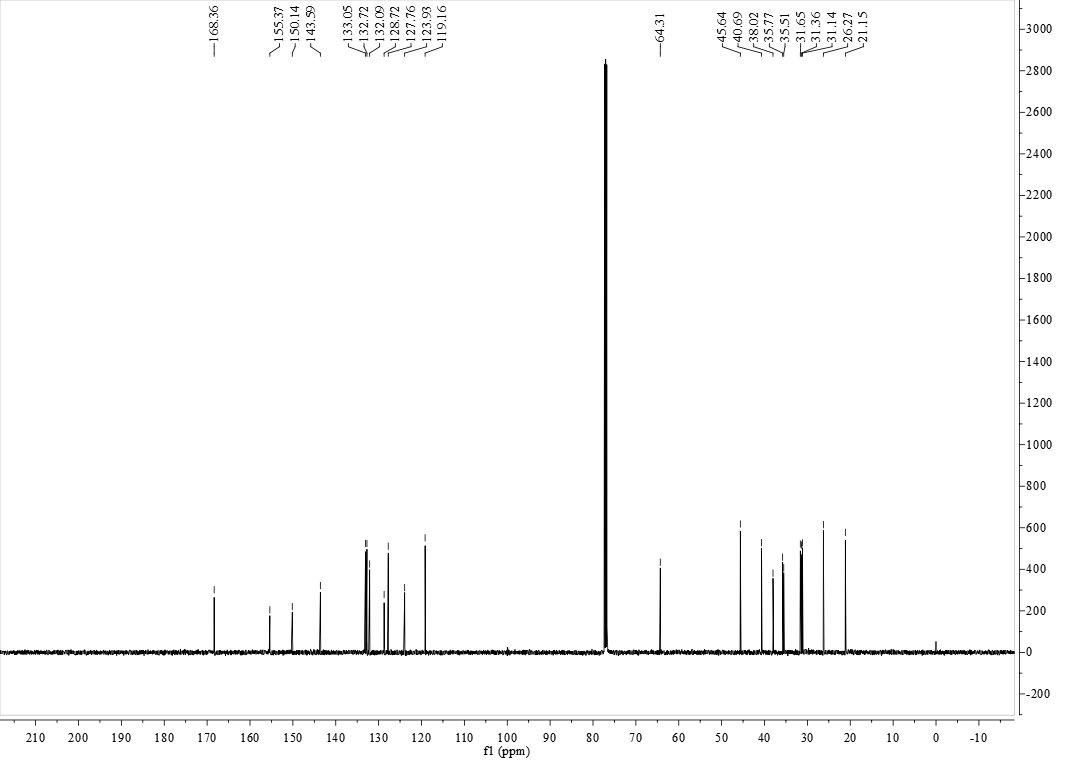


**FigureS30.** ^13^C NMR (125 MHz, CDCl_3_) of compound **5m**


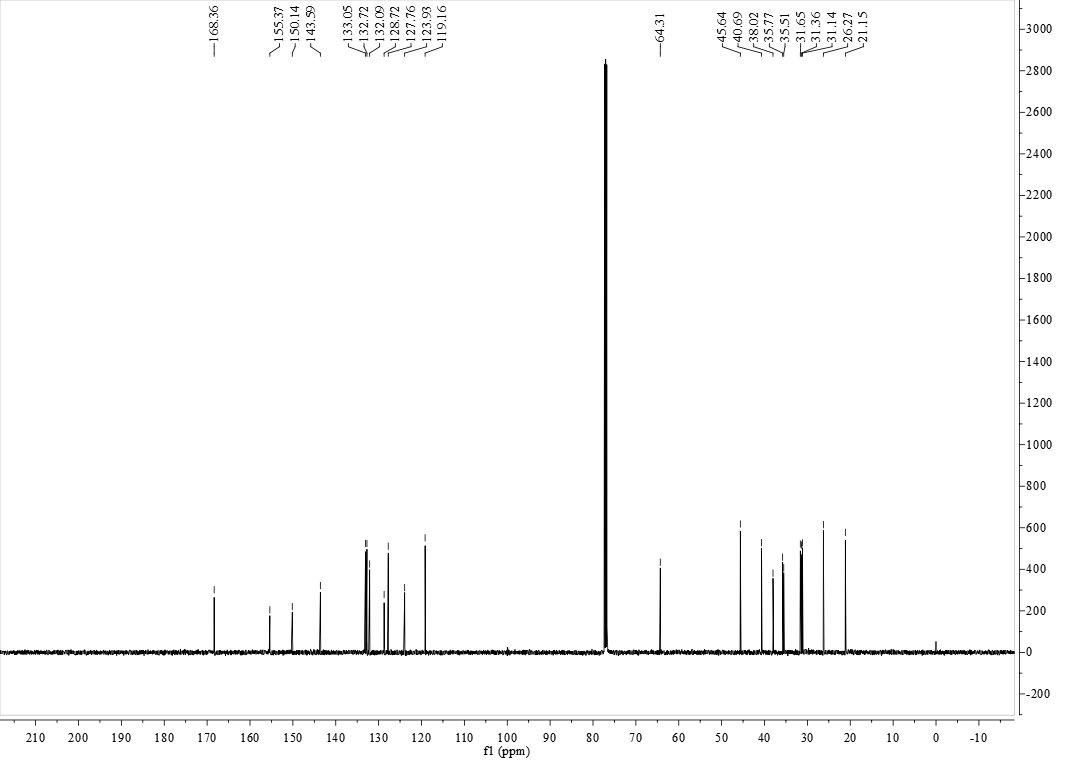


**FigureS31.** ^1^H NMR (500 MHz, CDCl_3_) of compound **5n**


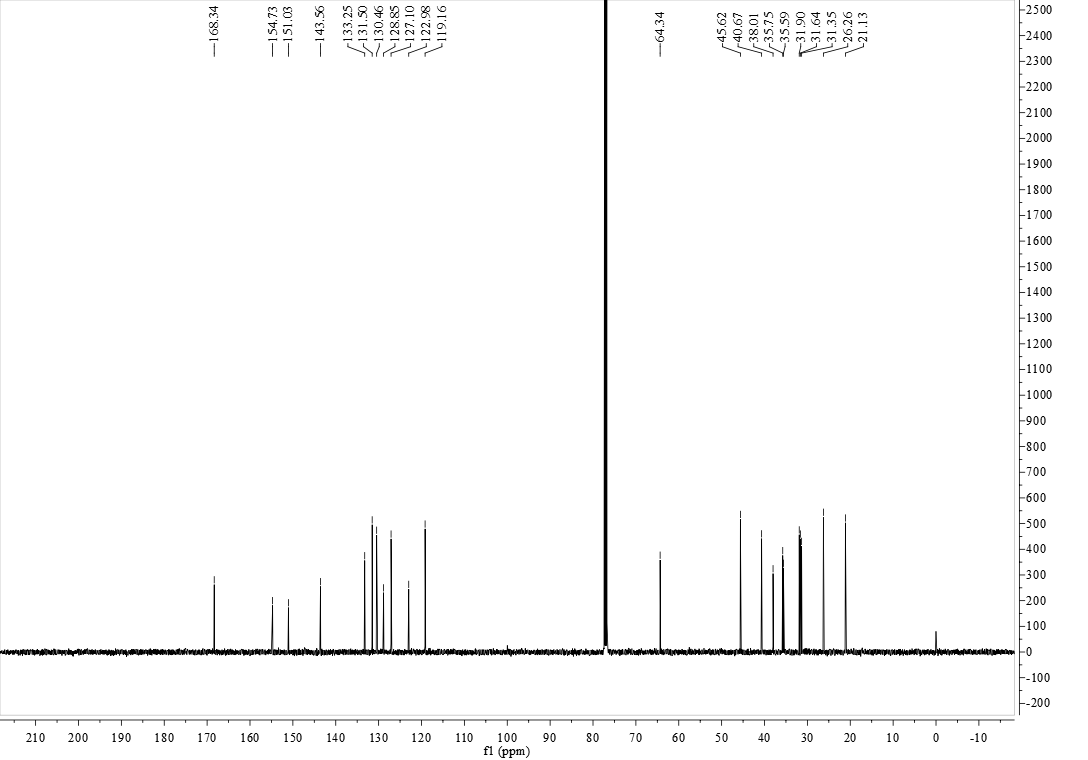


**FigureS32.** ^13^C NMR (125 MHz, CDCl_3_) of compound **5n**


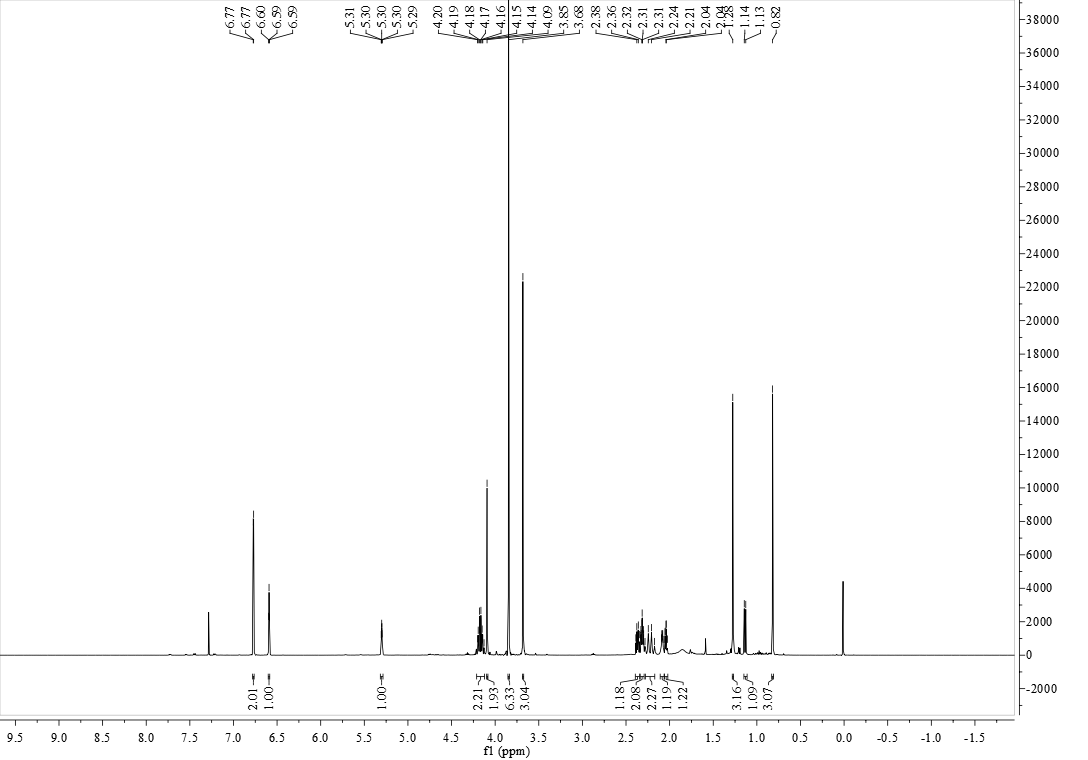


**FigureS33.** ^1^H NMR (500 MHz, CDCl_3_) of compound **5o**


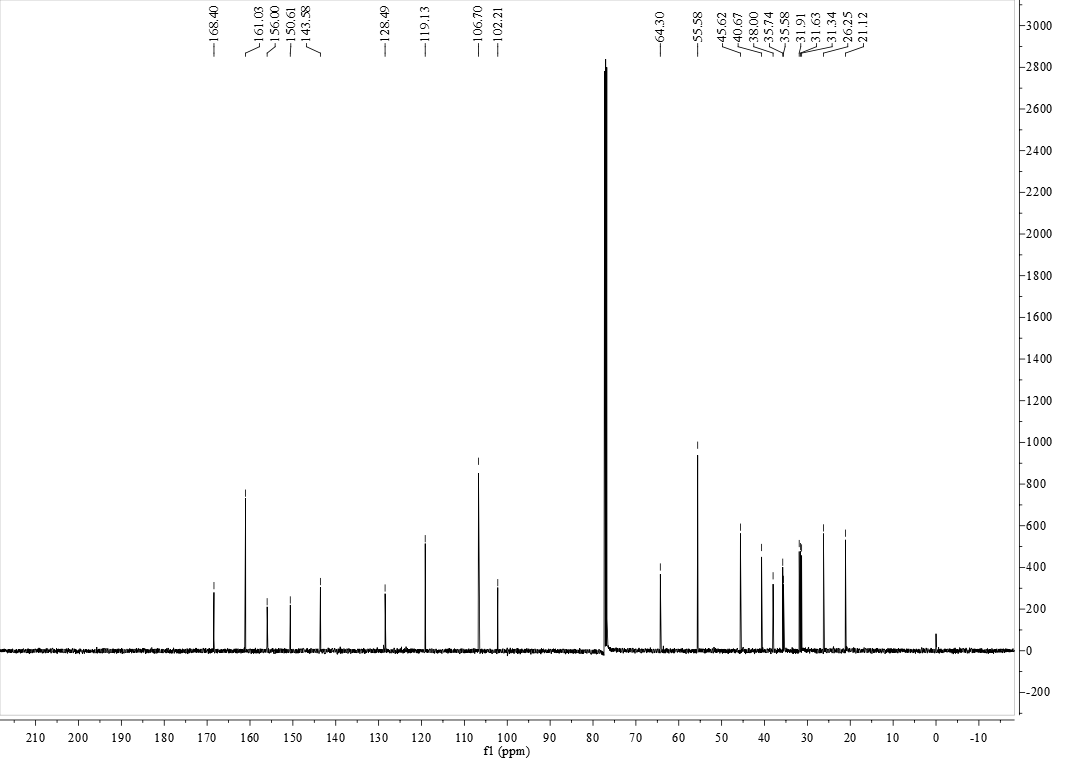


**FigureS34.** ^13^C NMR (125 MHz, CDCl_3_) of compound **5o**


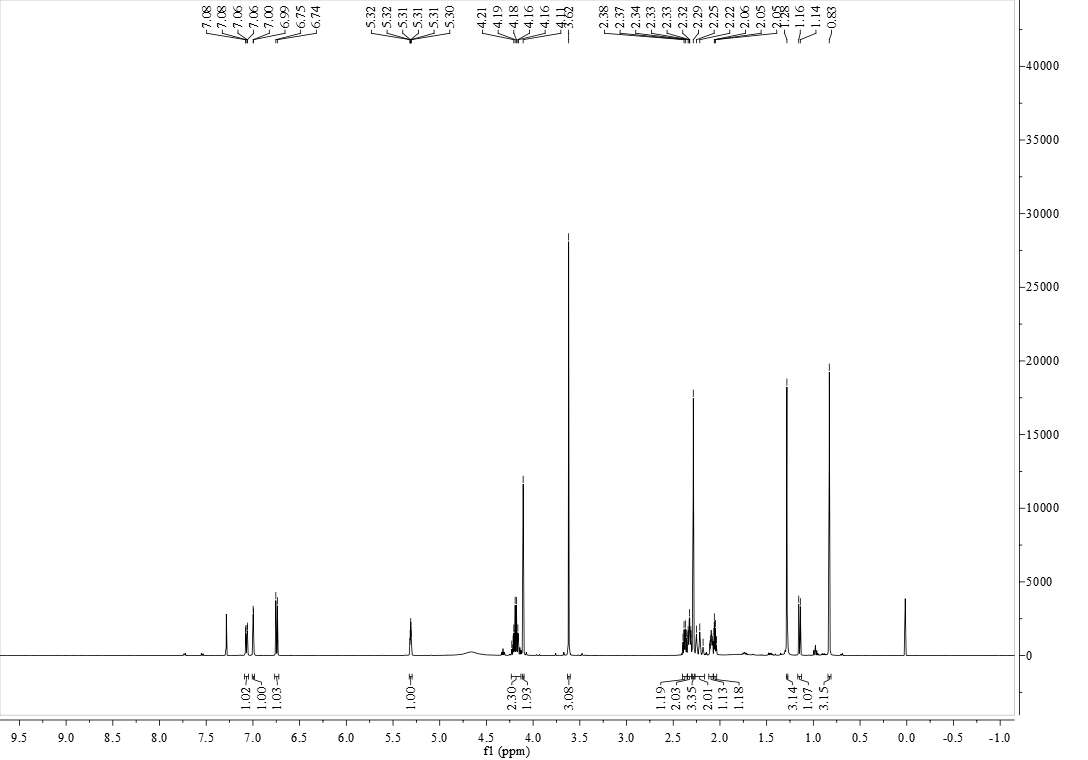


**FigureS35.** ^1^H NMR (500 MHz, CDCl_3_) of compound **5p**


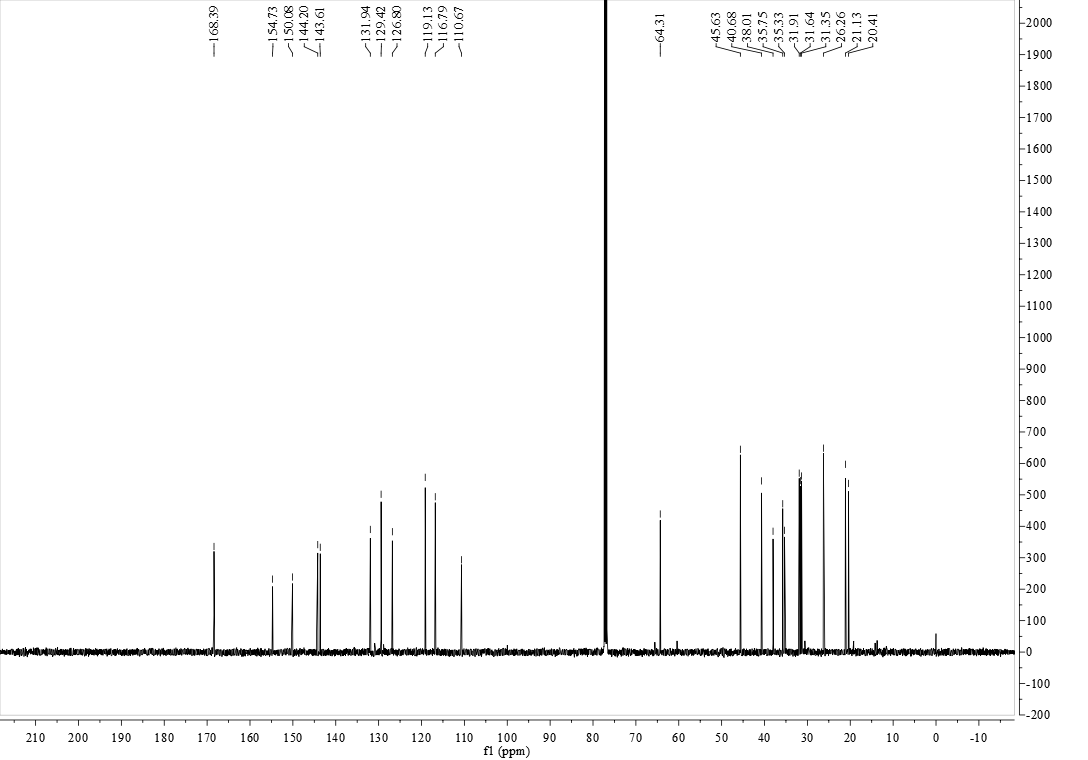


**FigureS36.** ^13^C NMR (125 MHz, CDCl_3_) of compound **5p**


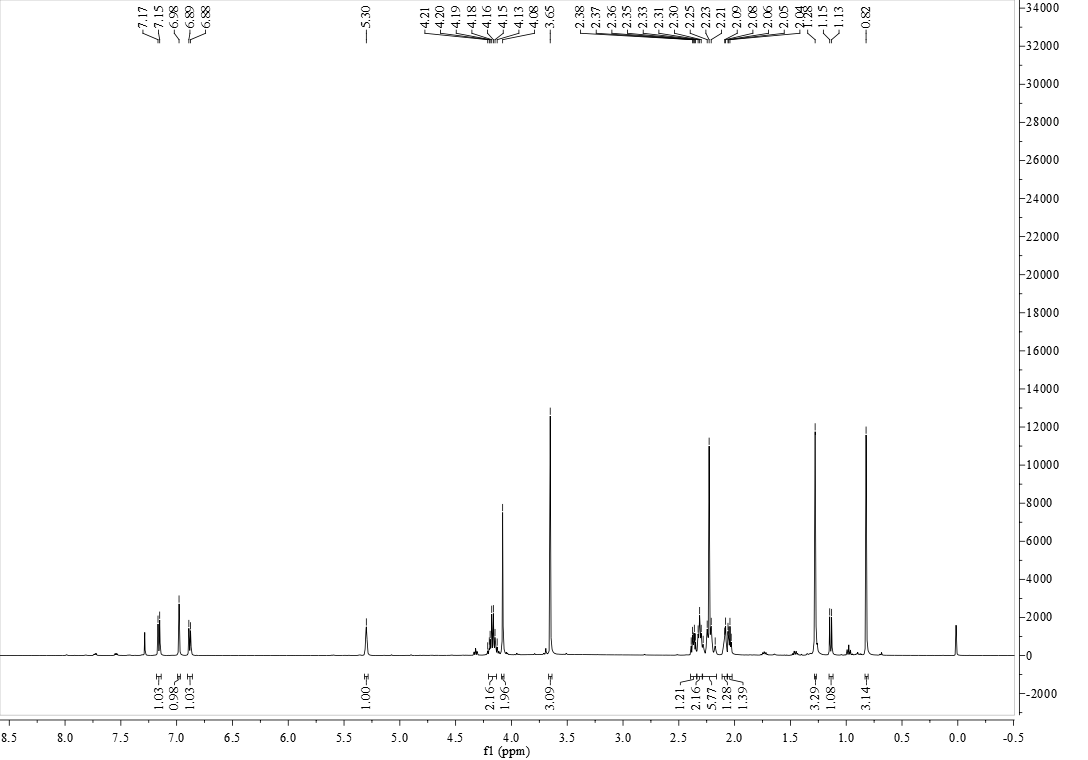


**FigureS37.** ^1^H NMR (500 MHz, CDCl_3_) of compound **5q**


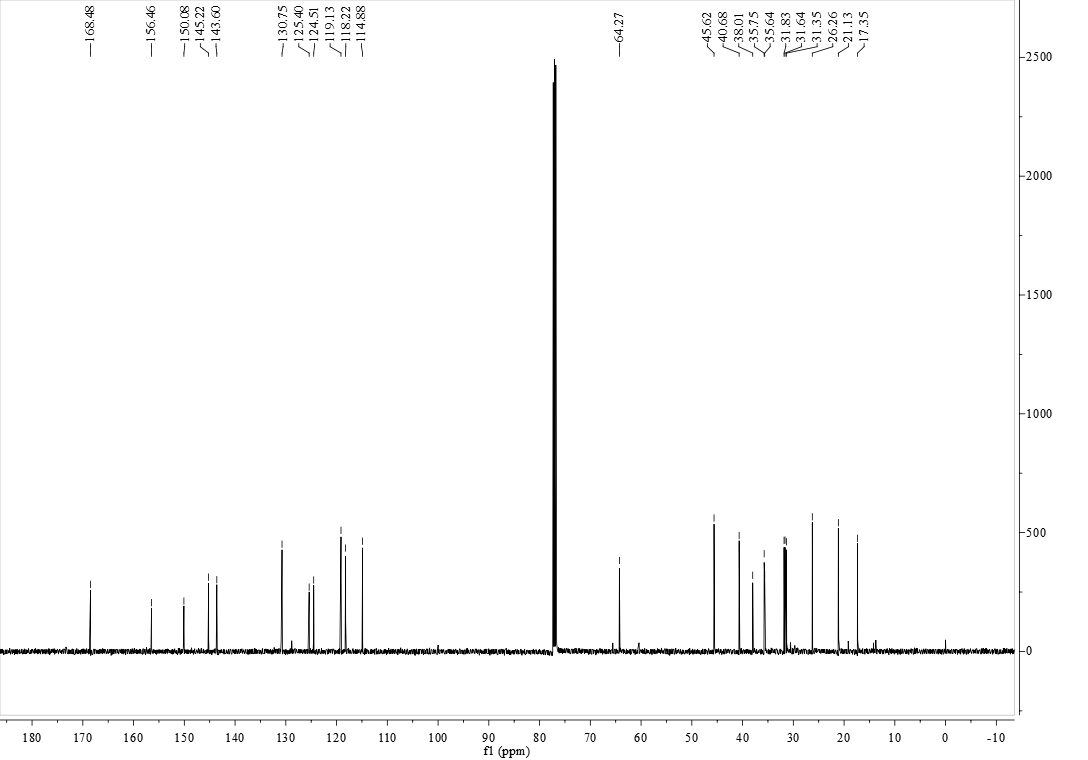


**FigureS38.** ^13^C NMR (125 MHz, CDCl_3_) of compound **5q**


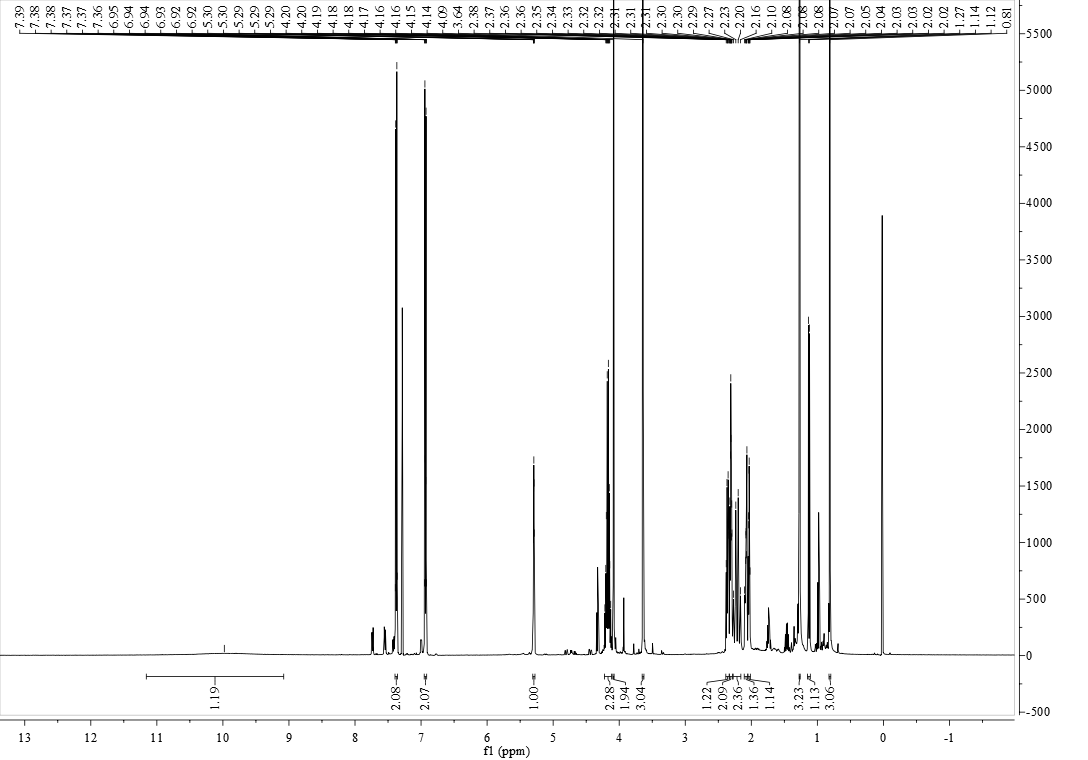


**FigureS39.** ^1^H NMR (500 MHz, CDCl_3_) of compound **5r**


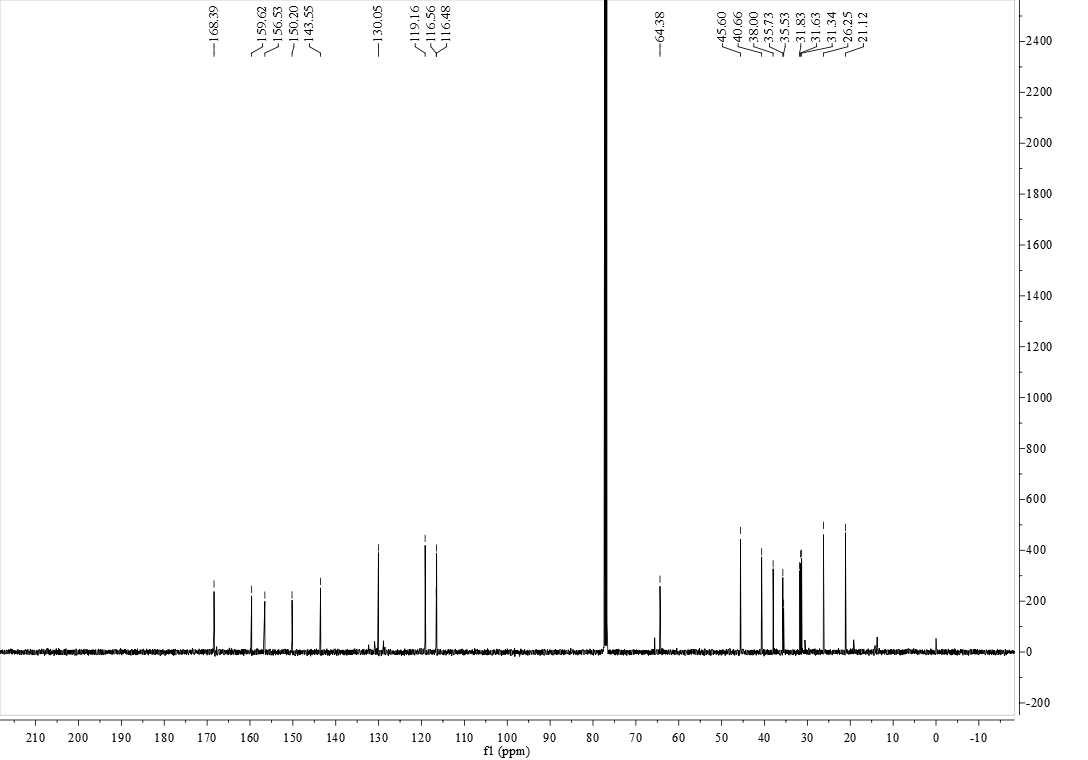


**FigureS40.** ^13^C NMR (125 MHz, CDCl_3_) of compound **5r**


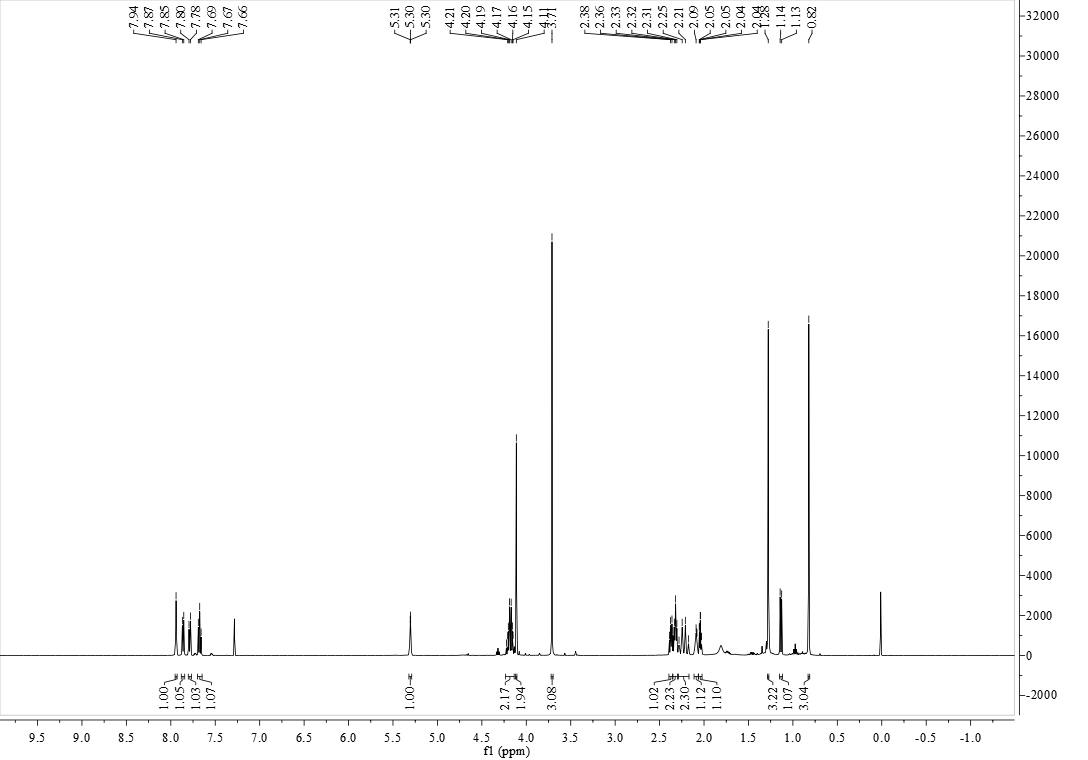


**FigureS41.** ^1^H NMR (500 MHz, CDCl_3_) of compound **5s**


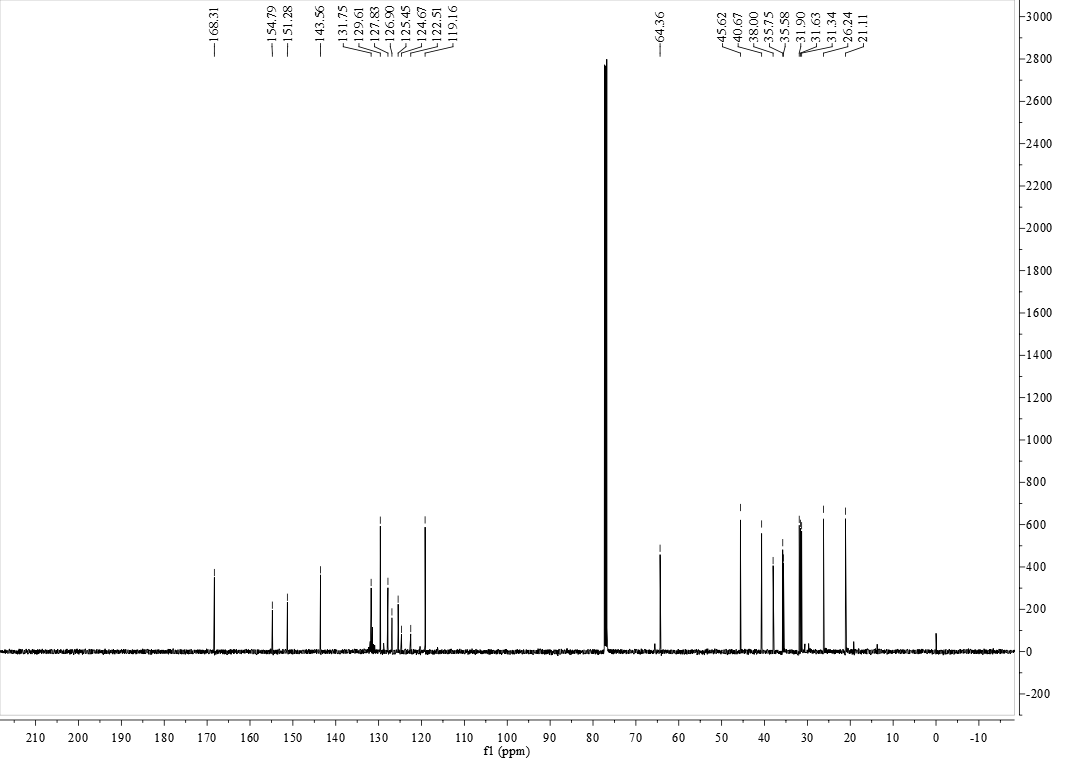


**FigureS42.** ^13^C NMR (125 MHz, CDCl_3_) of compound **5s**


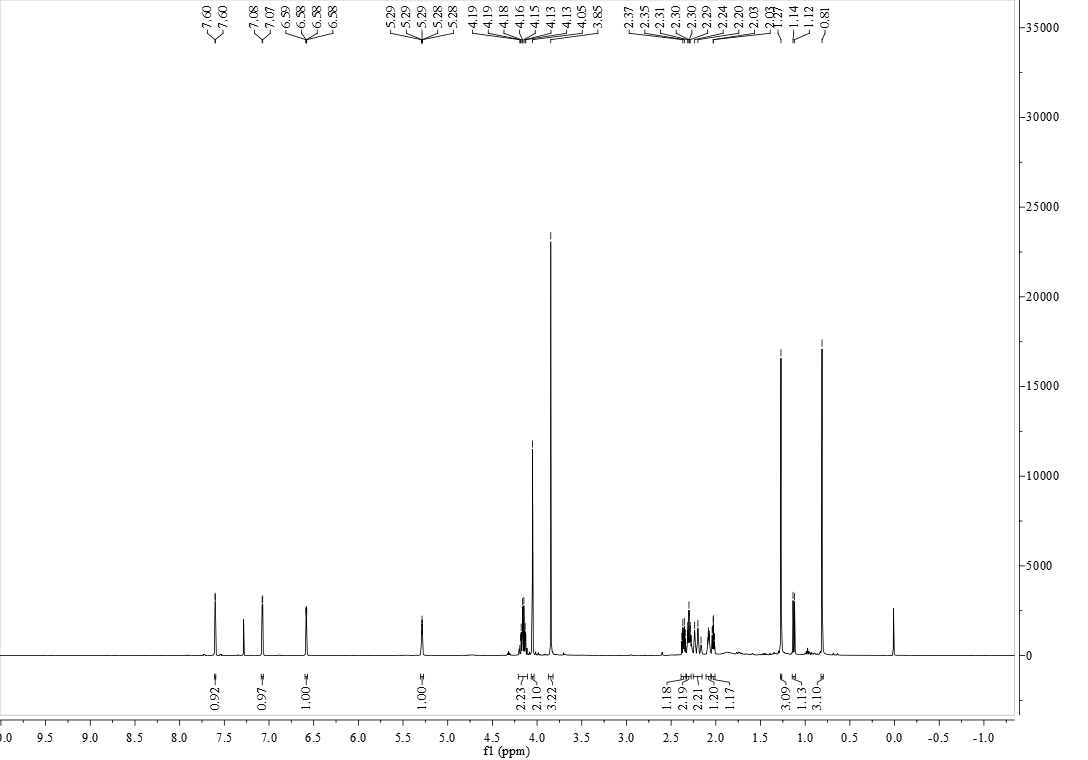


**FigureS43.** ^1^H NMR (500 MHz, CDCl_3_) of compound **5t**


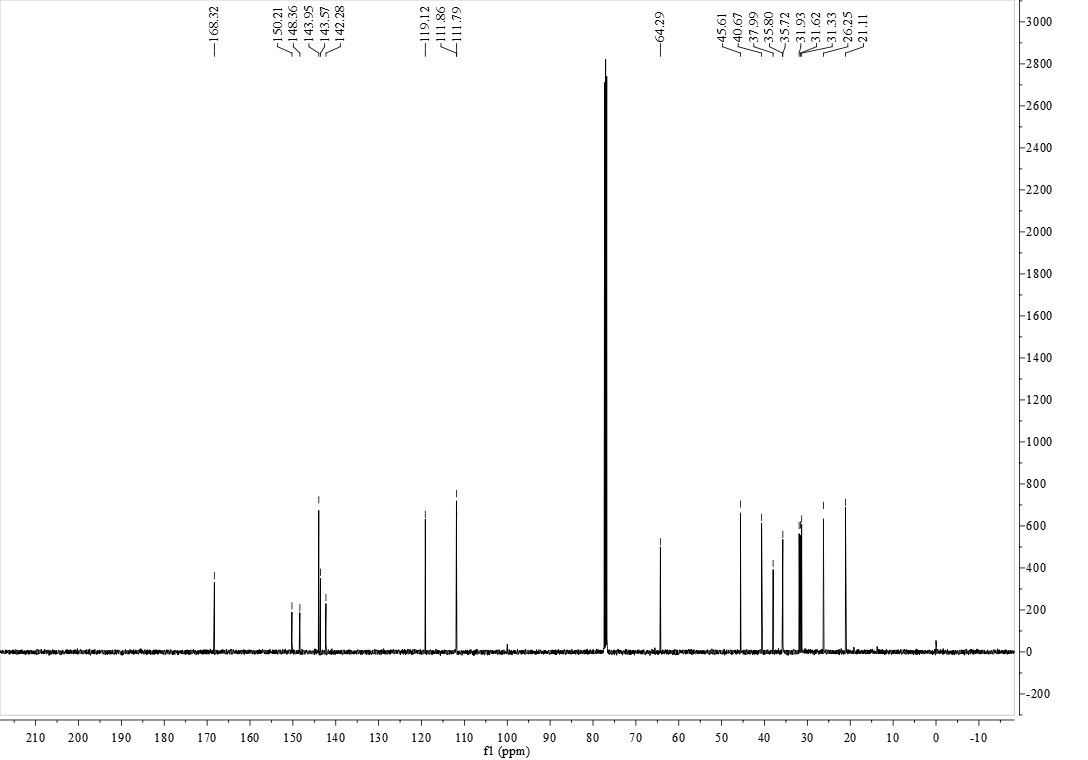


**FigureS44.** ^13^C NMR (125 MHz, CDCl_3_) of compound **5t**


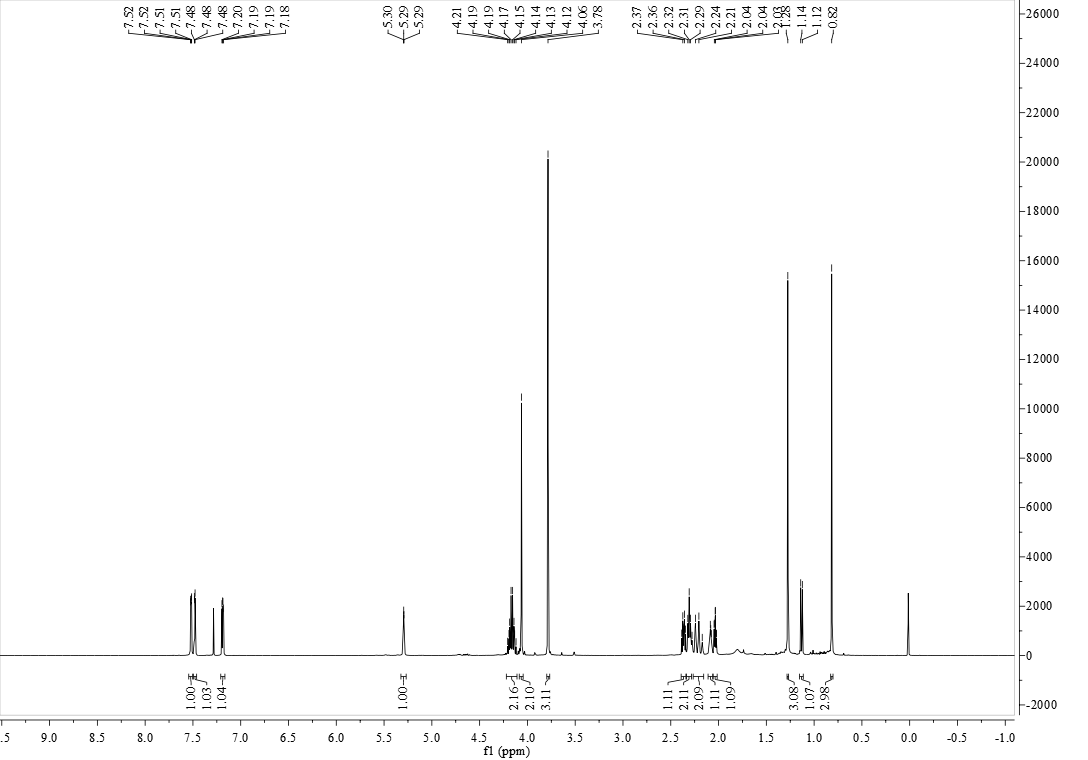


**FigureS45.** ^1^H NMR (500 MHz, CDCl_3_) of compound **5u**


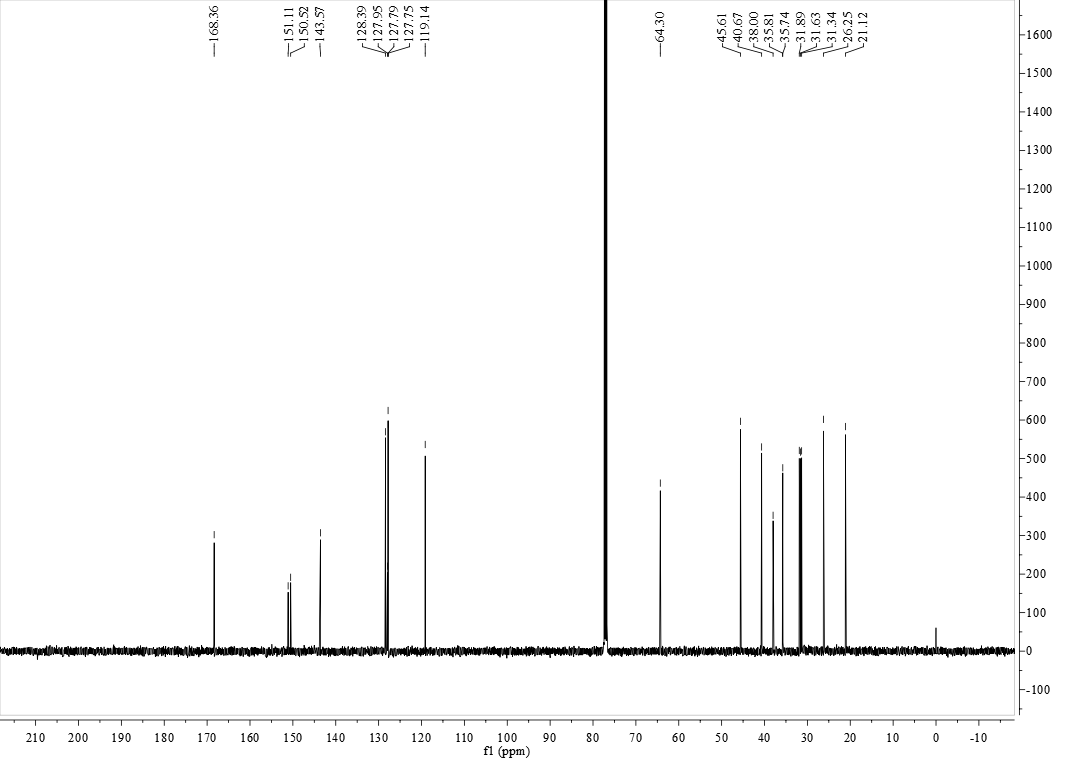


**FigureS46.** ^13^C NMR (125 MHz, CDCl_3_) of compound **5u**


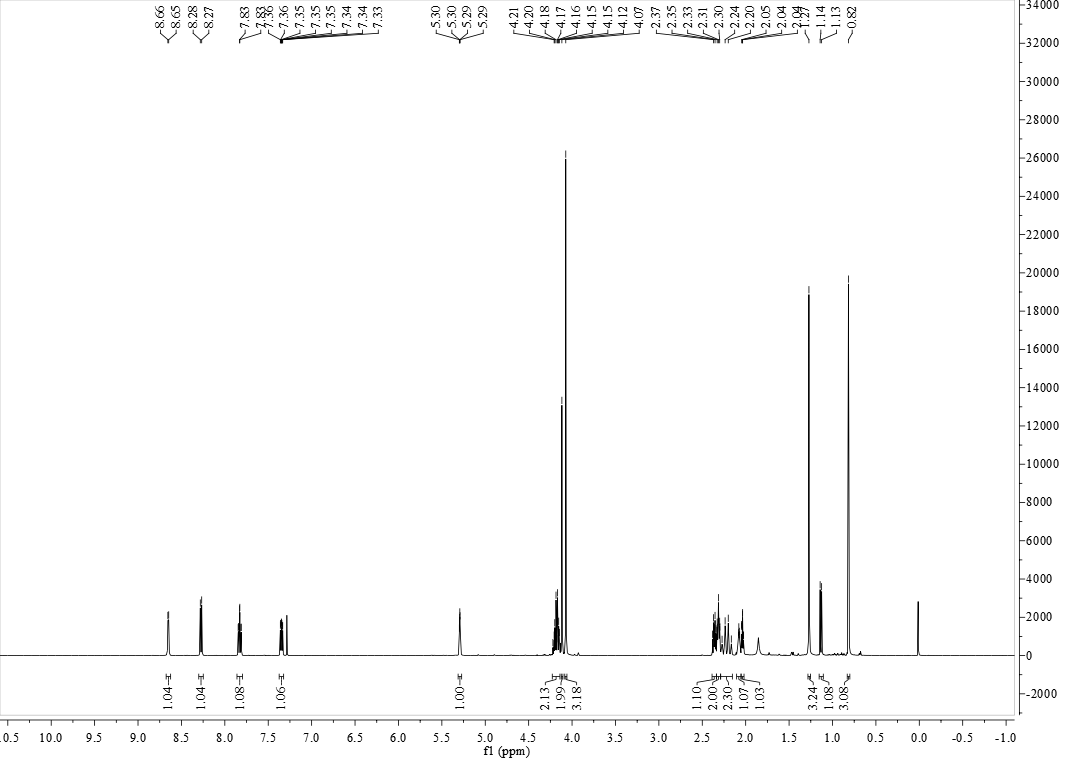


**FigureS47.** ^1^H NMR (500 MHz, CDCl_3_) of compound **5v**


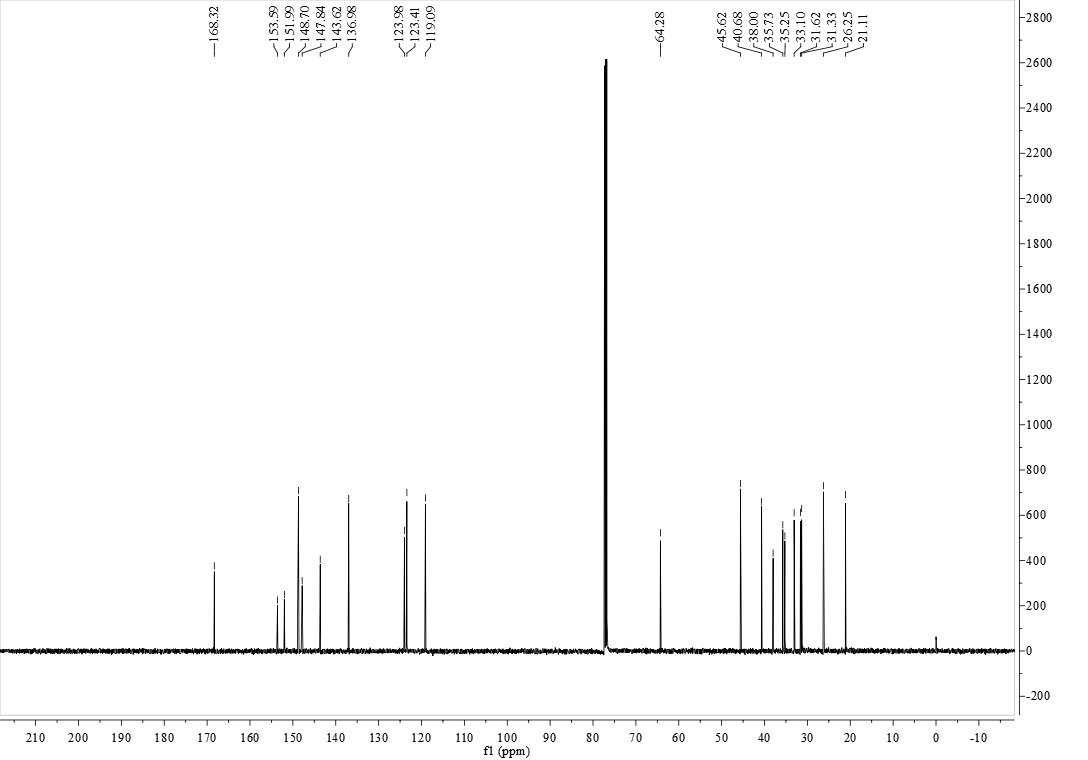


**FigureS48.** ^13^C NMR (125 MHz, CDCl_3_) of compound **5v**


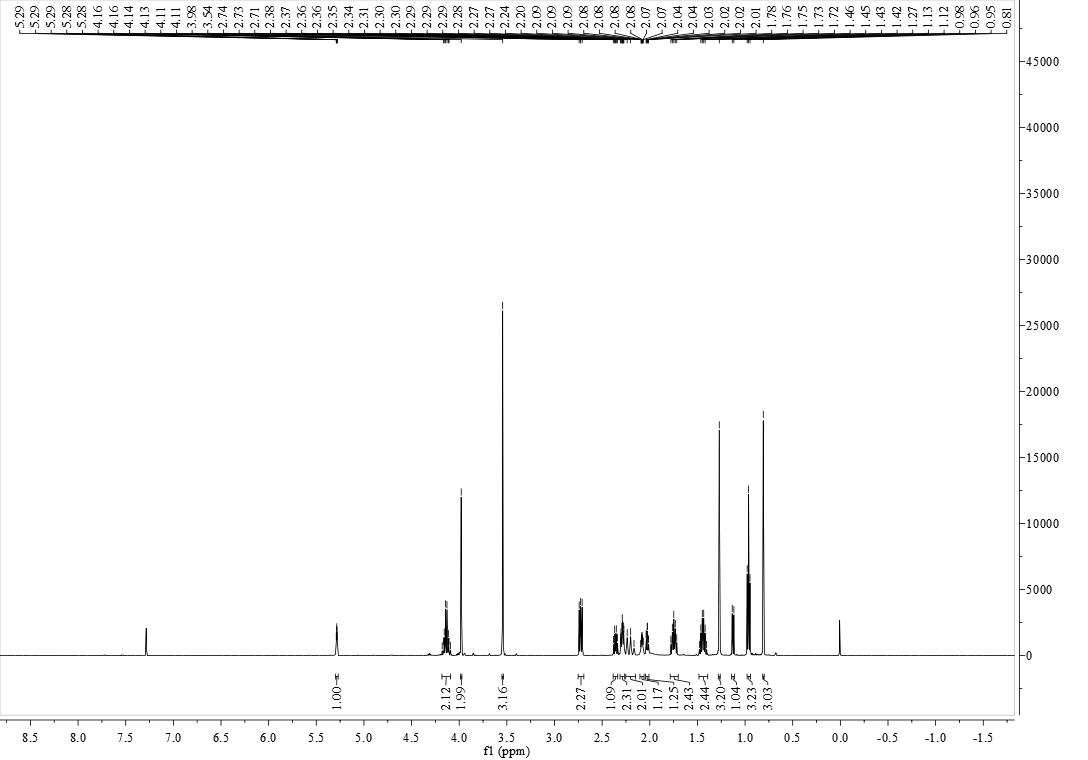


**FigureS49.** ^1^H NMR (500 MHz, CDCl_3_) of compound **5w**


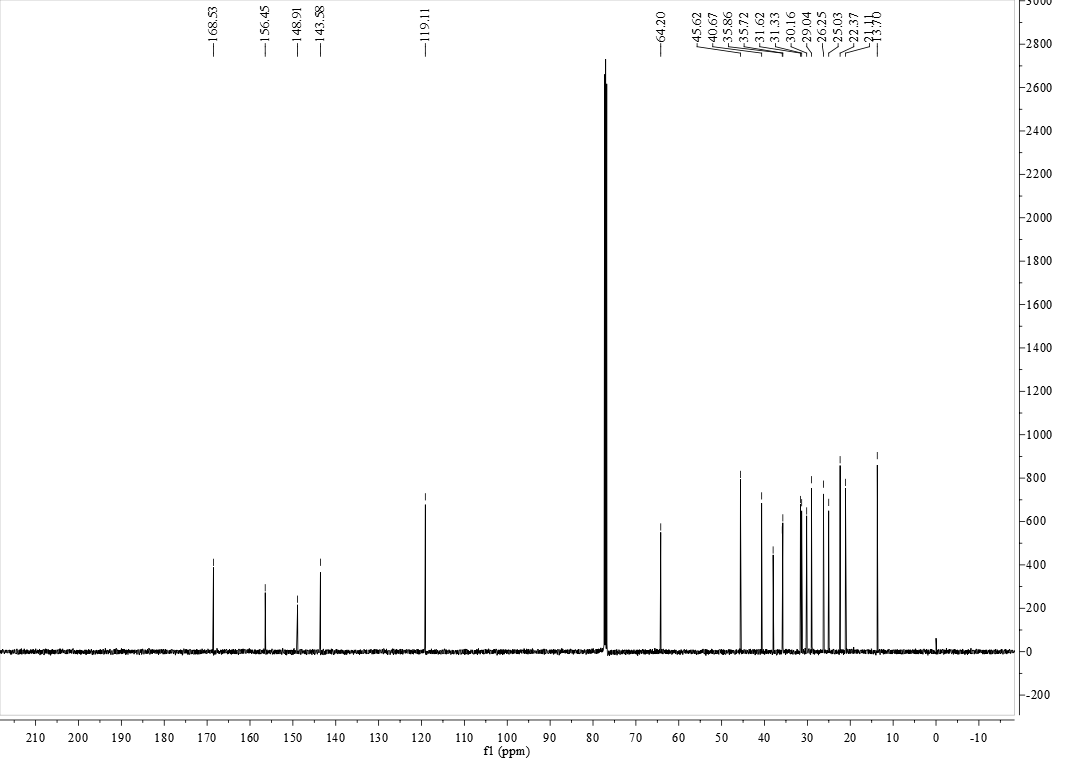


**FigureS50.** ^13^C NMR (125 MHz, CDCl_3_) of compound **5w**

**FT-IR-Spectra**


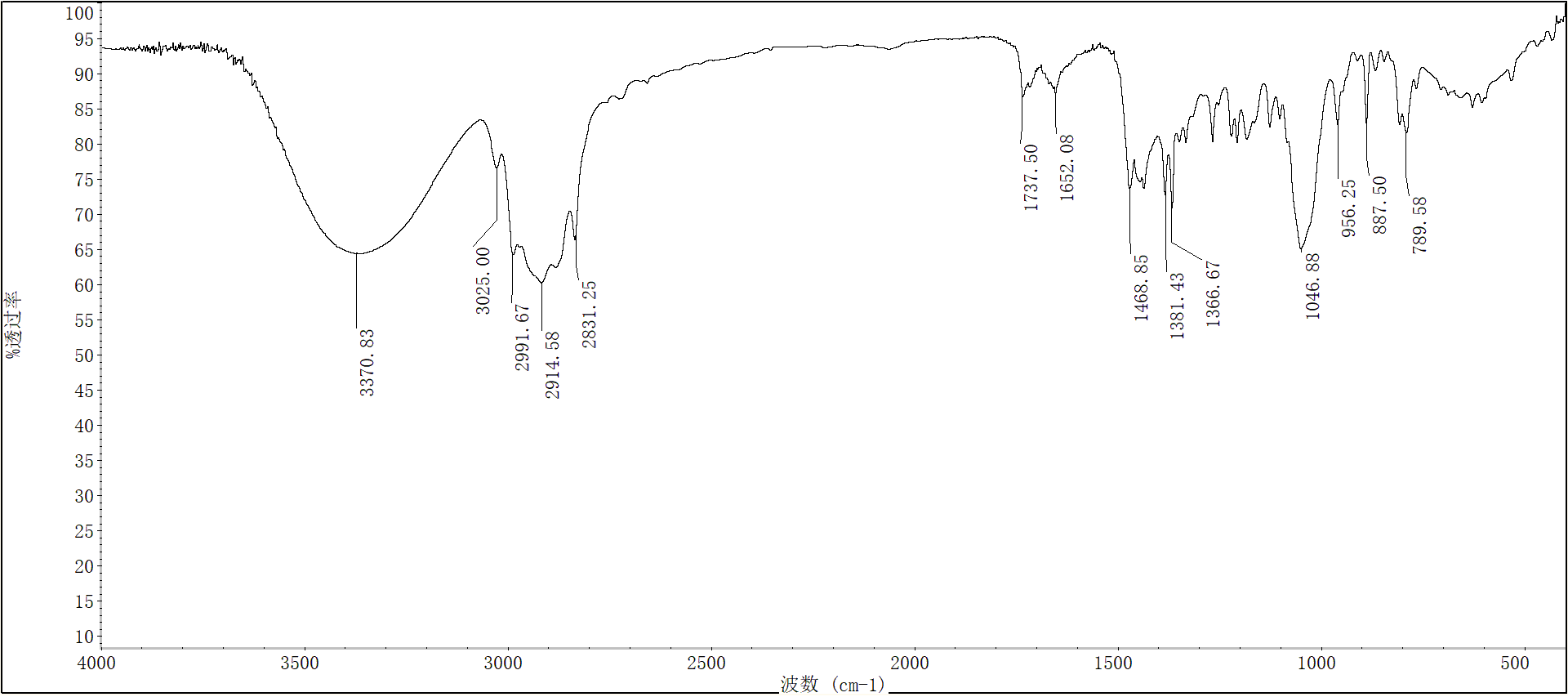


**FigureS51.** FT-IR spectra of compound **2**


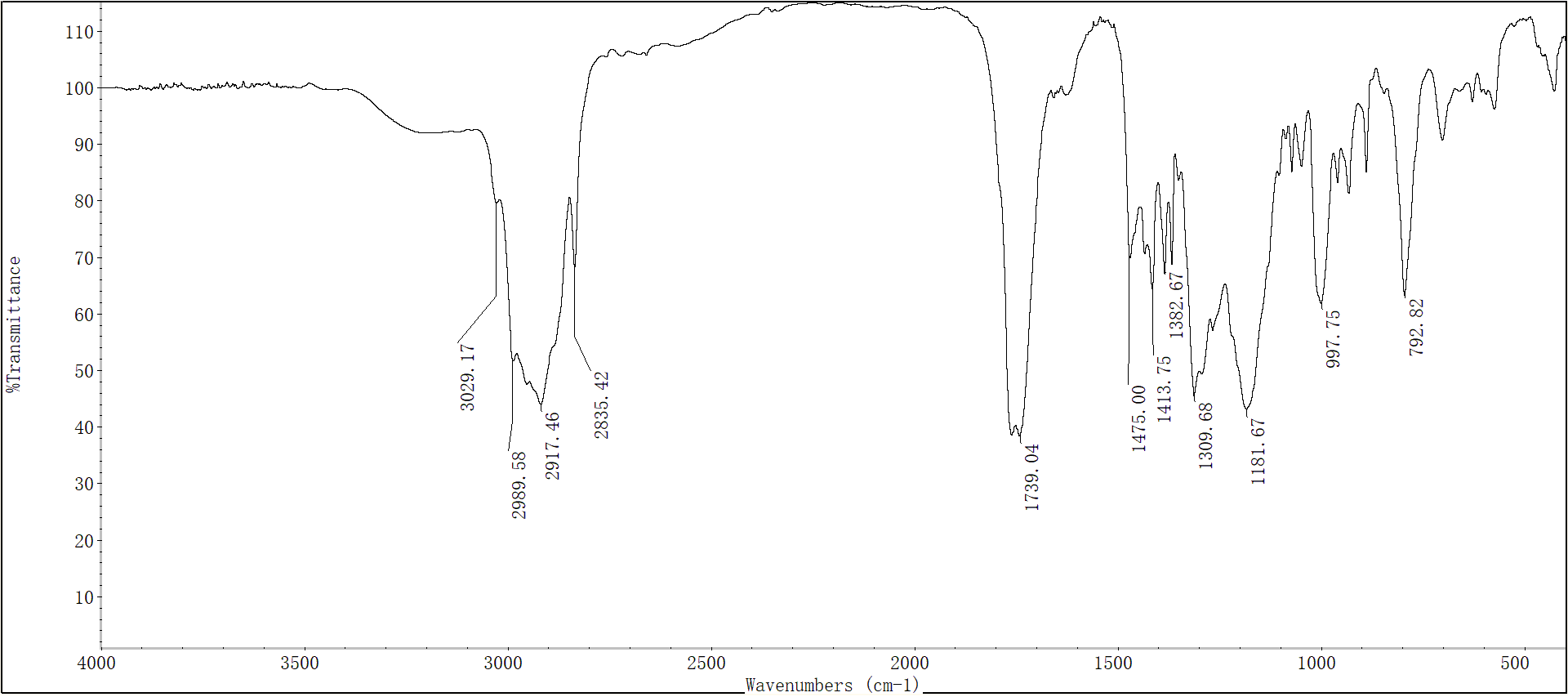


**FigureS52.** FT-IR spectra of compound **3**


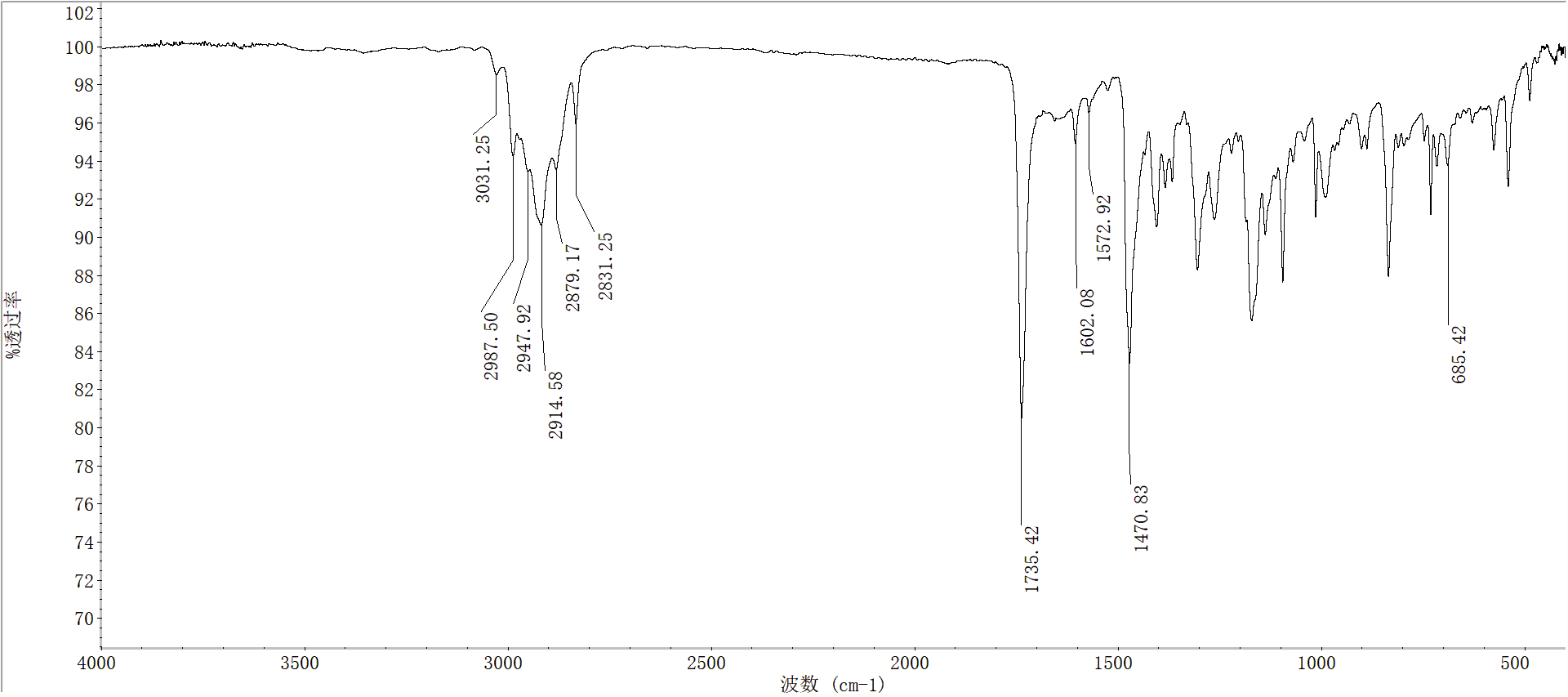


**FigureS53.** FT-IR spectra of compound **5a**


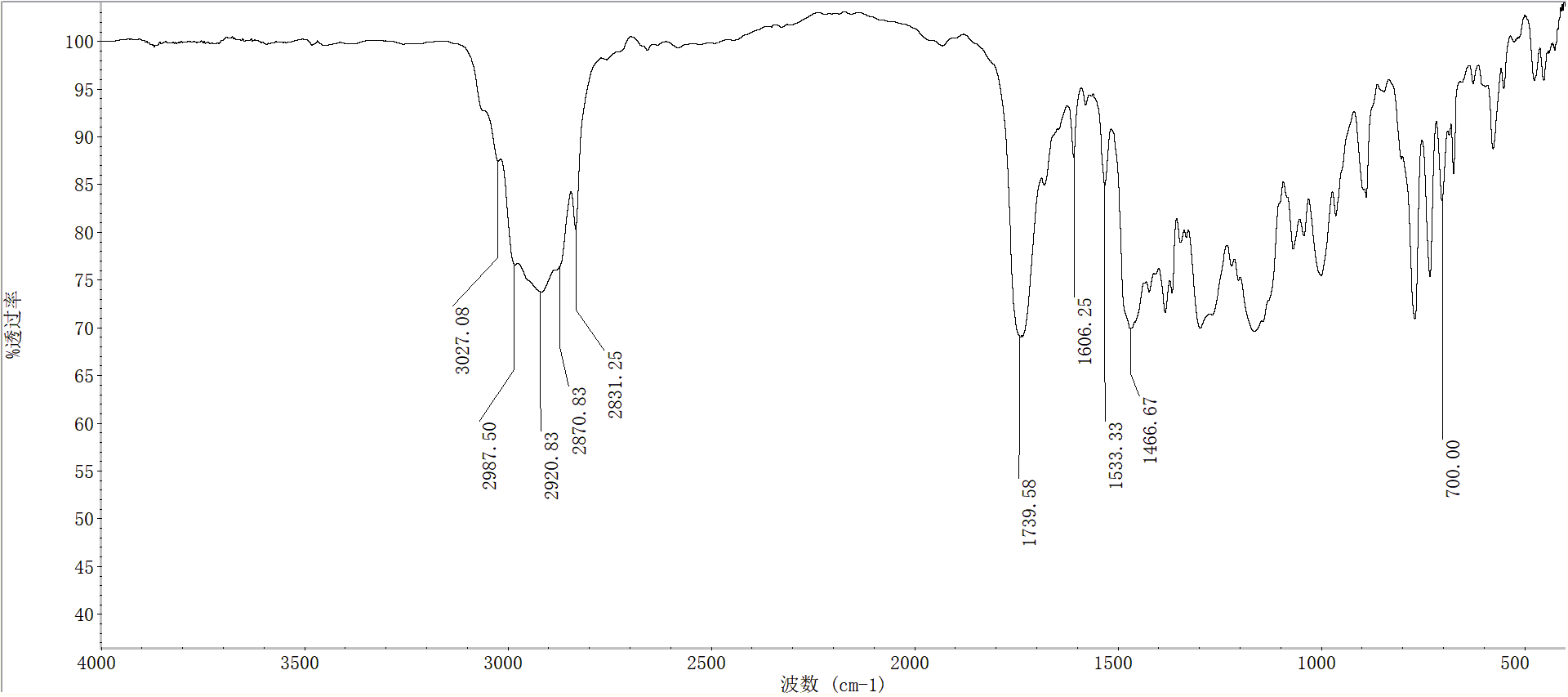


**FigureS54.** FT-IR spectra of compound **5b**


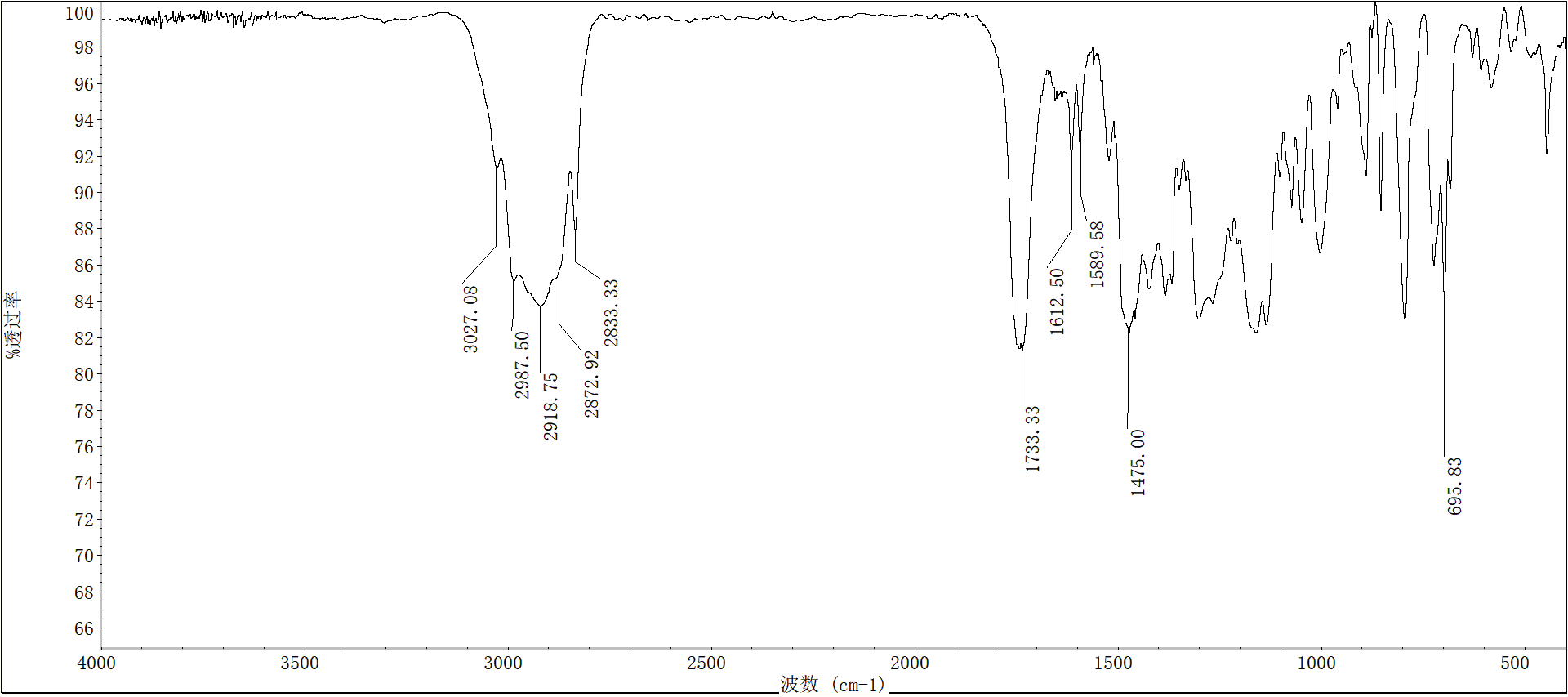


**FigureS55.** FT-IR spectra of compound **5c**


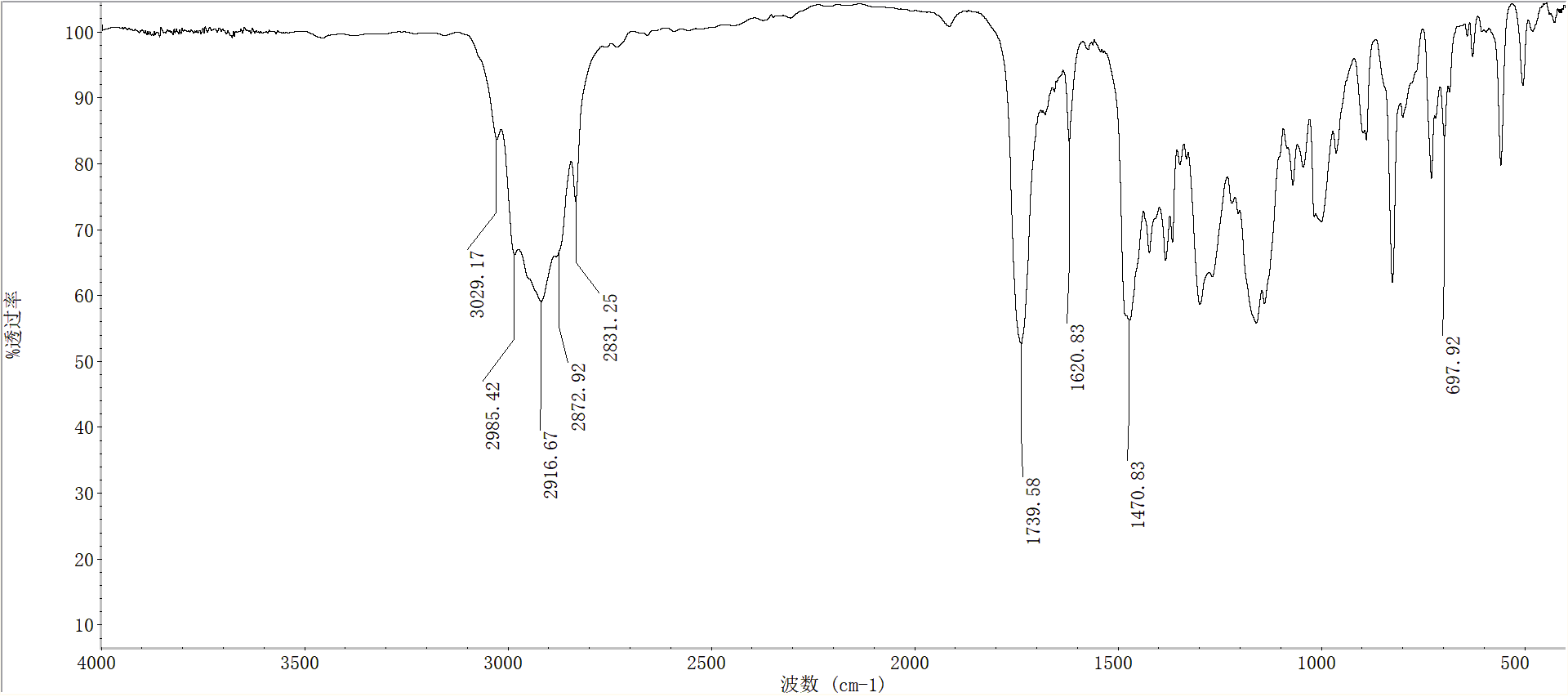


**FigureS56.** FT-IR spectra of compound **5d**


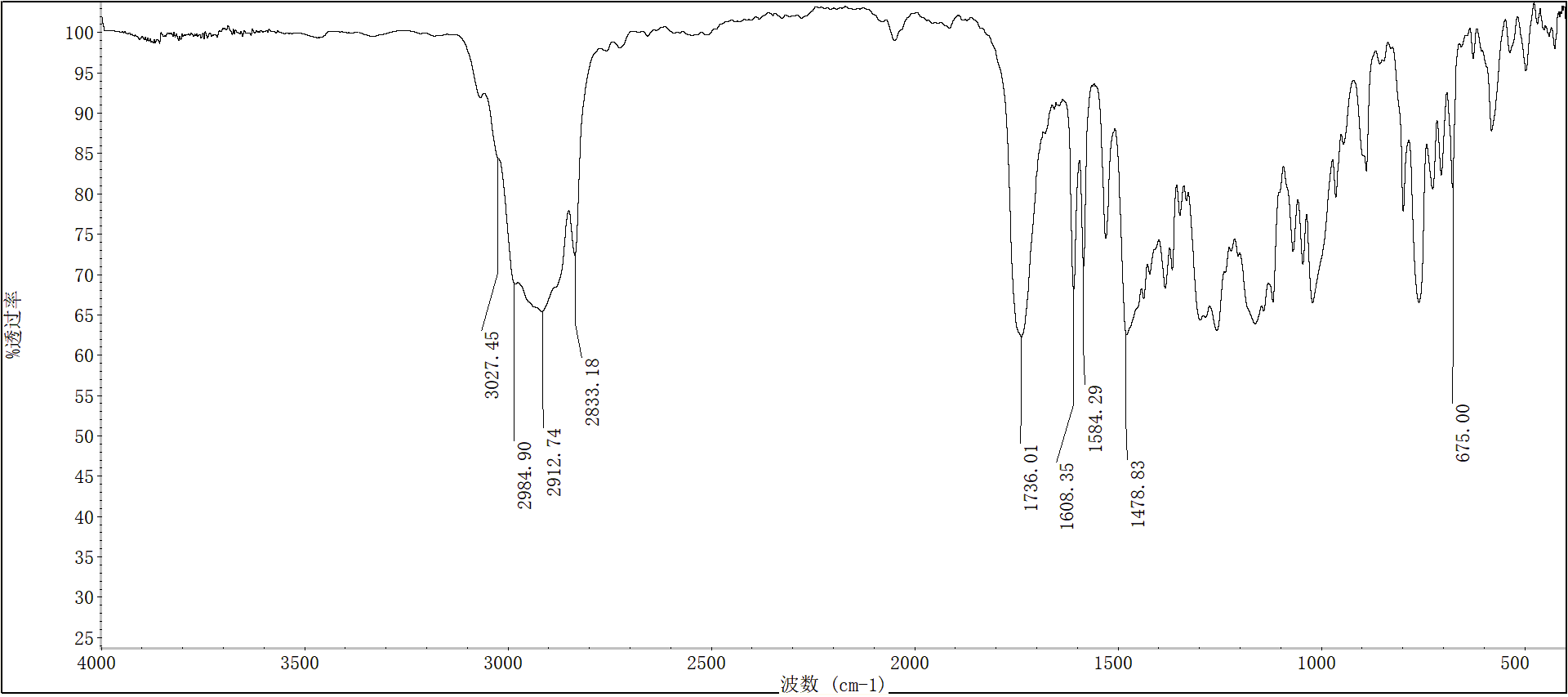


**FigureS57.** FT-IR spectra of compound **5e**


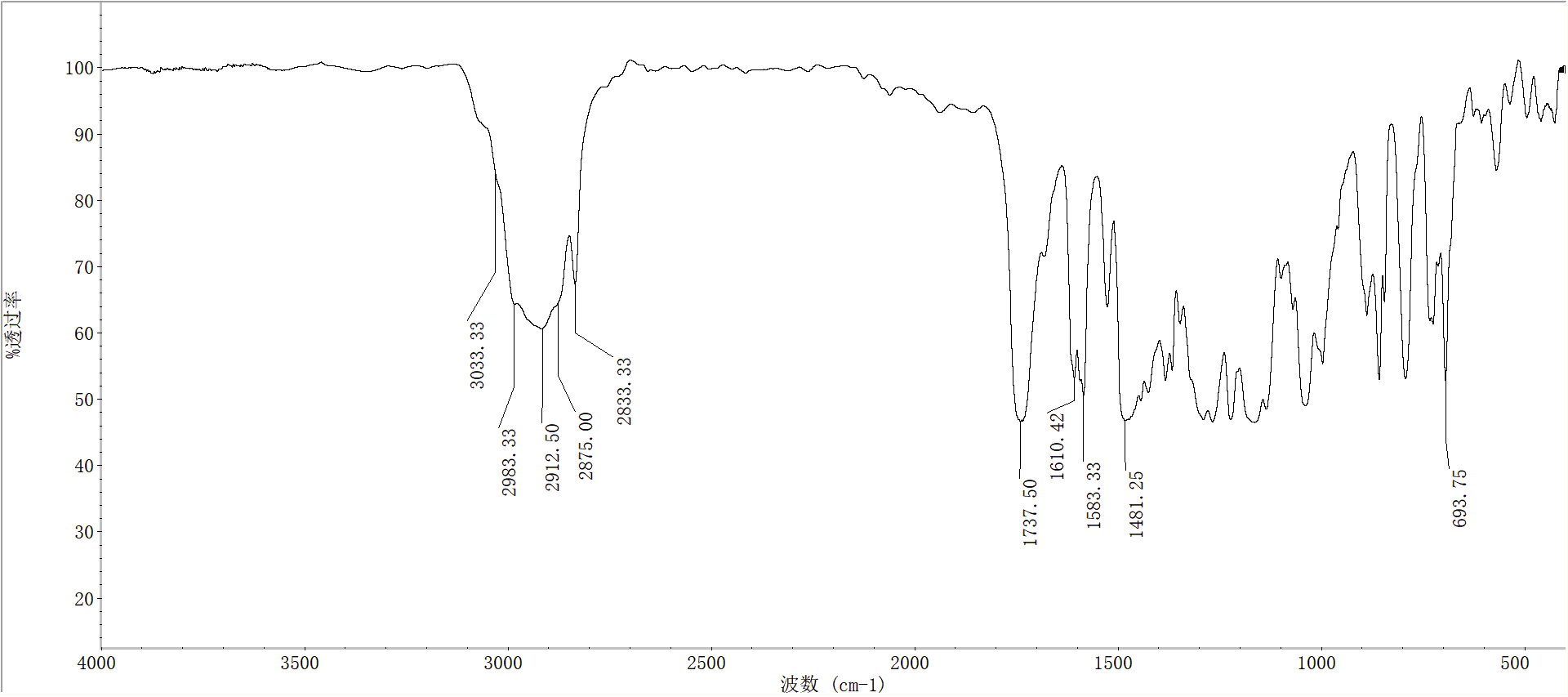


**FigureS58.** FT-IR spectra of compound **5f**


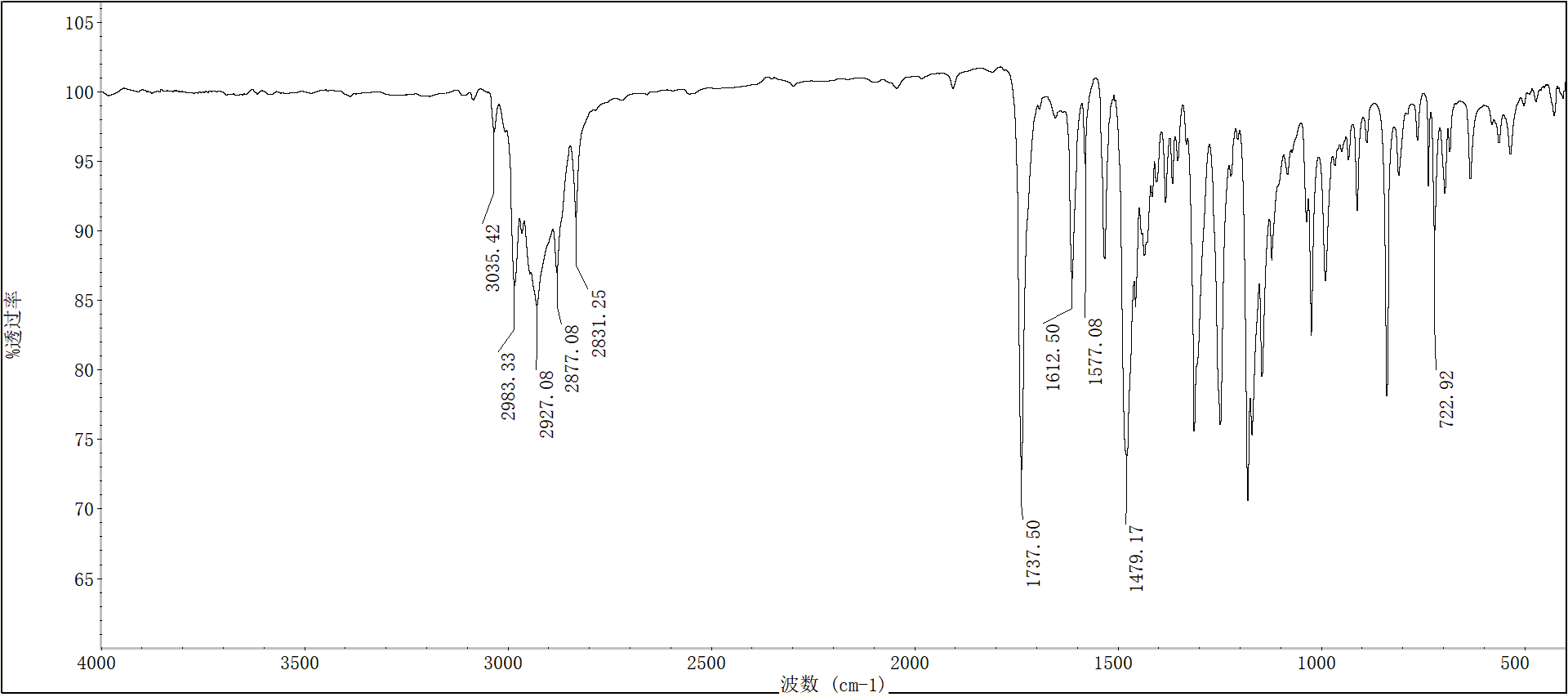


**FigureS59.** FT-IR spectra of compound **5g**


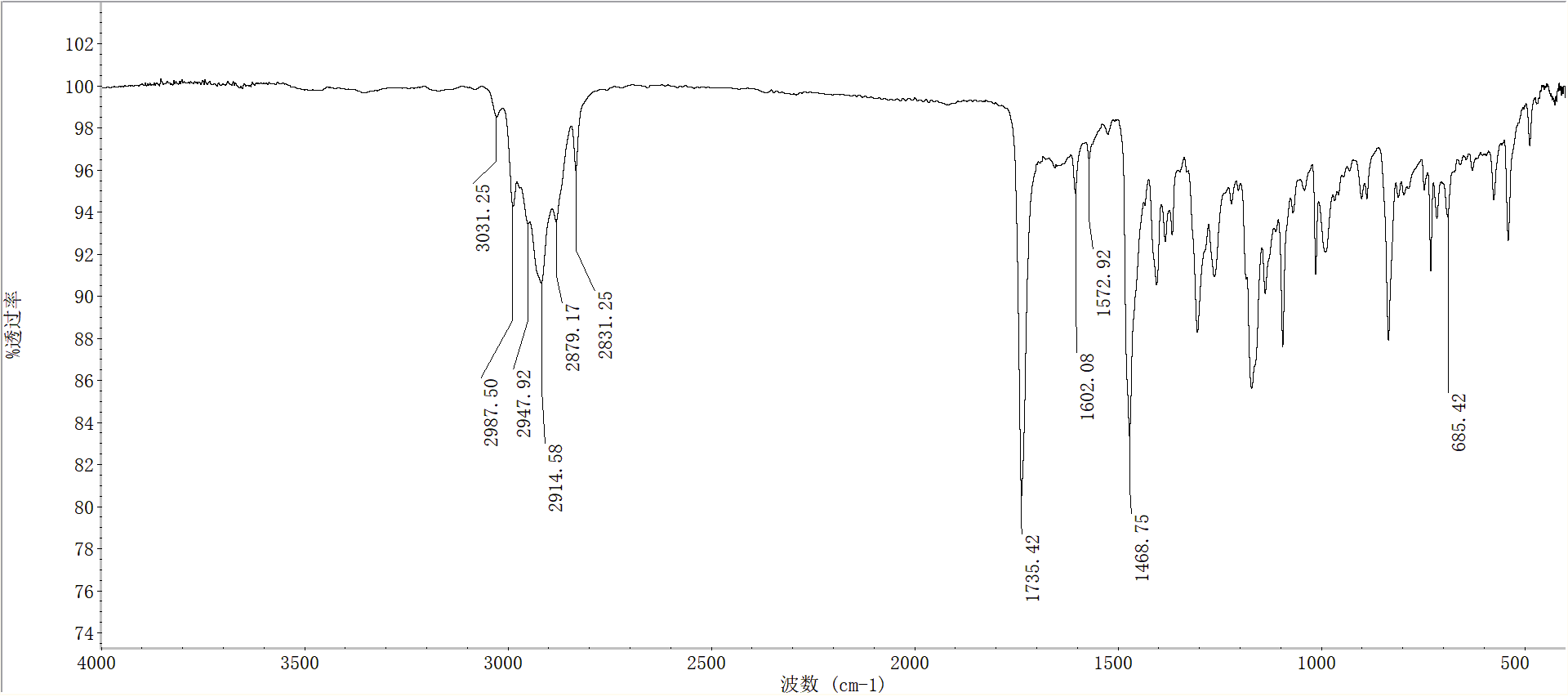


**FigureS60.** FT-IR spectra of compound **5h**


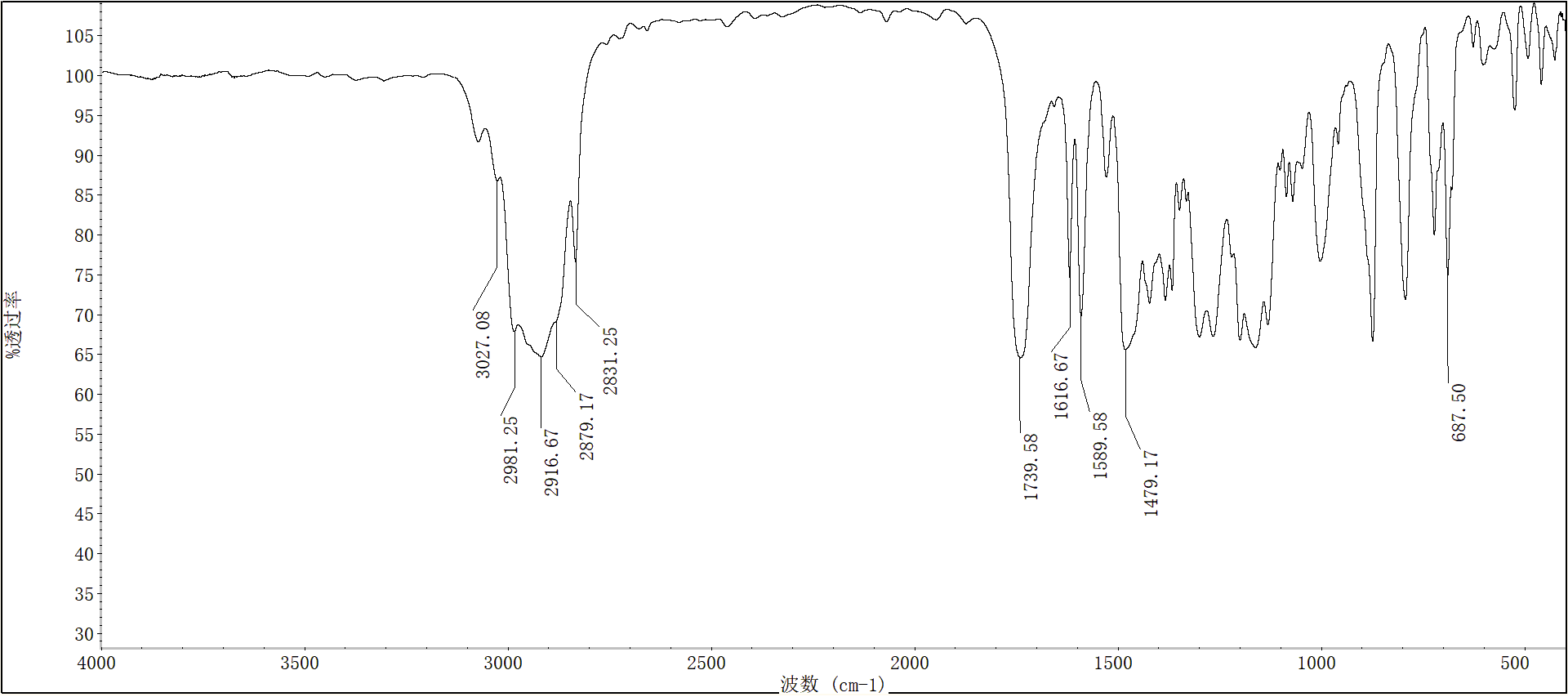


**FigureS61.** FT-IR spectra of compound **5i**


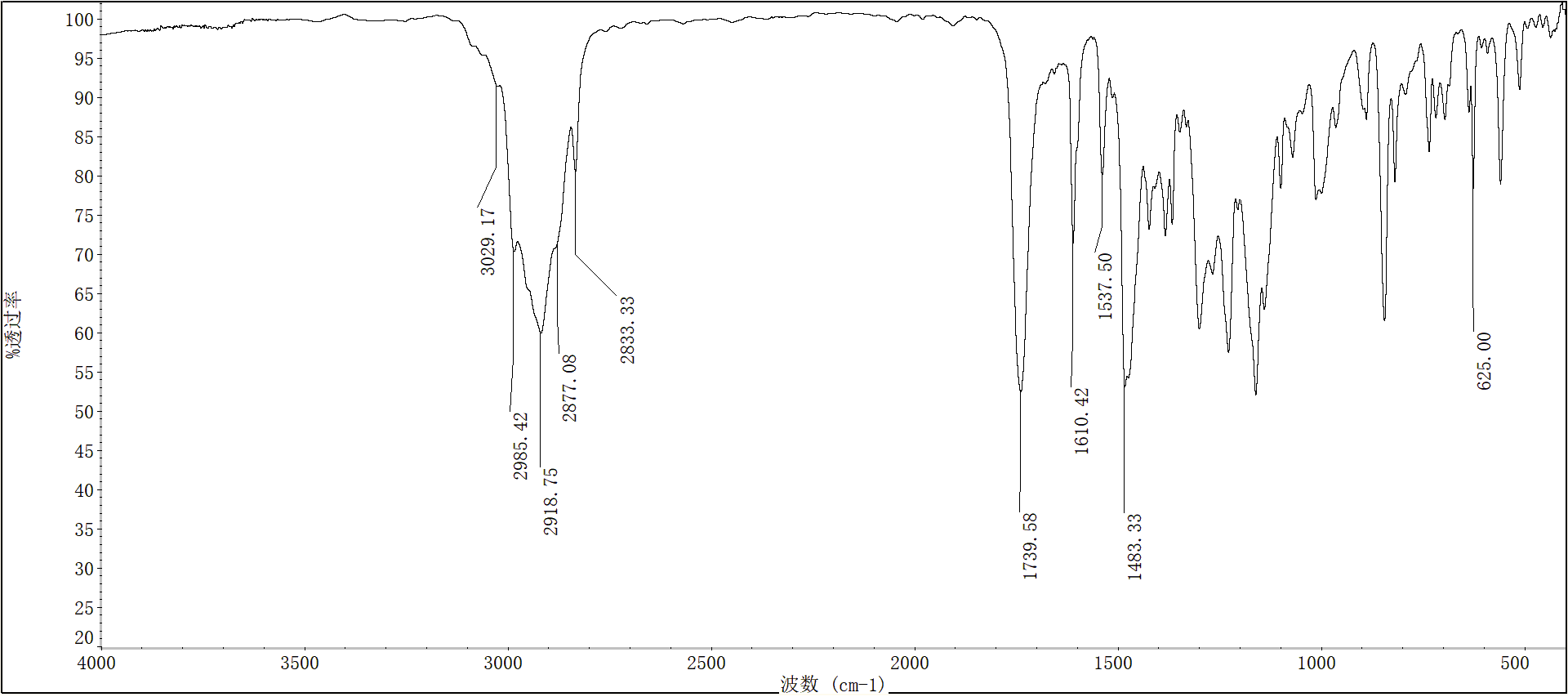


**FigureS62.** FT-IR spectra of compound **5j**


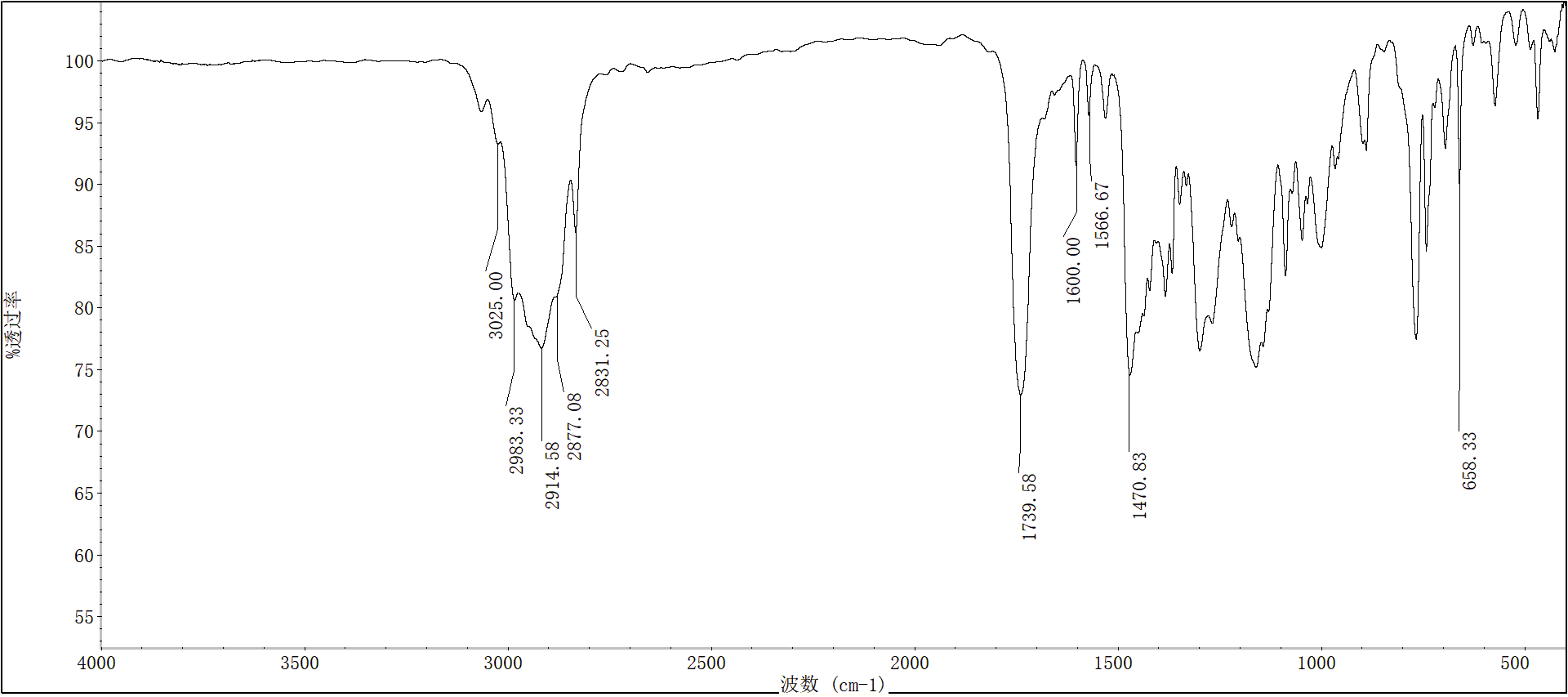


**FigureS63.** FT-IR spectra of compound **5k**


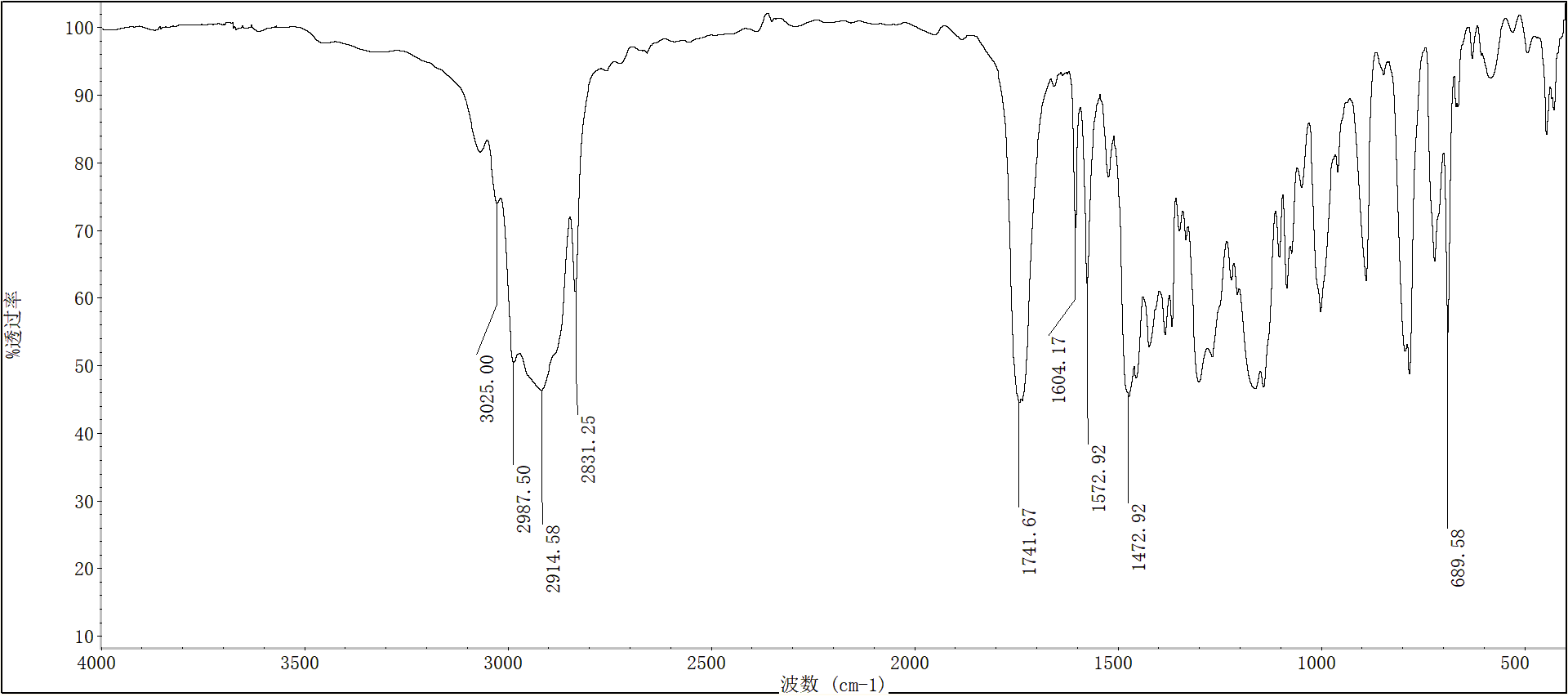


**FigureS64.** FT-IR spectra of compound **5l**


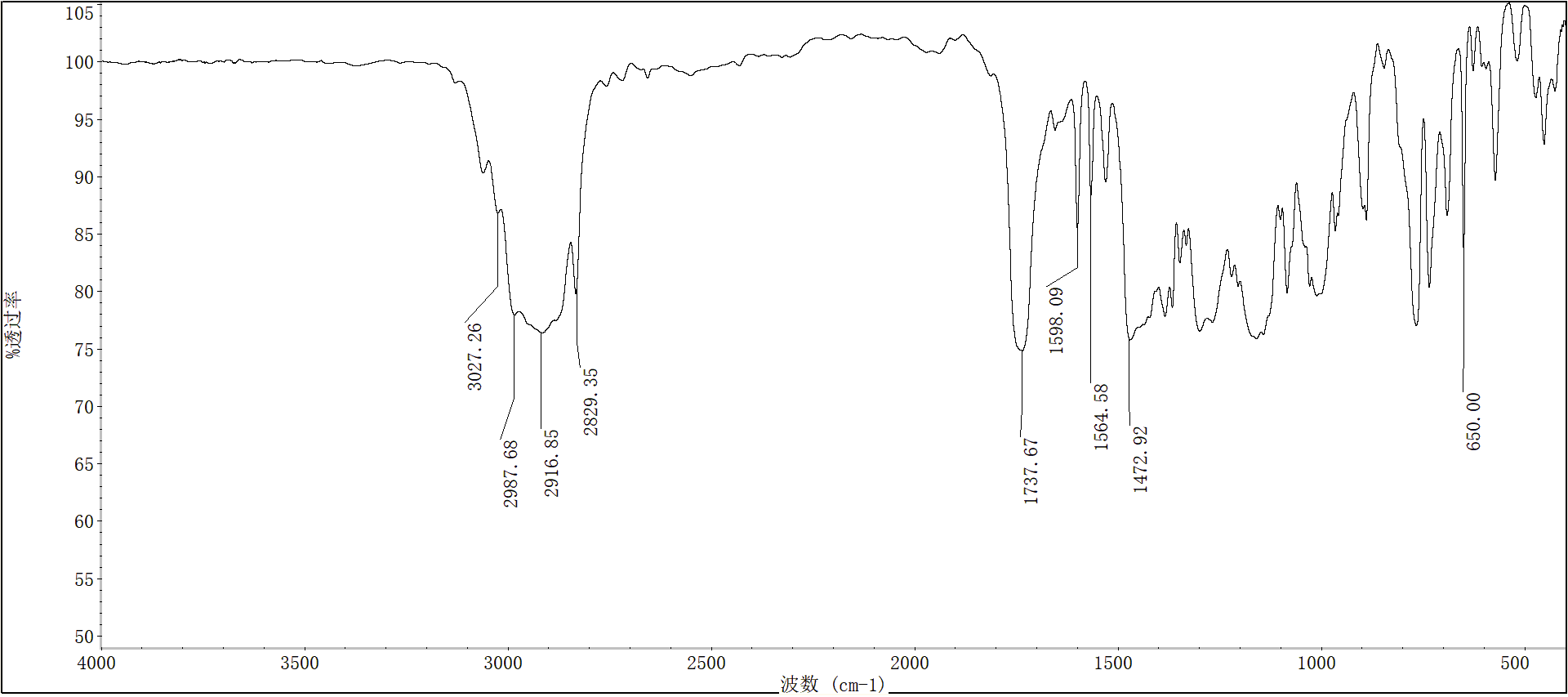


**FigureS65.** FT-IR spectra of compound **5m**


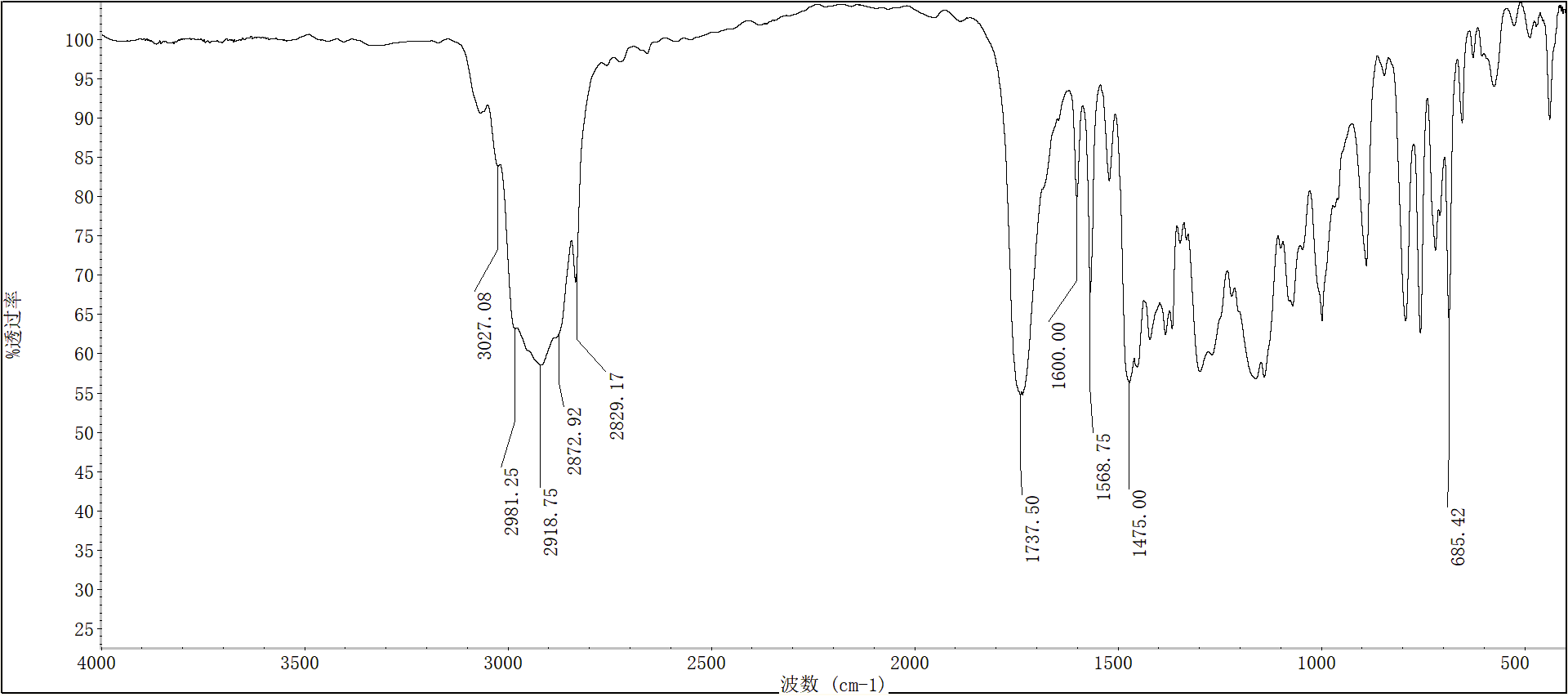


**FigureS66.** FT-IR spectra of compound **5n**


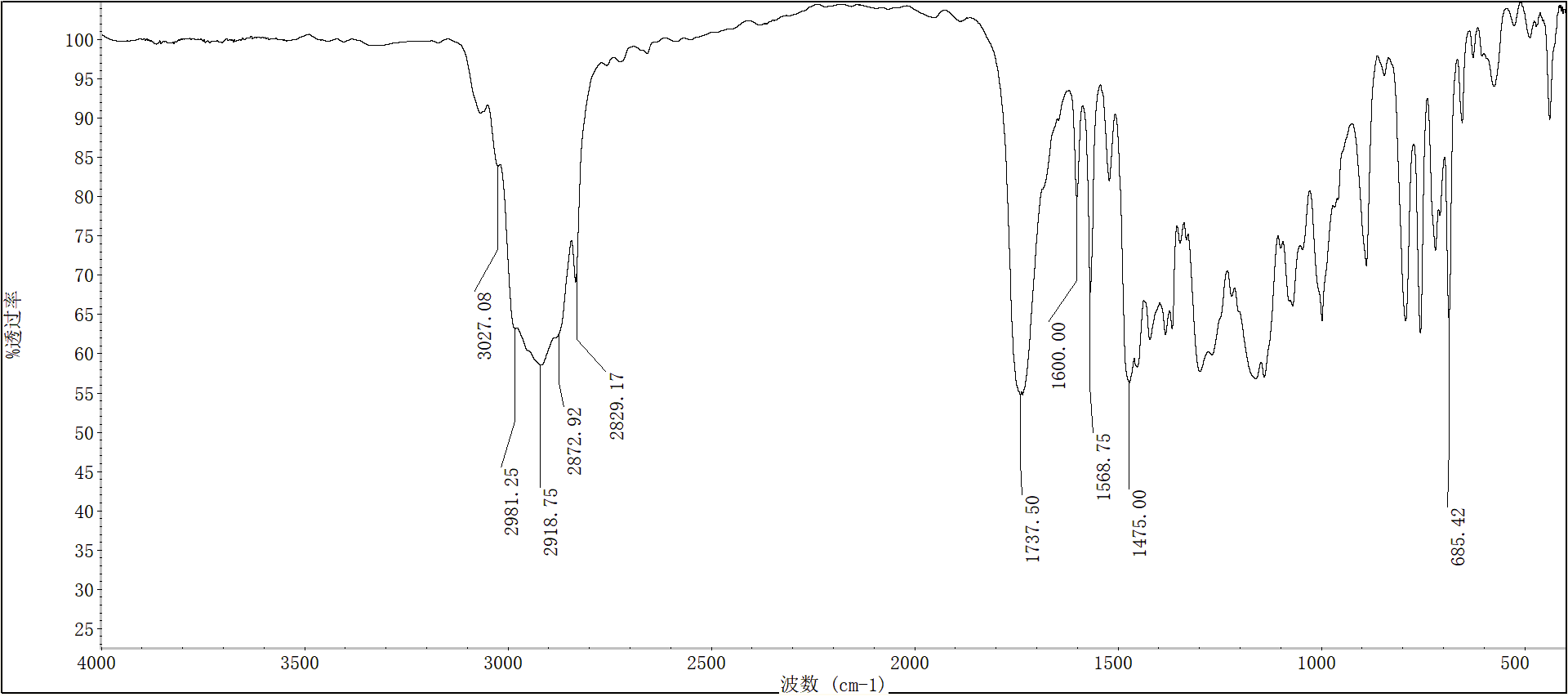


**FigureS67.** FT-IR spectra of compound **5o**


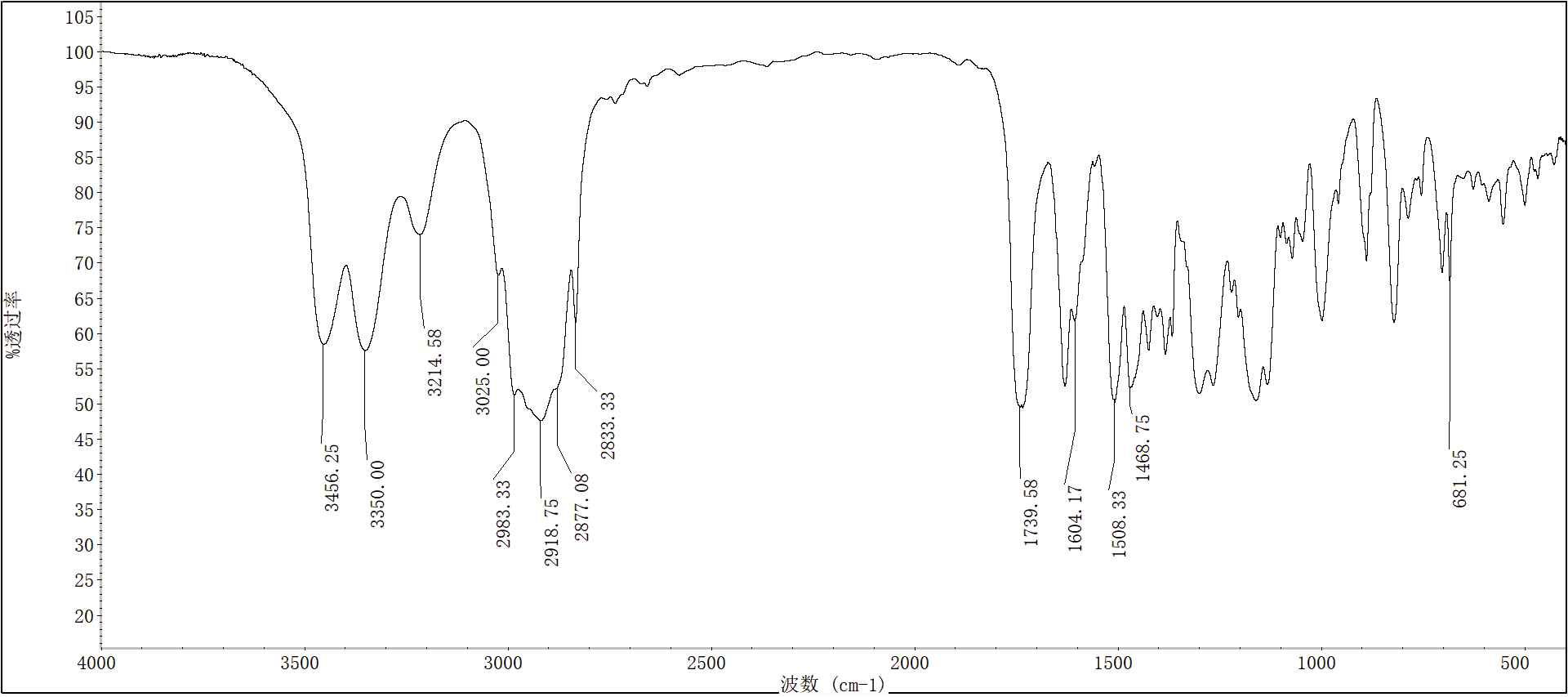


**FigureS68.** FT-IR spectra of compound **5p**


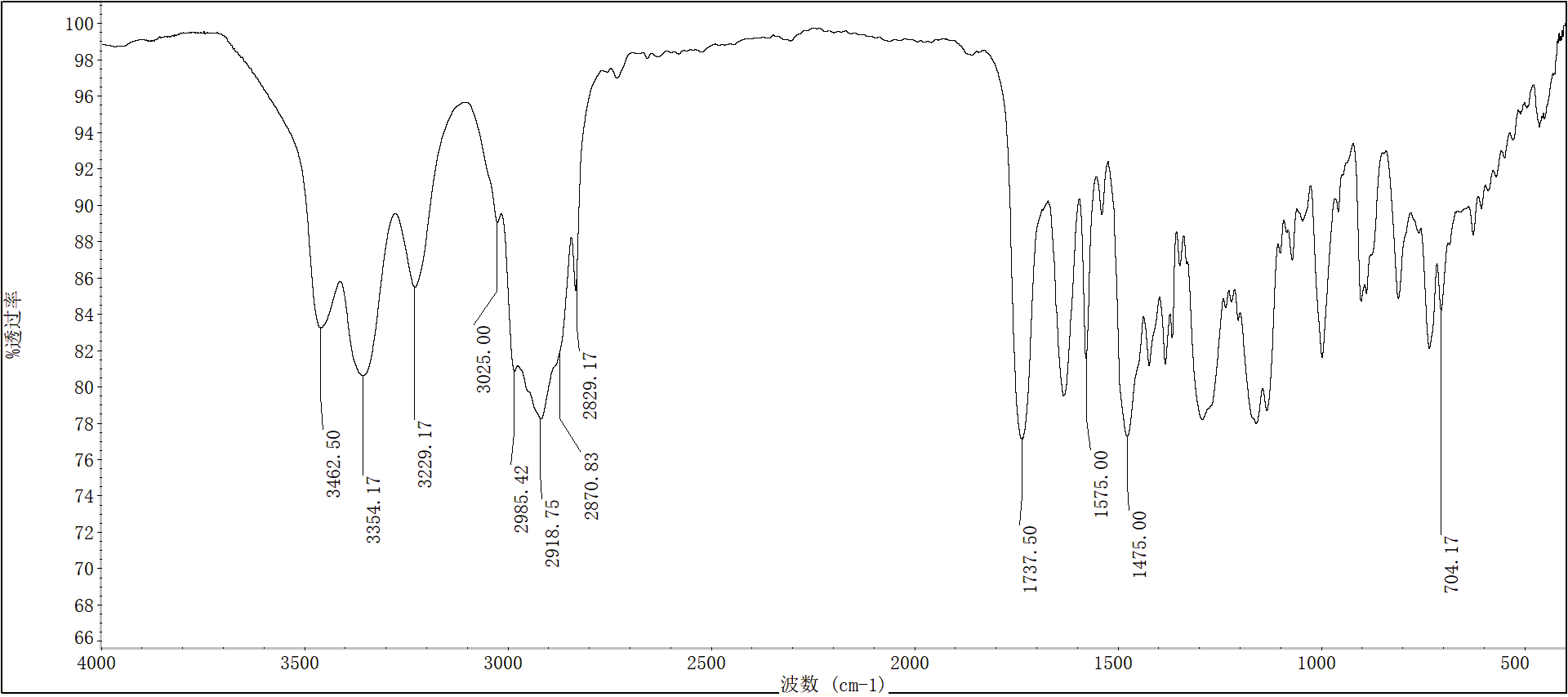


**FigureS69.** FT-IR spectra of compound **5q**


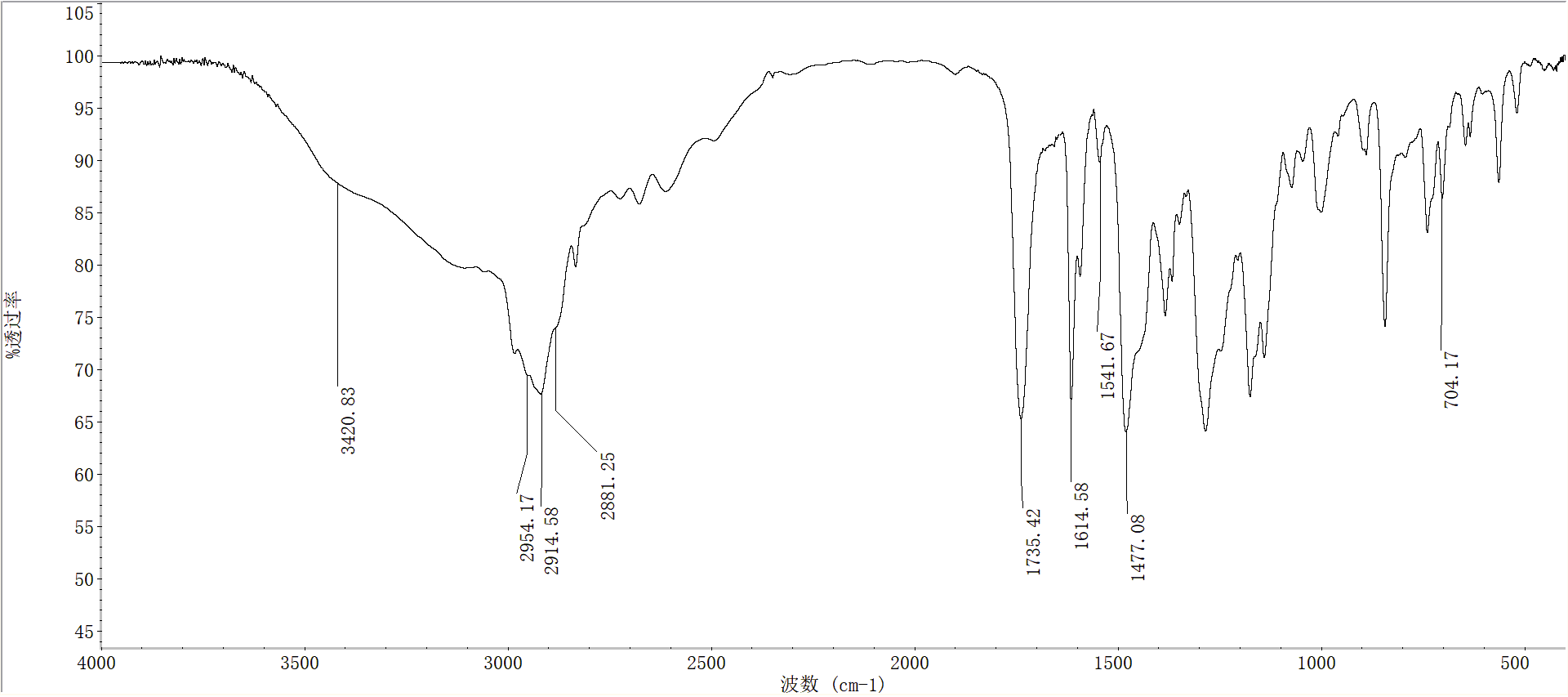


**FigureS70.** FT-IR spectra of compound **5r**


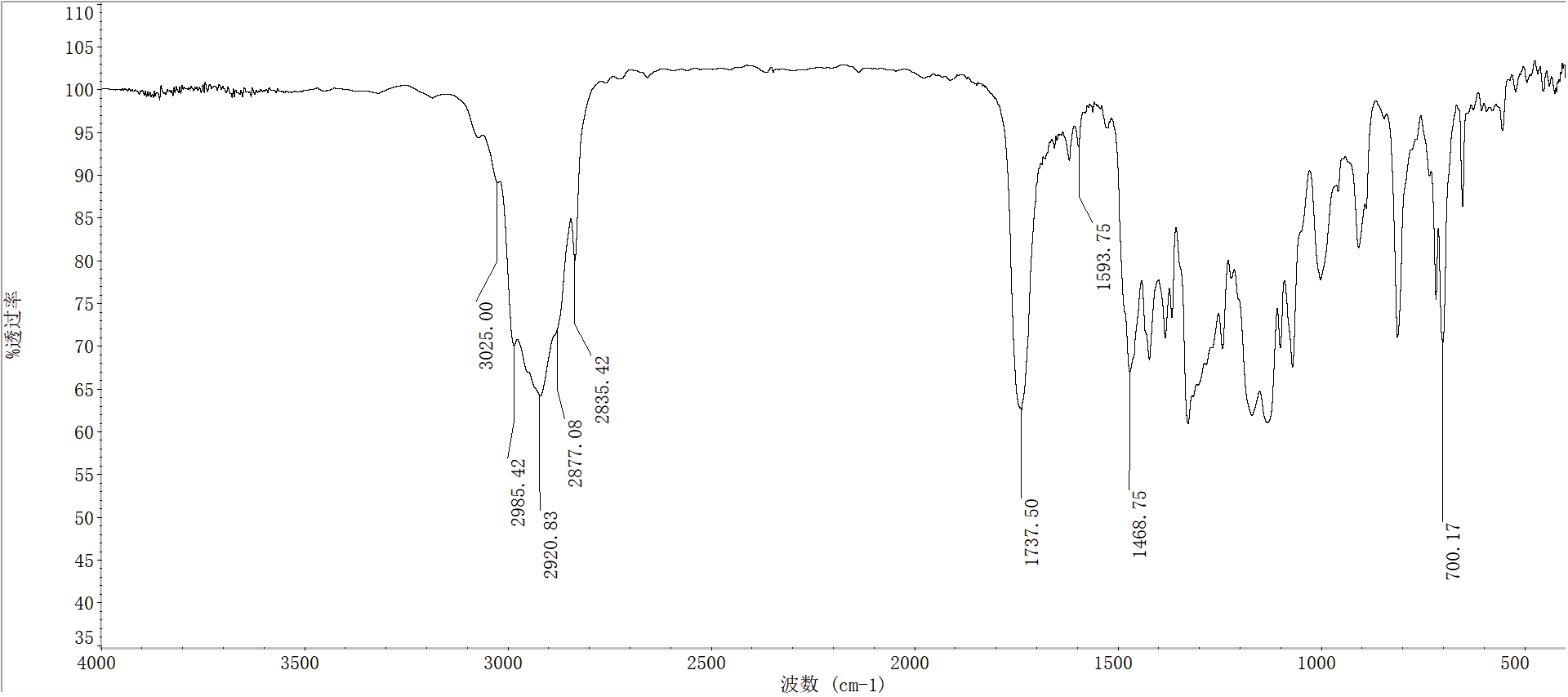


**FigureS71.** FT-IR spectra of compound **5s**


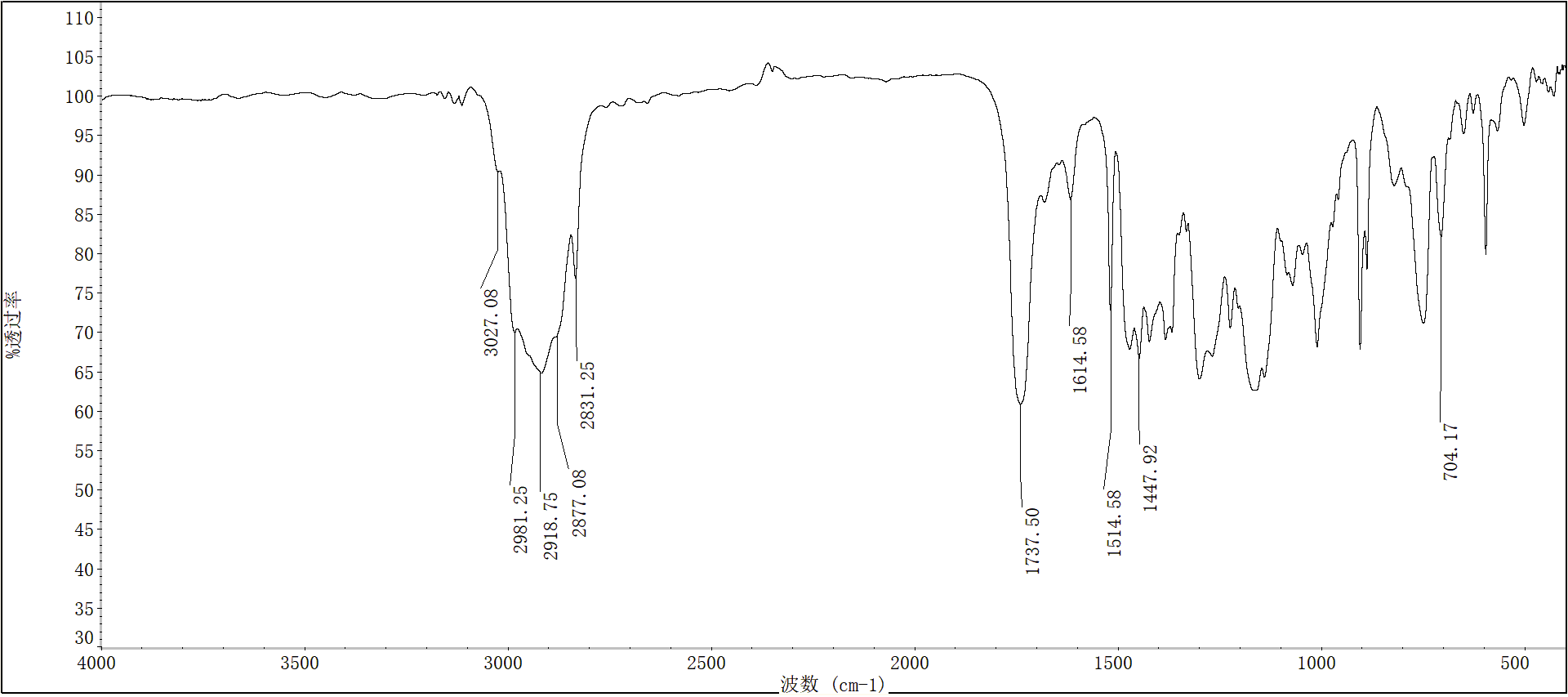


**FigureS72.** FT-IR spectra of compound **5t**


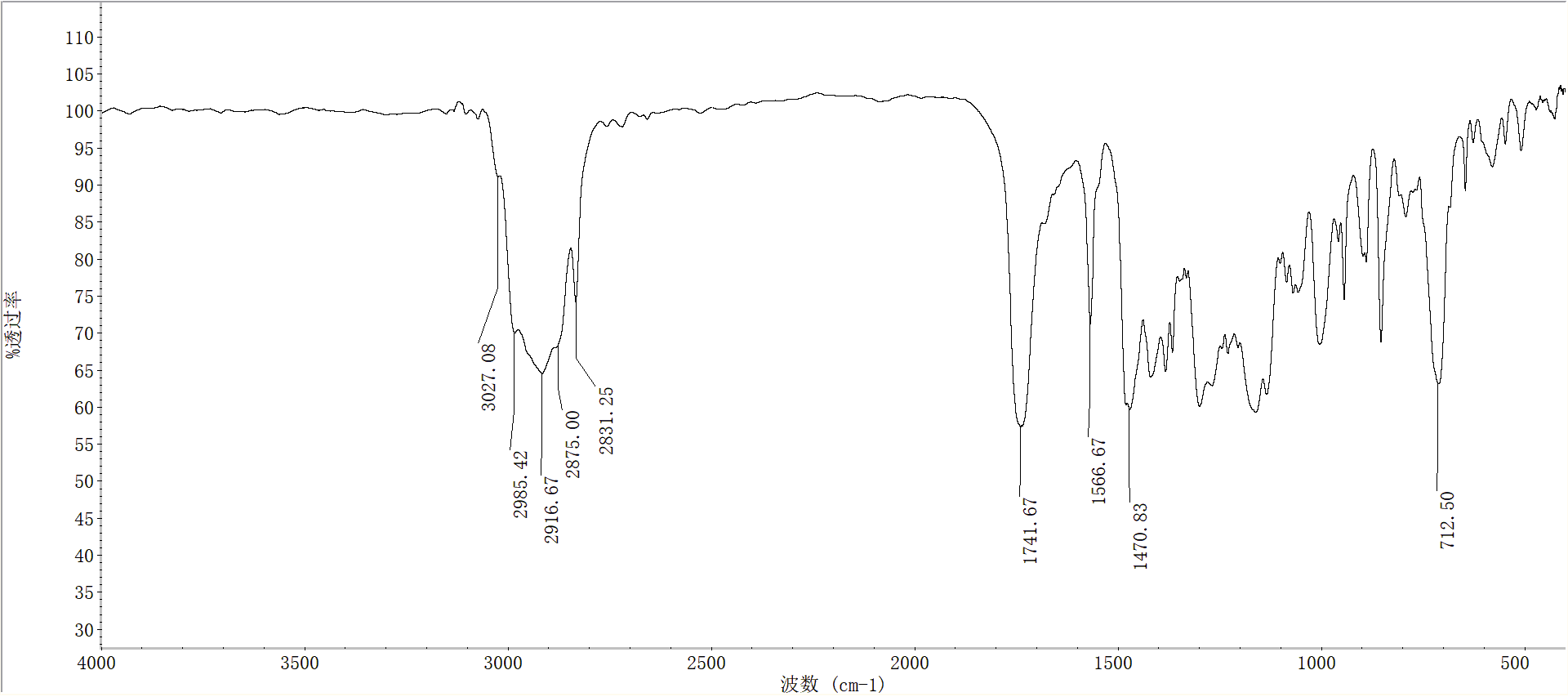


**FigureS73.** FT-IR spectra of compound **5u**


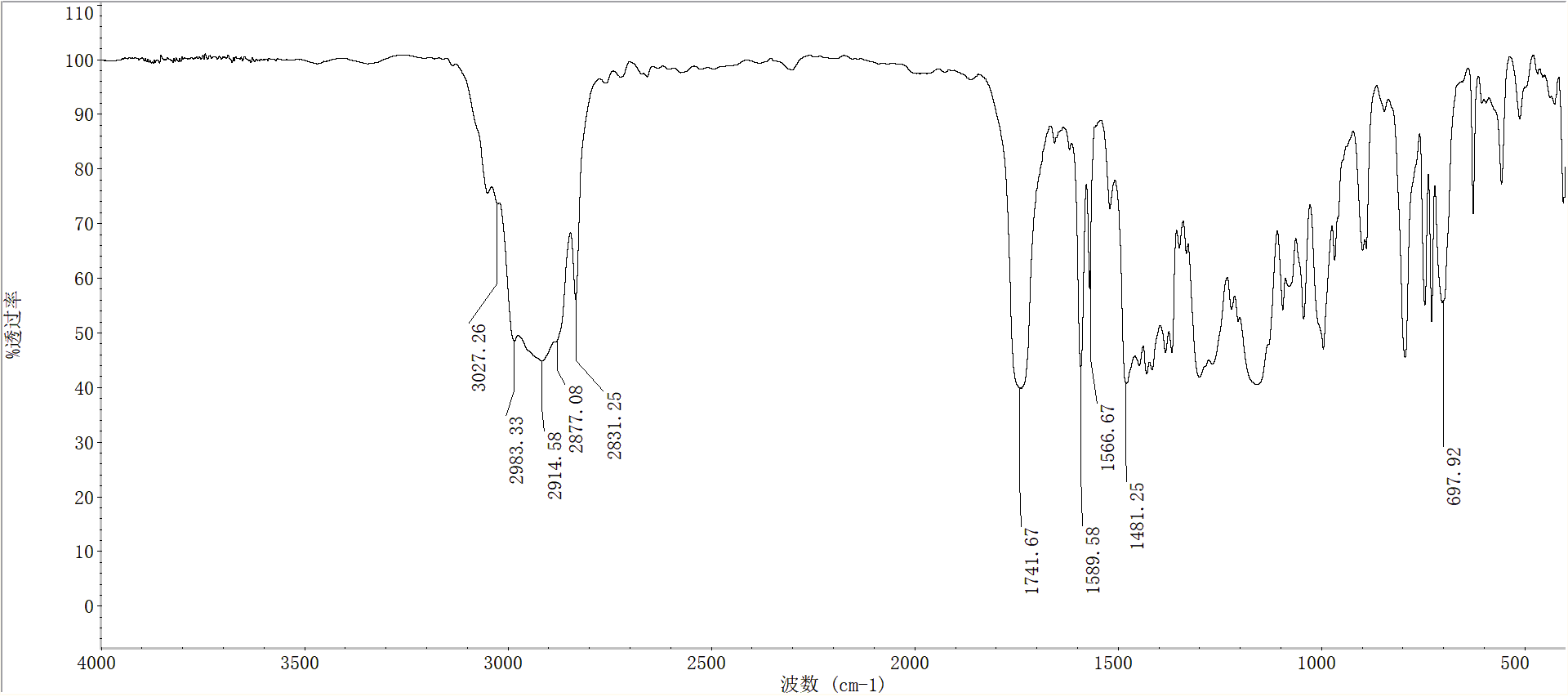


**FigureS74.** FT-IR spectra of compound **5v**


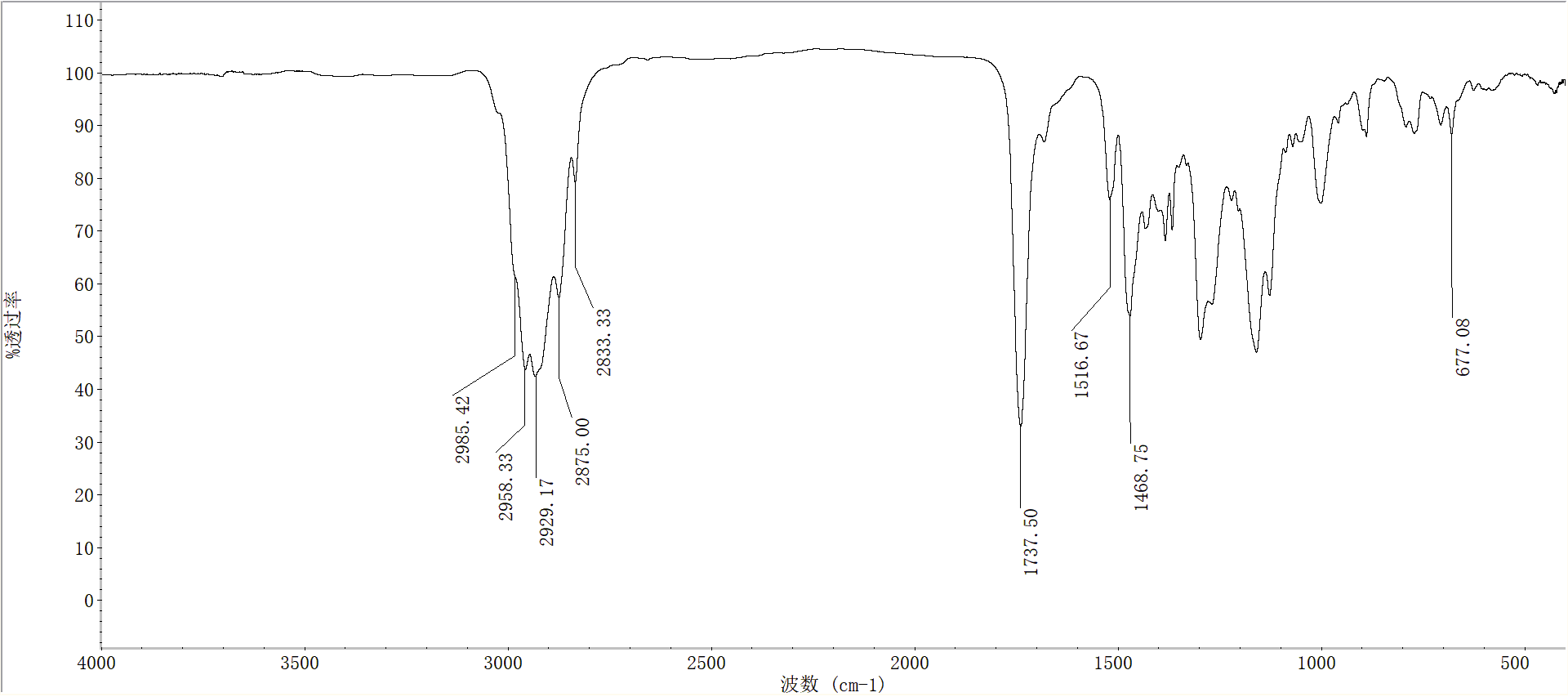


**FigureS75.** FT-IR spectra of compound **5w**

**MS-Spectra**

**FigureS76.** MS spectra of compound **5a**

**FigureS77.** MS spectra of compound **5b**

**FigureS78.** MS spectra of compound **5c**

**FigureS79.** MS spectra of compound **5d**

**FigureS80.** MS spectra of compound **5e**

**FigureS81.** MS spectra of compound **5f**

**FigureS82.** MS spectra of compound **5g**

**FigureS83.** MS spectra of compound **5h**

**FigureS84.** MS spectra of compound **5i**

**FigureS85.** MS spectra of compound **5j**

**FigureS86.** MS spectra of compound **5k**

**FigureS87.** MS spectra of compound **5l**

**FigureS88.** MS spectra of compound **5m**

**FigureS89.** MS spectra of compound **5n**

**FigureS90.** MS spectra of compound **5o**

**FigureS91.** MS spectra of compound **5p**

**FigureS92.** MS spectra of compound **5q**

**FigureS93.** MS spectra of compound **5r**

**FigureS94.** MS spectra of compound **5s**

**FigureS95.** MS spectra of compound **5t**

**FigureS96.** MS spectra of compound **5u**

**FigureS97.** MS spectra of compound **5v**

**FigureS98.** MS spectra of compound **5w**

**
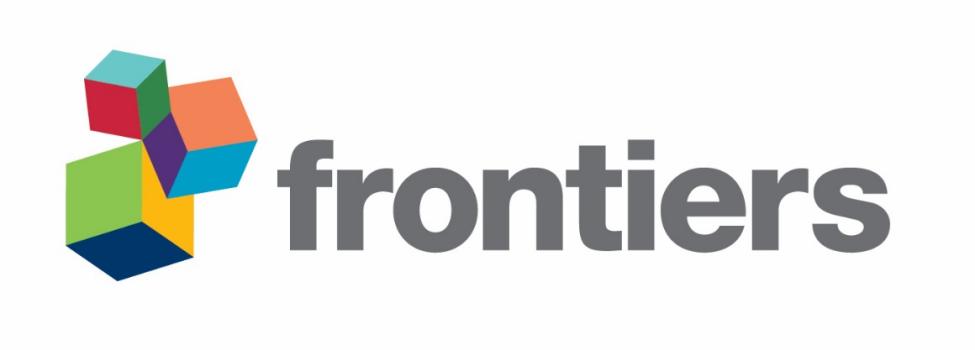
**
